# Development of a Smart Fluorescent Probe Specifically Interacting with C-Myc I-Motif

**DOI:** 10.3390/ijms23073872

**Published:** 2022-03-31

**Authors:** Zuzhuang Wei, Bobo Liu, Xiaomin Lin, Jing Wang, Zhi-Shu Huang, Ding Li

**Affiliations:** School of Pharmaceutical Sciences, Sun Yat-sen University, 132 Waihuan East Road, Guangzhou University City, Guangzhou 510006, China; weizzh@mail2.sysu.edu.cn (Z.W.); liubb9@mail2.sysu.edu.cn (B.L.); linxm35@mail2.sysu.edu.cn (X.L.); wangj349@mail2.sysu.edu.cn (J.W.); ceshzs@mail.sysu.edu.cn (Z.-S.H.)

**Keywords:** i-motif, carbazole, fluorescent probe, *c-myc*, oncogene promoter

## Abstract

I-motifs play key regulatory roles in biological processes, holding great potential as attractive therapeutic targets. In the present study, we developed a novel fluorescent probe **G59** with strong and selective binding to the *c-myc* gene promoter i-motif. **G59** had an i-motif-binding carbazole moiety conjugated with naphthalimide fluorescent groups. **G59** could differentiate the *c-myc* i-motif from other DNA structures through selective activation of its fluorescence, with its apparent visualization in solution. The smart probe **G59** showed excellent sensitivity, with a low fluorescent detection limit of 154 nM and effective stabilization to the *c-myc* i-motif. **G59** could serve as a rapid and sensitive probe for label-free screening of selective *c-myc* i-motif binding ligands under neutral crowding conditions. To the best of our knowledge, **G59** is the first fluorescent probe with high sensitivity for recognizing the i-motif structure and screening for selective binding ligands.

## 1. Introduction

Besides the well-known duplex structure, DNA may self-assemble to form noncanonical secondary structures, such as triplexes and quadruplexes [1]. Among these structures, the i-motif is one of the most important four-stranded DNA secondary structures, which was first recognized in 1993 [2]. I-motif structures are hemiprotonated species with cytosine-rich sequences, which are formed through a pair of parallel duplexes with intercalated C–C^+^ base pairs. A slightly acidic pH environment favors i-motif formation, due to the protonation of N_3_ in cytosine. In the presence of molecular crowding agents, negative superhelicity, or Ag^+^ cations, some i-motif structures could be stable under neutral or even slightly basic conditions [3]. In contrast to the G-quadruplex, the biological function of the i-motif has been much less studied, although these two structures are equally important in vivo. Recent studies have suggested that i-motif structures extensively exist in the human genome, especially in telomeric regions, centromeres, and oncogene promoter regions (*c-myc*, *bcl-2*, *c-kit*, etc.) [1]. These studies clearly indicate their biologically important roles in oncogene transcriptional regulation, which may be associated with important diseases, including cancer [4]. It should be noted that NMR experiments have revealed i-motif structure formation in cells [5], and, more importantly, antibodies (iMab) have recognized the i-motif structures with high affinity in the nuclei of human living cells [6]. The i-motif structures are gaining increasing interest, due to their peculiar architecture and biological functions, as well as their extreme pH dependency, which can be widely used for disease diagnosis and therapeutics [7]. Consequently, the design and development of selective and fluorescent i-motif probes are significant for research on their biological functions in regulating cellular processes. Although some fluorescent molecules that are responsive to i-motif structures have been previously reported, including thiazole orange [8], crystal violet [9], [Ru (phen)_2_ (dppz)]^2+^ [10], [Ru (bqp)_2_]^2+^ [11], terbium (III)–platinum(II) complex [12], berberine [13], neutral red [14], CHE [15], thioflavin T [16,17], and iridium (III) complex [18], these compounds could also interact with other DNA structures, making it impossible to sense i-motif structures selectively. In addition, their syntheses are relatively complicated, and some require additional labelling. Thus, the development of a selective and fluorescent i-motif probe is urgently required, to reveal and track i-motif formation dynamics for further study of their roles.

The promoter element of the *c-myc* oncogene is an important regulator of cellular proliferation and differentiation, and is considered to be one of the hallmarks of many types of human cancers [19]. It is considered to be a high-priority molecular target, and plays a crucial function in cell division and apoptosis. Its promoter C-rich strand can fold into an i-motif structure in slightly acidic conditions, and its stabilizing ligands can regulate gene transcription and translation [20,21]. Therefore, further development of a novel fluorescent probe that can selectively bind to the *c-myc* gene promoter i-motif could be significant, as a powerful research tool for tracking and investigating the i-motif’s biological functions. It has been shown that the carbazole derivative **3be** could bind to the *c-myc* gene promoter i-motif [22]; however, it would exhibit a fluorescence-quenching property. Therefore, **3be** cannot be used as a fluorescent probe for further biological studies. In this research, based on the chemical structure of **3be**, we synthesized a series of potential i-motif probes, including **G49**, **G50**, **G51**, and **G59–G67**. Interestingly, **G59** showed high sensitivity and selectivity to the *c-myc* gene promoter i-motif, with significantly enhanced fluorescence intensity, and minimum response to other DNA structures, indicating its significant application in differentiating the *c-myc* i-motif from other types of DNA structures. **G59** required no additional labeling for fluorescent detection of the *c-myc* i-motif, which could be further modified and developed as a promising fluorescent probe for applications in biological systems.

## 2. Results

### 2.1. Design and Syntheses of Carbazole Derivatives as Potential I-Motif Fluorescent Probes

**Figure 1:**
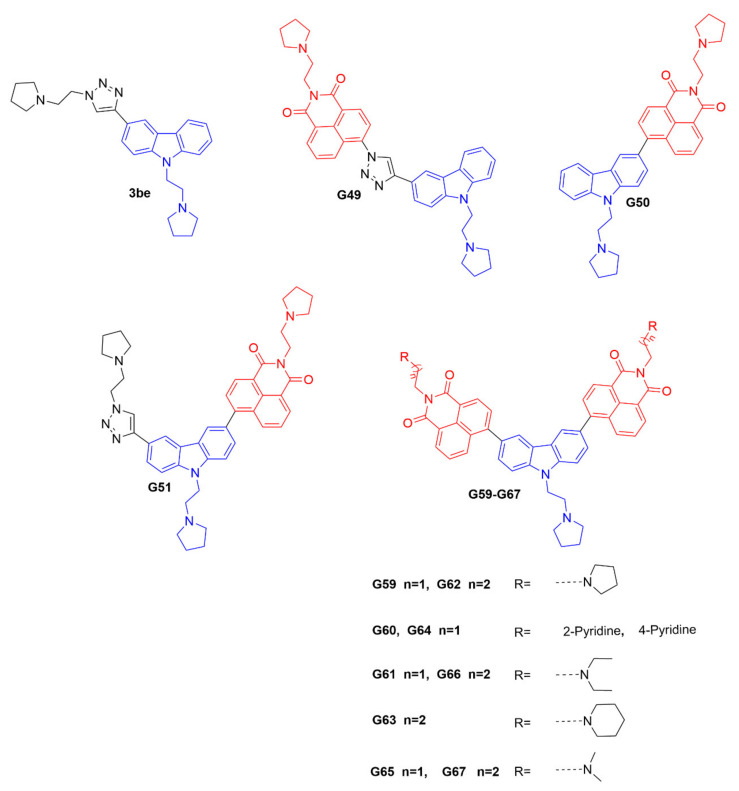
Chemical structures of **3be** and our carbazole derivatives as potential fluorescent probes.

The ideal fluorescent probe for the i-motif structure should have two essential features [23], including high recognition specificity and strong fluorescence intensity for target the i-motif. The carbazole derivative **3be** has been reported to bind with the *c-myc* gene promoter i-motif [22]; however, it has a fluorescence-quenching property that prevents it being used as a fluorescent probe. It is known that 1, 8-naphthalimide derivatives are strongly fluorescent, with a marked Stokes shift, and can be used for fluorescence sensing and imaging [24]. The 1, 8-naphthalimide derivatives could be connected with a DNA-binding pharmacophore, as fluorescent probes to track and investigate the biological functions of DNA secondary structures. Hence, in this study, based on the structure of the compound **3be**, twelve novel carbazole derivatives, as potential i-motif fluorescent probes, including **G49**, **G50**, **G51**, and **G59-G67,** were designed and synthesized, as shown in Figure 1 and Scheme S1A–D (Supplementary Materials). These compounds were designed with an i-motif-binding carbazole moiety, conjugated with naphthalimide at different positions, as potential fluorescent chemosensors, which were different from that previously reported for the acridone derivative **WZZ02**, with non-conjugated naphthalimide [25]. All the probes were characterized with ^1^H NMR, ^13^C NMR, and HRMS, as detailed in the Supplementary Materials.

### 2.2. Fluorescent Responses of the above Probes to I-Motif Structures

**Figure 2:**
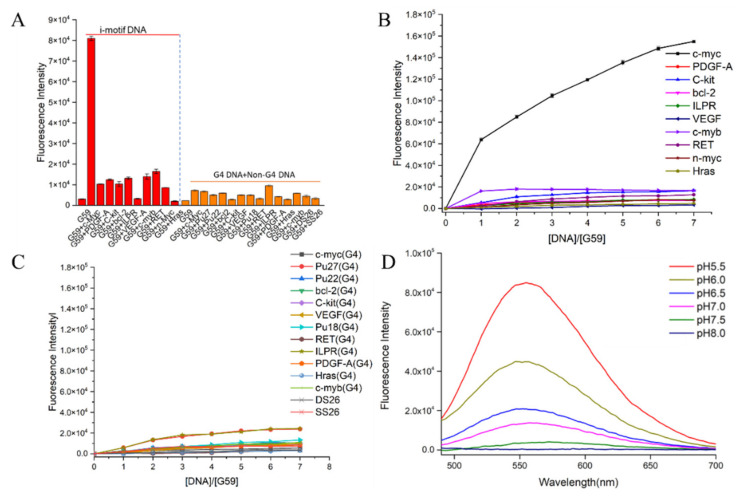
Fluorescent property of **G59** upon addition of various DNAs with λex of 407 nm. (**A**) Fluorescent intensity of 1 μM **G59** with 2 μM different i-motifs in 1 × BPES buffer at pH 5.5, and 1 μM **G59** with 2 μM different G-quadruplexes and linear DNAs in 20 mM Tris–HCl buffer containing 100 mM KCl at pH 7.4. (**B**) Fluorescence titration spectra of **G59** with various i-motifs in 1 × BPES buffer at pH 5.5. (**C**) Fluorescence titration spectra of **G59** with various G-quadruplexes and linear DNAs in 20 mM Tris–HCl buffer containing 100 mM KCl at pH 7.4. (**D**) Fluorescence spectra of 1 μM **G59** with 2 μM *c-myc* promoter i-motif at different pH.

The fluorescent responses of the probes towards the *c-myc* promoter i-motif were investigated. As shown in Supplementary Figure S1A, our data showed strong turn-on fluorescence enhancement upon incubation with the *c-myc* i-motif, especially for compound **G59**, with two conjugated naphthalimide structures. In comparison to **G49**, **G50**, **G51**, and **G60–G67**, compound **G59** exhibited unprecedented strong fluorescence enhancement, with a low background signal, which was possibly due to its high binding affinity with the i-motif. Then, in order to investigate the specificity of **G59** to the *c-myc* promoter i-motif, we measured its response to various other DNA structures for comparison, including promoter i-motifs, G-quadruplexes, double-strand DNA (dsDNA), and single-strand DNA (ssDNA). As shown in Figure 2A and Figure S1B,C, **G59** had a significant fluorescent increase upon addition of the *c-myc* promoter i-motif, with a relatively weak response to other DNA structures, which indicated that **G59** had certain specificity to the *c-myc* promoter i-motif, as a novel specific light-up system. Additionally, **G59** itself had no significant fluorescent response under various pH conditions, as shown in Figure S1D, making fluorescent sensing under various pH conditions possible [15]. In order to confirm the specificity of **G59** to the *c-myc* promoter i-motif, a fluorescence titration experiment was carried out with various i-motifs, G-quadruplexes, double-strand (DS26) DNA, and single-strand (SS26) DNA. As shown in Figure 2B,C, the fluorescence intensity of **G59** was significantly enhanced upon incubation with the *c-myc* promoter i-motif at increasing concentrations, in comparison with the weak fluorescence enhancement for other DNAs. To the best of our knowledge, this is the first example of a fluorescent probe with selective recognition for a *c-myc* promoter i-motif.

Subsequently, because i-motif formation is sensitive to pH [26], a further fluorescence experiment was carried out to incubate **G59** with the *c-myc* promoter i-motif under the pH range 5.5–8.0, which showed maximal fluorescence enhancement at pH 5.5 (Figure 2D). Since i-motif structures are generally more stable under acidic conditions (Figure S2), our result indicated that **G59** could selectively interact with the i-motif structure, instead of linear C-rich DNA, which was consistent with our previously reported results for other i-motif-binding molecules [20,25,27].

### 2.3. The Sensitivity of **G59** to C-Myc Promoter I-Motif

**Figure 3:**
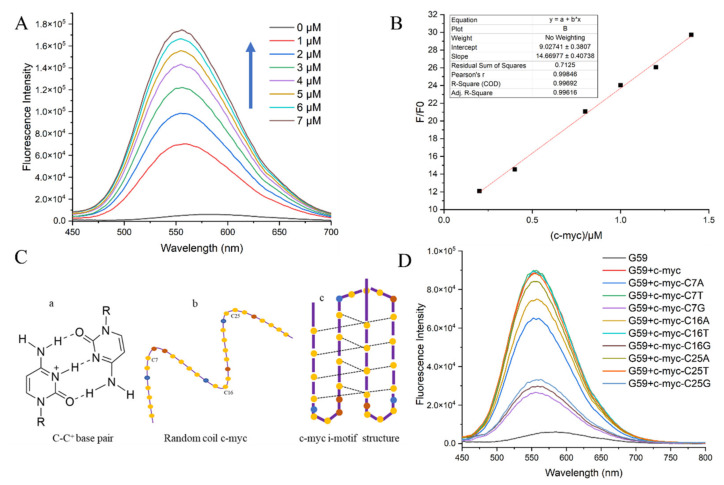
Fluorescent specificity of **G59** upon addition of *c-myc* promoter i-motif in 1 × BPES buffer at pH 5.5 with λex of 407 nm. (**A**) Fluorescence titration of 1 μM **G59** through stepwise addition of *c-myc* promoter i-motif (arrows indicate 0–7 μM). (**B**) A linear relationship was obtained for fluorescence intensity against increasing concentrations of *c-myc* promoter i-motif. (**C**) Schematic illustration of the i-motif structure: (a) cytosine-protonated cytosine base pair; (b) random coil *c-myc* promoter; (c) *c-myc* promoter i-motif structure, with yellow dots representing cytosine, blue dots representing adenine, and brown dots representing thymine. (**D**) Fluorescence spectra of *c-myc* promoter i-motif wild-type and mutants.

The detection limit is a major criterion for evaluating an ideal sensor for biosensing [28,29]. Here, the sensitivity of probe **G59**, in terms of the detection limit, was measured through a fluorescence titration experiment, with the *c-myc* promoter i-motif as a model substrate. The fluorescence spectra for the titration of **G59** with increasing concentrations of *c-myc* promoter i-motif are shown in Figure 3A. With the addition of increasing concentrations of *c-myc* promoter i-motif, the fluorescence intensity of **G59** increased accordingly, with a good linear relationship in the concentration range 0.2–1.4 μM of i-motif against 1 μM **G59**, as shown in Figure 3B. **G59** exhibited the lowest detection limit of 0.154 μM, indicating its high sensitivity towards the *c-myc* promoter i-motif. 

In order to further investigate the sensitivity of **G59** to a wild-type *c-myc* promoter i-motif, we performed a fluorescence experiment to compare the wild-type *c-myc* promoter i-motif with mutants in loop regions. The *c-myc* promoter i-motif is a four-stranded antiparallel structure, formed through intercalated hemiprotonated cytosine–cytosine (C-C^+^) base pairs with three loops, as shown in Figure 3C. We mutated one base on each loop, including C7 on loop 1, C16 on loop 2, and C25 on loop 3. Our circular dichroism (CD) experiment indicated that these mutations did not have a significant effect on the formation of the i-motif structure, as shown in Figure S3. In comparison, our fluorescence emission experiment showed that **G59** had significantly decreased binding to some of these mutants, as shown in Figure 3D and Table S2. Different mutations on the loops had different effects on fluorescence intensity, indicating that **G59** could have significant interactions with these loops, possibly through hydrogen bonding or electrostatic interactions [30,31]. These results showed that some minor mutations could significantly affect the fluorescence signal of the wild-type **G59**/*c-myc* promoter i-motif binding complex, suggesting that **G59** could be applied for diagnosing relevant diseases caused by mutations. 

### 2.4. Binding Mechanism of **G59** with C-Myc Promoter I-Motif and Fluorescent Visualization Experiments

**Figure 4:**
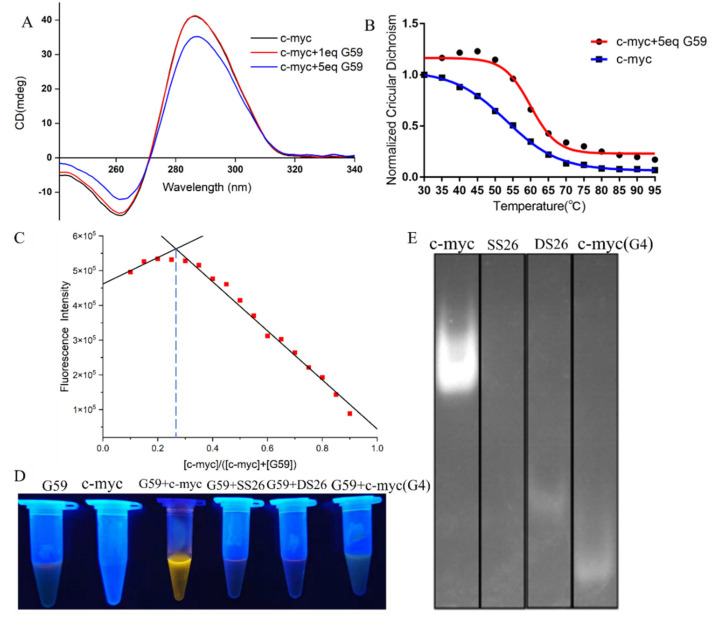
Binding studies of **G59** with *c-myc* promoter i-motif. (**A**) CD spectra of 2 μM *c-myc* promoter i-motif without and with 1 eq or 5 eq (2 μM or 10 μM) **G59** in 1 × BPES buffer at pH 5.5. (**B**) CD melting curves of 2 μM *c-myc* promoter i-motif without and with 5 eq (10 μM) **G59**. (**C**) The Job’s plot curve of **G59** probe with *c-myc* promoter i-motif. (**D**) Fluorescence change in **G59** with *c-myc* promoter i-motif and other DNAs. (**E**) Gel electrophoresis of *c-myc* promoter i-motif, single-strand DNA (SS26), double-strand DNA (DS26), and *c-myc* promoter G-quadruplex, followed by incubation using **G59** probe as a staining agent.

In order to know the possible interactions between **G59** and the *c-myc* promoter i-motif, we carried out a CD titration experiment. As shown in Figure 4A, the addition of **G59** did not have much of an effect on its characteristic CD peaks, indicating that their interactions did not induce significant conformational changes on the i-motif [15,32,33]. On the other hand, a CD melting experiment was carried out to study whether **G59** could affect the stability of the *c-myc* promoter i-motif. As shown in Figure 4B, the melting temperature of the *c-myc* promoter i-motif alone was determined to be 53.1 °C. After the addition of **G59**, the melting temperature increased to 59.9 °C, with the Δ*T_m_* value determined to be 6.8 °C, indicating its good stabilization to the *c-myc* promoter i-motif [15,16,34]. An isothermal titration calorimetry (ITC) experiment was also carried out to study their thermodynamic binding property [35], with their binding isotherms determined as shown in Figure S4. The Gibbs free energy (ΔG°) was found to be negative, indicating that their binding was a spontaneous process. Our data showed that their interactions were exothermic, with the thermodynamic parameters listed in Table S3. These data revealed that **G59** could bind to, and thermally stabilize, the i-motif, possibly through hydrogen bonding, electrostatic interactions, and van der Waals forces [36]. The stoichiometry between **G59** and the *c-myc* promoter i-motif was studied using Job’s plot method [37]. As shown in Figure 4C, the plot gave an intersection point at around 0.3, indicating that **G59** bound to the *c-myc* promoter i-motif at a stoichiometry of 1:2. This result showed that one molecule of **G59** could bind to two molecules of *c-myc* promoter i-motif, which was consistent with our ITC result. Our above results were similar to those of other fluorescent probes for DNA secondary structures [38].

Then, we explored the potential applications of **G59** for the visualization of nucleic acids. After the addition of the **G59** probe to various types of DNA solutions, the *c-myc* promoter i-motif showed bright yellow fluorescence under UV light, as shown in Figure 4D. In comparison, other types of DNAs showed no significant fluorescence change, indicating the good selectivity of the **G59** probe to the *c-myc* promoter i-motif, which was consistent with our above experimental data. Next, we explored the potential application of **G59** as a selective staining agent. Various types of DNAs, including the *c-myc* promoter i-motif, single-strand DNA, double-strand DNA, and the *c-myc* promoter G-quadruplex, were analyzed by using polyacrylamide gel electrophoresis (PAGE). As shown in Figure 4E, upon incubation with **G59**, as a staining agent, the *c-myc* promoter i-motif was selectively visualized under UV light illumination. As we know, **G59** is the first example of a probe with a good “light-switch” effect on the *c-myc* promoter i-motif under UV light illumination. 

### 2.5. Application of **G59** in Screening for Potential C-Myc I-Motif Binding Ligands

**Figure 5:**
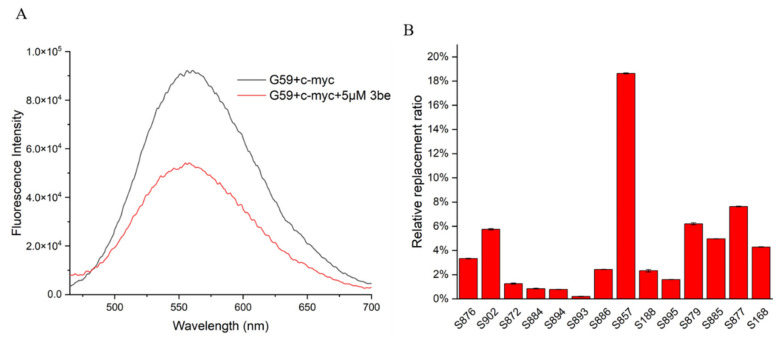
Application of **G59** for screening *c-myc* i-motif binding ligands. (**A**) Fluorescent spectra for mixture of 1 μM **G59** and 2 μM *c-myc* i-motif, with or without addition of 5 eq (5 μM) **3be** in 1 × BPES buffer at pH 5.5. (**B**) Relative **G59** replacement ratio was used for screening natural products for potential *c-myc* i-motif binding ligands.

**Figure 6:**
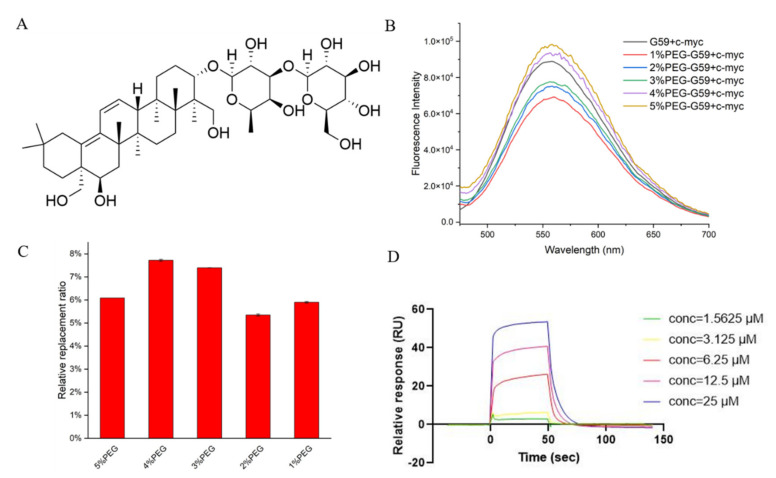
Studies for **G59** replacement by natural product **S857** upon binding with *c-myc* promoter i-motif. (**A**) Chemical structure of **S857**. (**B**) Fluorescent spectra for mixture of 1 μM **G59** and 2 μM *c-myc* i-motif in 1 × BPES buffer at pH 5.5, or 1 × BPES buffer at pH 6.5, with an increasing amount of PEG as the crowding agent. (**C**) Relative **G59** replacement ratio for natural product **S857** in 1 × BPES buffer at pH 6.5, with an increasing amount of PEG as the crowding agent. (**D**) The binding affinity of **S857** to *c-myc* i-motif was analyzed using SPR in MES buffer.

So far, a limited number of i-motif binding ligands have been reported, including **IMC-48**, **IMC-76**, **a9**, **B19**, **A22,** and **WZZ-02** [20,21,23,25,30]. In order to discover and develop more i-motif binding ligands rapidly and economically, efficient and accurate screening methods are required. The fluorescent intercalator displacement (FID) assay is a high-throughput method that is useful for ligand discovery, which relies on a non-covalent intercalator that fluoresces when bound to DNA, but not when competitively displaced by a binding ligand. In the present study, we developed a ligand screening method for *c-myc* i-motif binding molecules, by using an FID assay. **G59** was used as a selective probe for the *c-myc* i-motif, with excellent sensitivity, with their fluorescence changed upon incubation with various ligands, analyzed by using a multifunctional microplate reader. As shown in Figure 5A, with the addition of 5 eq **3be** as a control, its fluorescence intensity decreased, with its relative replacement ratio determined to be 42.09%, by using a fluorescence spectrometer. Similar replacement ratio data were obtained when a multifunctional microplate reader was used for detection at λem of 553 nm (Table S4). Subsequently, a series of natural products were screened by using a multifunctional microplate reader, and **S857** (Saikosaponin B2, Figure 6A) was found to have a relatively good replacement ratio (Figure 5B and Table S4), indicating its possible binding with the *c-myc* i-motif.

It should be mentioned that potential *c-myc* i-motif binding ligands are normally screened under acidic conditions, by using instrumental methods, such as surface plasmon resonance (SPR), because the i-motif is unstable under neutral conditions. These discovered binding ligands have been questioned in further cellular studies, because different pH conditions might affect their possible binding in cells. As mentioned before, the i-motif could be stabilized under neutral crowding conditions; however, sticky crowding agents could not be used in the instrumental analysis. In order to explore the possibility of screening i-motif binding ligands under near-neutral pH for in-depth cellular and animal studies, the fluorescence spectra for the mixture of **G59** and the *c-myc* i-motif were recorded in different molecular crowding conditions, at pH 6.5. We found that their fluorescence intensity increased at pH 6.5, as the concentration of the polyethylene glycol (PEG) crowding agent increased (Figure 6B), indicating that PEG could stabilize the i-motif structure to enable a stronger interaction with **G59**. The relative **G59** replacement ratio data for **S857**, measured in crowding conditions, became slightly lower (Figure 6C), indicating that the i-motif might have strong selective binding with **G59** under neutral crowding conditions. Then, an SPR experiment was performed, as shown in Figure 6D and Figure S5, and the *K*_D_ values for the binding of **S857** with the i-motif and G-quadruplex were determined to be 9.52 μM and 46.2 μM, respectively, indicating that **S857** could become a selective *c-myc* i-motif binding ligand for further development. Here, we developed a rapid and sensitive biosensor screening protocol that enables label-free screening for selective *c-myc* i-motif binding ligands. This screening method can avoid the effect of traditional SPR labeling on the i-motif structure, and can be performed under neutral crowding conditions for consistent further cellular and animal studies. The method could be applied for the high-throughput screening of small molecule compound libraries, with minimum time and expense.

## 3. Discussion

The i-motif has been recognized as an important molecular target, and studies have been focused on the development of i-motif binding ligands, due to its biological function in gene regulation [4]. In recent years, progress has been made in developing fluorescent probes for i-motif structures. However, their application is limited, due to reasons such as low specificity or selectivity, weak fluorescence signal or sensitivity, and expensive conjugated labeling requirements [17,26]. In some cases, two or more fluorescent probes are required to differentiate i-motif structures from other nucleic acids [6,39]. A selective fluorescent i-motif probe is urgently required, in order to clarify the biological functions of the i-motifs in complex biological systems, which is one of the major challenges in this field. In this study, **G59** was found to be a specific i-motif binding probe, with a strong and selective fluorescence performance, which could become a label-free fluorescence sensing system for direct and fast detection and verification of the i-motif structure. It could also offer a sensitive and accurate method for drug screening, based on an FID assay, because of its high sensitivity. It is an economic and efficient method to find potential i-motif binding ligands for studying the functions of i-motifs in biological systems, for purposes of gene expression analysis and disease diagnosis.

An antibody fragment (iMab) has been found to recognize i-motif structures, enabling the detection of i-motifs in the nuclei of human cells [6]. iMab is a broad-spectrum macromolecule that can simultaneously detect multiple i-motif structures; however, it is not commercially available. Our present study could provide a supplemental method for detecting i-motif structures with a small molecule. **G59** showed high affinity and a strong fluorescence response to the *c-myc* promoter i-motif, without a significant response to other i-motif structures, indicating its possible selective detection of only certain related diseases.

## 4. Material and Methods

### 4.1. Materials and Characterization

All chemicals and starting materials were purchased from commercial sources, which were analytical grade without further purification unless otherwise specified. ^1^H and ^13^C NMR spectra were recorded on a Bruker BioSpin GmbH spectrometer (Bruker, Switzerland). HRMS were recorded on a Shimadzu LCMS-IT-TOF of MAT95XP mass spectrometer (Thermo Fisher Scientific, Waltham, MA, USA).

### 4.2. Syntheses of Fluorescent Probes

Syntheses were carried out as shown in Supporting Information in Scheme S1A–D.

### 4.3. DNA Oligonucleotides

DNA oligonucleotides were purchased from Sangon (China) as salt-free oligomers, which were then dissolved in relevant buffers, with their sequences as shown in Table S1.

### 4.4. The Limit of Detection (LOD)

The limit of detection (LOD) of **G59** was obtained through fluorescence titration and estimated based on the following calculation formula: LOD = K (Sb/m). In the equation, Sb is the standard deviation of the blank multiple measurements (*n* = 20), and m is the slope of the calibration curve, which represents the sensitivity of this method. According to the International Union of Pure and Applied Chemistry (IUPAC), the K value is generally taken to be 3.

### 4.5. CD Experiments

Circular dichroism (CD) studies were performed on a Chirascan circular dichroism spectrophotometer (Applied Photophysics, Leatherhead, UK). A quartz cuvette with 10 mm path length was used for the spectra recorded over a wavelength range of 230–350 nm at 1 nm bandwidth, 1 nm step size, and 0.5 s per point. CD melting was performed at a fixed concentration of nucleic acid (2 μM), either with or without a fixed concentration (10 μM) of **G59** in 1 × BPES buffer (30 mM KH_2_PO_4_, 30 mM K_2_HPO_4_, 1 mM EDTA, and 100 mM KCl) at pH 5.5. The data were recorded at intervals of 5 °C, over a range of 30–95 °C, with a heating rate of 2.5 °C/min. 

### 4.6. Job’s Plot

To gain a better understanding of the stoichiometry between **G59** and *c-myc* promoter i-motif, independent fluorescence spectra were obtained using various concentrations of **G59** and *c-myc* promoter i-motif, while the sum concentrations of **G59** and *c-myc* promoter i-motif remained as 10 μM. 

### 4.7. Isothermal Titration Calorimetry (ITC)

The thermodynamic parameters for the binding interactions of **G59** with *c-myc* promoter i-motif were determined using an isothermal titration calorimeter (VP-ITC, Microcal, Northampton, MA, USA). The calorimeter contains a pair of sample and reference cells, which are packed in an adiabatic chamber. The sample cell had *c-myc* promoter i-motif DNA and the reference cell had a buffer. A syringe with a volume of 280 μL, containing **G59** solution, was used for injection into the sample cell with a volume of 1.4235 mL. Each experiment had 28 consecutive injections of 10 μL of 100 μM **G59** to 4 μM *c-myc* promoter i-motif DNA in the sample cell for a duration of 20 s with a 180 s interval between the consecutive injections. 

### 4.8. PAGE Experiment

Different oligonucleotides were loaded onto a 20% bisacrylamide gel in 1 × TBE buffer (pH 5.5) and electrophoresed at 4 °C at 140 V for 5 h. The i-motif was diluted to the required concentration (0.1 mM) in BPES buffer. The oligonucleotides were stained with **G59** (0.5 mM), and DNA bands were visualized under UV light and photographed using AlphaImager EC. 

### 4.9. SPR Experiment

The SPR measurement was performed on a ProteOn XPR36 Protein Interaction Array system (Bio-Rad Laboratories, Hercules, CA, USA), using a Neutravidin-coated GLH sensor chip. For immobilization, all DNA samples were biotinylated and attached to a reptavidin-coated sensor chip. The 5′-biotin-labeled *c-myc* i-motif was diluted to 1 μM in MES running buffer (20 mM 2-(4-morpholino) ethanesulfonic acid, pH 5.5, 100 mM KCl and 0.05% Tween-20), and the 5′-biotin-labeled *c-myc* G-quadruplex was diluted to 1 μM in running buffer (Tris-HCl 20 mM, pH 7.4, 100 mM KCl). The DNA samples were then captured (1000 RU) in flow cells, and a blank cell was set as the control. **S857** was prepared with the running buffer through serial dilutions from a stock solution (10 mM in DMSO). **S857** of different concentrations were injected simultaneously at a flow rate of 25 mL/min for 50 s in the association phase, followed by 90 s in the dissociation phase at 25 °C. The GLH sensor chip was regenerated with a short injection of 50 mM NaOH between consecutive measurements. The final graphs were obtained by subtracting blank sensorgrams from the i-motif and G-quadruplex sensorgrams. Data were analyzed with ProteOn manager software.

## 5. Conclusions

In summary, after the syntheses and evaluation of some carbazole derivatives, we developed a fluorescent probe **G59** with selective binding to the *c-myc* gene promoter i-motif, with excellent binding affinity. **G59** showed significant fluorescence enhancement upon binding with the i-motif, with little response to other DNA structures. **G59** exhibited strong stabilization to the *c-myc* i-motif and a low fluorescent detection limit (154 nM), with a large Stokes shift, which is valuable for i-motif visualization in solution. **G59** could be applied in screening for selective *c-myc* i-motif binding ligands under neutral crowding conditions, as a rapid and sensitive biosensor for label-free screening. To the best of our knowledge, **G59** is the first fluorescent probe with high sensitivity for recognizing i-motif structures, and is an economic tool to screen for selective *c-myc* i-motif binding ligands.

## Data Availability

The data presented in this study are available in both article and Supplementary Materials.

## Supplementary Materials

The following supporting information can be downloaded at: https://www.mdpi.com/article/10.3390/ijms23073872/s1.
