# Supplementary material for: Development of a Smart Fluorescent Probe Specifically Interacting with C-Myc I-Motif
Source: Int J Mol Sci. 2022 Mar 31;23(7):3872. doi: 10.3390/ijms23073872 (PMC8998492; doi:10.3390/ijms23073872)

Supporting Information for

Development of a smart fluorescent probe specifically interacting with c-myc i-motif

Zuzhuang Wei, Bobo Liu, Xiaomin Lin, Jing Wang, Zhi-Shu Huang, Ding Li*

School of Pharmaceutical Sciences, Sun Yat-sen University, Guangzhou University City, 132 Waihuan East Road, Guangzhou 510006, P. R. China

weizzh@mail2.sysu.edu.cn (Z.W.); liubb9@mail2.sysu.edu.cn (B.L.); linxm35@mail2.sysu.edu.cn (X.L.); wangj349@mail2.sysu.edu.cn (J.W.); ceshzs@mail.sysu.edu.cn (Z.-S.H.)

Correspondence: liding@mail.sysu.edu.cn; Tel.: +86-20-3994-3058 (D.L.)

1. Syntheses

1.1. Carbazole derivative **G49** was prepared by following Scheme S1A

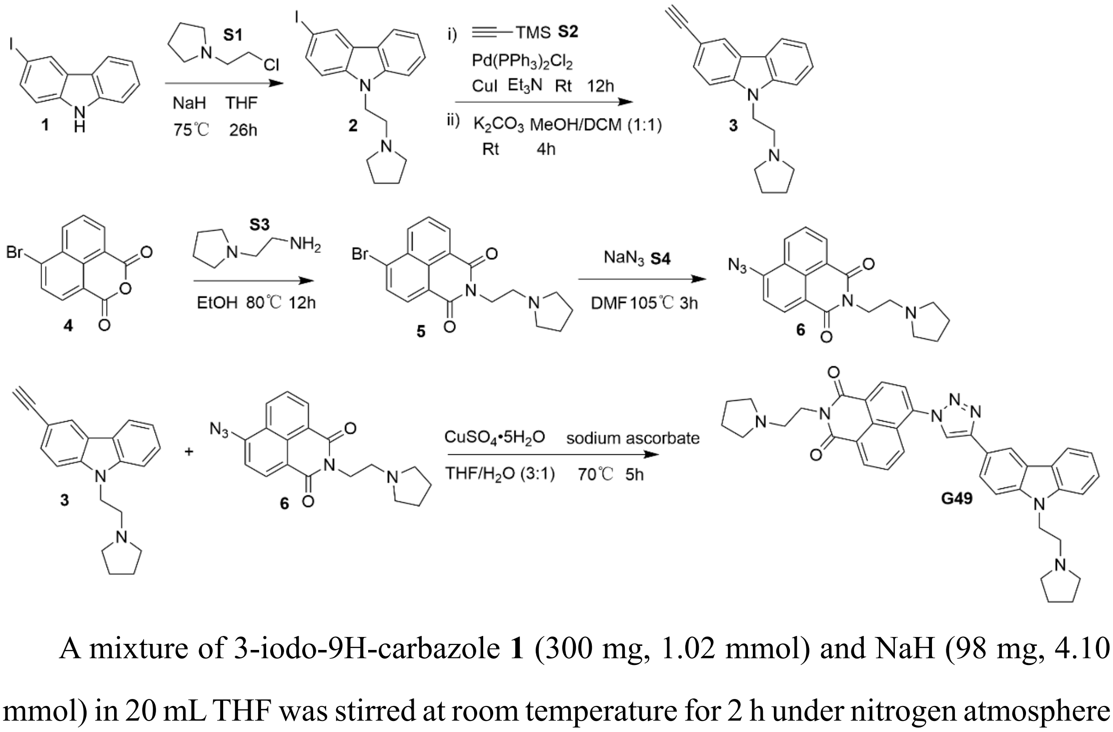

A mixture of 3-iodo-9H-carbazole **1** (300 mg, 1.02 mmol) and NaH (98 mg, 4.10 mmol) in 20 mL THF was stirred at room temperature for 2 h under nitrogen atmosphere. In another flask, 1-(2-chloroethyl) pyrrolidine hydrochloride **S1** (522 mg, 3.07 mmol)

and NaOH (1.5 g) were diluted in 5 mL water and cooled to room temperature, and then the upper layer of this solution was added drop-wise to the above mixture. The reaction mixture was heated at 75 °C for additional 24 h. After removal of the solvent under reduced pressure, the residue was extracted with dichloromethane (3×20 mL). The combined organic layer was washed with brine for three times, and dried over anhydrous sodium sulfate. After removal of the solvent under reduced pressure, the residue was purified by using column chromatography on silica gel with MeOH/CH_2Cl_2 (1:50) to give compound **2** as a yellow liquid (319 mg) with a yield of 80%. ^{1}H NMR (400 MHz, $CDCl_3$) δ 8.38 (d, J = 1.5 Hz, 1H), 8.02 (d, J = 7.8 Hz, 1H), 7.70 (dd, J = 8.6, 1.7 Hz, 1H), 7.50 – 7.41 (m, 2H), 7.23 (d, J = 8.4 Hz, 2H), 4.49 – 4.39 (m, 2H), 2.92 – 2.83 (m, 2H), 2.64 (s, 4H), 1.82 (dd, J = 6.7, 3.3 Hz, 4H). ^{13}C NMR (125 MHz, $CDCl_3$) δ 140.26, 139.45, 133.95, 129.25, 126.49, 125.47, 121.70, 120.58, 119.55, 110.68, 108.77, 81.46, 54.51, 53.96, 42.40, 23.56. ESI-MS (m/z) 391.15 $[M+H]^+$.

To a solution of **2** (200 mg, 0.51 mmol) in Et_3N (6 mL) and Pd $(PPh_3)_2Cl_2$ (36 mg, 0.05 mmol), CuI (10 mg, 0.05 mmol) was added at room temperature. The mixture was stirred for 30 minutes, and then TMS-acetylene **S2** (0.2 mL, 1.54 mmol) was added dropwise. The resulting mixture was stirred at room temperature under nitrogen atmosphere for 12 h. The reaction mixture was quenched with water, and extracted with dichloromethane (3×20 mL). The combined extracting solvents were concentrated, washed with brine, and dried over anhydrous sodium sulfate. The crude product was purified by using column chromatography, which was then stirred with 5 equivalents of K_2CO_3 in MeOH-CH_2Cl_2 solution at room temperature under nitrogen atmosphere for 4 h. The solution was washed with brine, dried over anhydrous sodium sulfate, and concentrated. The crude product was purified by using column chromatography to give **3** as a brown oil (121 mg, 82%). ^{1}H NMR (400 MHz, $CDCl_3$) δ 8.09 (d, J = 1.2 Hz, 1H), 7.90 (d, J = 7.8 Hz, 1H), 7.44 (dd, J = 8.5, 1.6 Hz, 1H), 7.34 – 7.30 (m, 1H), 7.28 (d, J = 3.0 Hz, 1H), 7.20 (d, J = 8.4 Hz, 1H), 7.12 – 7.07 (m, 1H), 4.31 – 4.21 (m, 2H), 2.93 (s, 1H), 2.75 – 2.66 (m, 2H), 2.49 – 2.44 (m, 4H), 1.68 – 1.64 (m, 4H). ^{13}C NMR (100

MHz, $CDCl_3$) δ 140.70, 140.20, 129.73, 128.60, 128.48, 126.31, 124.70, 122.84, 122.44, 120.57, 119.60, 112.21, 108.84, 108.59, 85.07, 75.23, 54.54, 54.08, 42.59, 23.59. ESI-MS (m/z) 289.20 $[M+H]^+$.

A mixture of 4-bromo-1,8-naphthalic anhydride **4** (500 mg, 1.81 mmol) and 1-(2-aminoethyl) pyrrolidine **S3** (275 mg, 2.41 mmol) in 30 mL absolute ethanol was heated at 80 °C for 12 h under nitrogen atmosphere, and monitored by using TLC. After cooling down, the solvent was removed under reduced pressure, and the residue was extracted with dichloromethane (3×20 mL). The combined organic layer was washed with brine for three times, dried over anhydrous sodium sulfate, and concentrated. The residue was purified by using column chromatography on silica gel with $MeOH/CH_2Cl_2/NH_4OH$ (1:60:0.01) to give compound **5** as a yellow solid (577 mg) with a yield of 86%. ^1H NMR (400 MHz, $CDCl_3$) δ 8.59 – 8.55 (m, 1H), 8.49 – 8.44 (m, 1H), 8.33 (d, J = 7.9 Hz, 1H), 7.96 (d, J = 7.9 Hz, 1H), 7.78 (dd, J = 8.3, 7.5 Hz, 1H), 4.38 – 4.27 (m, 2H), 2.84 – 2.75 (m, 2H), 2.67 – 2.60 (m, 4H), 1.79 – 1.72 (m, 4H). ^{13}C NMR (100 MHz, $CDCl_3$) δ 163.51, 163.48, 133.15, 131.98, 131.16, 131.03, 130.50, 130.18, 128.91, 128.02, 123.04, 122.18, 54.38, 53.59, 39.35, 23.61. ESI-MS (m/z) 373.10 $[M+H]^+$.

A mixture of **5** (200 mg, 1.81 mmol) and sodium azide **S4** (275 mg, 2.41 mmol) in 10 mL anhydrous dimethylformamide was heated at 105 °C for 3 h under nitrogen atmosphere. The reaction mixture was quenched with water, and extracted with dichloromethane (3×20 mL). The combined organic layer was washed with brine for three times, dried over anhydrous sodium sulfate, concentrated under reduced pressure to give crude compound **6**, which was used for the next step without further purification.

Compound **3** (200 mg, 0.60 mmol) was dissolved in a 3:1 mixture of THF/H_2O (8 mL). Copper (II) sulphate pentahydrate (15 mg, 0.06 mmol) and sodium ascorbate (12 mg, 0.06 mmol) were added, and the solution was stirred for 10 min. The corresponding azide **6** (258 mg, 0.90 mmol) was added, and the mixture was heated for 4 h at 70 °C

under nitrogen atmosphere, and then quenched with water. Dichloromethane (50 mL) was added, and the mixture was stirred for 10 minutes. After separation and extraction with dichloromethane (3×20 mL), the combined organic layer was washed with brine for three times, and dried over anhydrous sodium sulfate. After removal of the solvent under reduced pressure, the residue was purified by using column chromatography on silica gel with MeOH/CH_2Cl_2/NH_4OH (1:50:0.01) to give compound **G49** as a yellow solid (577 mg) with a yield of 81%. ^{1}H NMR (400 MHz, $CDCl_3$) δ 8.70 – 8.61 (m, 2H), 8.41 (dd, J = 8.5, 0.9 Hz, 1H), 8.23 (s, 1H), 8.12 (d, J = 7.7 Hz, 1H), 7.81 (d, J = 7.5 Hz, 1H), 7.73 – 7.66 (m, 1H), 7.61 (d, J = 0.9 Hz, 2H), 7.54 (d, J = 5.1 Hz, 2H), 7.28 (s, 1H), 4.61 – 4.52 (m, 2H), 4.47 – 4.36 (m, 2H), 3.03 – 2.96 (m, 2H), 2.93 – 2.83 (m, 2H), 2.74 – 2.66 (m, 8H), 1.90 – 1.81 (m, 8H). ^{13}C NMR (100 MHz, $CDCl_3$) δ 164.42, 164.23, 148.03, 140.85, 140.24, 133.23, 131.12, 130.90, 130.51, 129.58, 128.89, 128.26, 127.74, 126.66, 126.40, 123.24, 122.84, 122.68, 121.97, 121.11, 120.58, 119.54, 108.94, 108.71, 54.63, 54.40, 54.22, 53.68, 42.71, 39.20, 23.65, 23.61. HRMS (ESI) calculated for $C_{38}H_{37}N_7O_2$, $[M+H]^{2+}$, 321.6577; found, 321.6578.

1.2. Carbazole derivative **G50** was prepared by following Scheme S1B

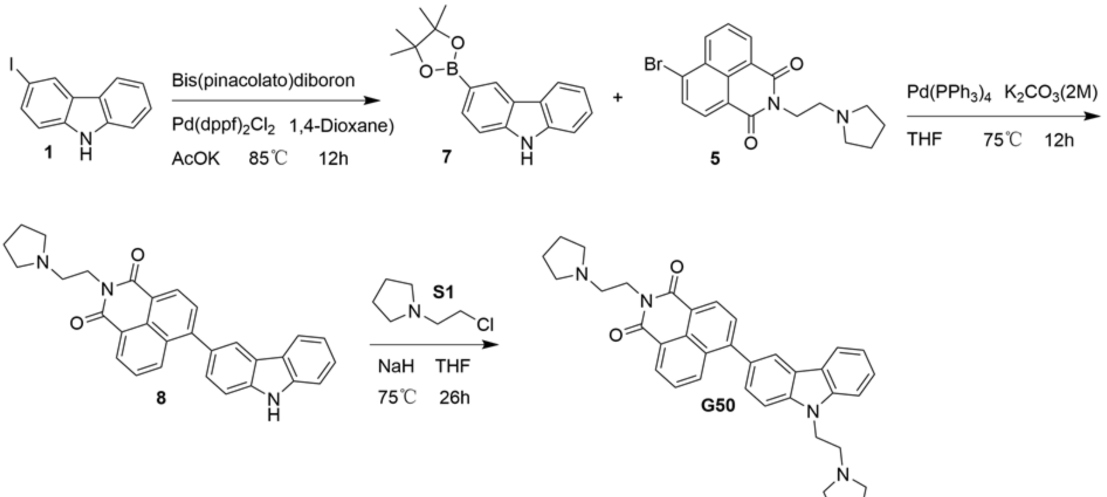

A mixture of 3-iodo-9H-carbazole **1** (500 mg, 1.71 mmol), bis(pinacolato)diboron (650 mg, 2.54 mmol), Pd(dppf)$_2$Cl$_2$ (125 mg, 0.17 mmol), and AcOK (502 mg, 5.12 mmol) in 30 mL absolute 1,4-dioxane was stirred at 85 °C for 12 h under nitrogen atmosphere, and monitored by using TLC. After cooling down, the reaction mixture

was quenched with water. After removal of the solvent under reduced pressure, the mixture was extracted with dichloromethane (3×20 mL). The combined organic layer was washed with brine for three times, dried over anhydrous sodium sulfate, and concentrated. The residue was purified by using column chromatography on silica gel with ethyl acetate/ petroleum ether (1:4) to give compound **7** as a white solid (450 mg) with a yield of 90%. ^{1}H NMR (400 MHz, $CDCl_3$) δ 8.59 (s, 1H), 8.14 – 8.06 (m, 2H), 7.90 – 7.84 (m, 1H), 7.41 – 7.33 (m, 3H), 7.23 (ddd, J = 8.0, 6.0, 2.1 Hz, 1H), 1.40 (s, 12H). ^{13}C NMR (100 MHz, $CDCl_3$) δ 141.71, 139.44, 132.35, 127.78, 125.89, 123.47, 123.08, 120.57, 119.84, 110.62, 83.68, 24.97. ESI-MS (m/z) 294.20 $[M+H]^+$.

A mixture of **7** (200 mg, 0.68 mmol), 2M K_2CO_3 (3 mL), $Pd(PPh_3)_4$ (79 mg, 0.07 mmol) and **5** (381 mg, 1.02 mmol) in 20 mL absolute tetrahydrofuran was stirred at 75 °C for 12 h under nitrogen atmosphere, and monitored by using TLC. The reaction mixture was quenched with water, and the solvent was removed under reduced pressure, and the resulting solution was extracted with dichloromethane (3×20 mL). The combined organic layer was washed with brine for three times, dried over anhydrous sodium sulfate, and concentrated under reduced pressure. The residue was purified by using column chromatography on silica gel with $MeOH/CH_2Cl_2/NH_4OH$ (1:60:0.01) to give compound **8** as a yellow solid (266 mg) with a yield of 85%. ^{1}H NMR (400 MHz, $CDCl_3$) δ 10.34 (s, 1H), 8.30 (d, J = 7.6 Hz, 1H), 8.15 (d, J = 8.4 Hz, 1H), 8.08 (d, J = 7.1 Hz, 1H), 7.82 – 7.71 (m, 2H), 7.44 – 7.27 (m, 6H), 7.13 (t, J = 7.2 Hz, 1H), 4.50 (t, J = 5.9 Hz, 2H), 3.14 (t, J = 5.8 Hz, 2H), 2.93 (s, 4H), 1.91 (s, 4H). ^{13}C NMR (100 MHz, $CDCl_3$) δ 164.47, 164.40, 147.23, 140.05, 139.45, 132.61, 130.31, 130.17, 129.24, 128.96, 128.37 127.56, 126.91, 126.19, 125.96, 122.81, 122.56, 121.99, 121.89, 119.91, 119.68, 119.30, 111.06, 110.81, 54.50, 54.22, 38.93, 23.65. ESI-MS (m/z) 460.20 $[M+H]^+$.

A mixture of **8** (200 mg, 0.44 mmol) and NaH (42 mg, 1.74 mmol) in 20 mL THF was stirred at room temperature for 2 h under nitrogen atmosphere. In another flask, a

mixture of 1-(2-chloroethyl) pyrrolidine hydrochloride **S1** (222 mg, 1.31 mmol) and NaOH (1.5 g) in 5 mL water was prepared at room temperature, with its upper layer added dropwise to the previous solution. The reaction mixture was heated at 75 °C for additional 24 h, and monitored by using TLC, which was then quenched with water. After removal of the solvent under reduced pressure, the solution was extracted with dichloromethane (3×20 mL). The combined organic layer was washed with brine for three times, and dried over anhydrous sodium sulfate. After removal of the solvent under reduced pressure, the residue was purified by using column chromatography on silica gel with MeOH/CH_2Cl_2/NH_4OH (1:60:0.01) to give compound **G50** as a yellow liquid (194 mg) with a yield of 80%. ^1H NMR (400 MHz, $CDCl_3$) δ 8.57 (dd, J = 7.0, 5.4 Hz, 3H), 8.32 (d, J = 8.6 Hz, 1H), 8.23 (s, 1H), 8.02 (d, J = 7.6 Hz, 1H), 7.97 (dd, J = 8.5, 1.5 Hz, 1H), 7.81 – 7.71 (m, 2H), 7.49 – 7.38 (m, 3H), 7.20 (t, J = 7.3 Hz, 1H), 4.43 (dd, J = 10.0, 5.5 Hz, 2H), 4.34 (t, J = 7.2 Hz, 2H), 2.91 – 2.80 (m, 4H), 2.69 – 2.61 (m, 8H), 1.85 – 1.75 (m, 8H). ^{13}C NMR (100 MHz, $CDCl_3$) δ 163.56, 163.03, 149.35, 140.66, 140.43, 138.14, 132.01, 130.55, 129.61, 129.00, 128.36, 126.24, 123.84, 123.57, 123.30, 123.13, 122.81, 122.71, 120.95, 120.55, 119.46, 117.99, 109.03, 108.81, 54.58, 54.42, 54.14, 53.62, 42.64, 39.46, 23.64, 23.59. HRMS (ESI) calculated for $C_{36}H_{36}N_4O_2$ $[M + H]^{2+}$, 279.1492; found, 279.1491.

1.3. Carbazole derivative **G51** was prepared by following Scheme S1C

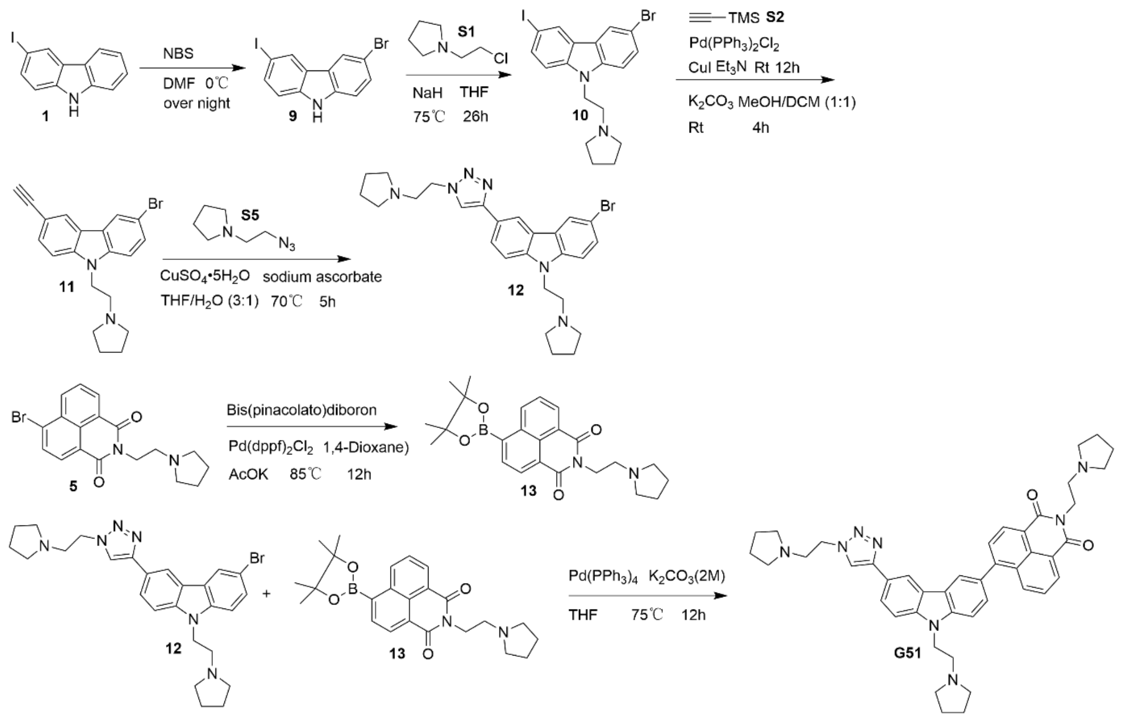

A mixture of 3-iodo-9H-carbazole **1** (200 mg, 0.68 mmol) and NBS (145 mg, 0.82 mmol) in 10 mL DMF was stirred in the dark at 0 °C overnight under nitrogen atmosphere. The reaction mixture was quenched with water, and ethyl acetate (50 mL) was added. After stirring for 10 minutes, the solution was separated and extracted with ethyl acetate (3×20 mL). The combined organic layer was washed with brine for three times, dried over anhydrous sodium sulfate, and the solvent was removed under reduced pressure. The residue was purified by using column chromatography on silica gel with ethyl acetate/petroleum ether (1:5) to give compound **9** as a yellow solid (227 mg) with a yield of 90%. ^{1}H NMR (400 MHz, $CDCl_3$) δ 8.32 (d, J = 1.5 Hz, 1H), 8.11 (d, J = 1.8 Hz, 2H), 7.68 (dd, J = 8.5, 1.7 Hz, 1H), 7.51 (dd, J = 8.6, 1.9 Hz, 1H), 7.29 (d, J = 8.6 Hz, 1H), 7.21 (d, J = 8.5 Hz, 1H). ^{13}C NMR (100 MHz, $CDCl_3$) δ 134.84, 129.42, 129.31, 123.26, 112.78, 112.18. ESI-MS (m/z) 369.85 $[M-H]^-$.

A mixture of 3-bromo-6-iodo-9H-carbazole **9** (200 mg, 0.54 mmol) and NaH (52 mg, 2.16 mmol) in 20 mL THF was stirred at room temperature for 2 h under nitrogen atmosphere. In another flask, a mixture of 1-(2-chloroethyl) pyrrolidine hydrochloride **S1** (275 mg, 1.62 mmol) and NaOH (1.5 g) in 5 mL water was prepared, with its upper

layer added dropwise to the previous solution. The reaction mixture was heated at 75 °C for additional 24 h, and quenched with water. Dichloromethane (50 mL) was added and stirred for 10 minutes. The solution was separated and extracted with dichloromethane (3×20 mL). The combined organic layer was washed with brine for three times, dried over anhydrous sodium sulfate, and the solvent was removed under reduced pressure. The residue was purified by using column chromatography on silica gel with MeOH/CH_2Cl_2 (1:50) to give compound **10** as a yellow solid (222 mg) with a yield of 88%. ^{1}H NMR (400 MHz, $CDCl_3$) δ 8.16 (dd, J = 11.0, 4.6 Hz, 2H), 7.56 (ddd, J = 15.8, 8.6, 1.6 Hz, 2H), 7.31 (dd, J = 14.6, 8.6 Hz, 2H), 4.46 – 4.33 (m, 2H), 2.90 – 2.78 (m, 2H), 2.61 (s, 4H), 1.81 (s, 4H), 0.29 (s, 9H). ^{13}C NMR (100 MHz, $CDCl_3$) δ 139.68, 138.87, 134.61, 129.37, 129.08, 124.26, 123.24, 112.22, 110.87, 110.22, 81.79, 54.56, 54.00, 42.76, 23.59. ESI-MS (m/z) 468.95 $[M+H]^+$.

To a mixture of **10** (200 mg, 0.43 mmol) in anhydrous Et_3N (3 mL), Pd $(PPh_3)_2Cl_2$ (30 mg, 0.04 mmol) and CuI (8 mg, 0.04 mmol) were added at room temperature. The mixture was stirred for 30 minutes, and then TMS-acetylene **S2** (0.17mL, 1.28 mmol) was added dropwise. The resulting mixture was stirred under nitrogen atmosphere for 12 h, and then quenched with water. Dichloromethane (50 mL) was added and stirred for 10 minutes. The solution was separated and extracted with dichloromethane (3×20 mL). The combined organic layer was washed with brine for three times, dried over anhydrous sodium sulfate, and the solvent was removed under reduced pressure. The crude product was purified by using column chromatography, and then stirred with 5 equivalents of K_2CO_3 in MeOH-CH_2Cl_2 solution at room temperature under nitrogen atmosphere for 4 h. The crude product was purified by using column chromatography to give **11** as a brown oil (125 mg) with a yield of 80%. ^{1}H NMR (400 MHz, $CDCl_3$) δ 8.15 (dd, J = 9.3, 1.3 Hz, 2H), 7.60 (dd, J = 8.5, 1.4 Hz, 1H), 7.55 (dd, J = 8.7, 1.8 Hz, 1H), 7.35 (d, J = 8.5 Hz, 1H), 7.30 (d, J = 8.7 Hz, 1H), 4.45 – 4.37 (m, 2H), 3.08 (s, 1H), 2.88 – 2.81 (m, 2H), 2.61 (s, 4H), 1.81 (dt, J = 6.4, 3.1 Hz, 4H). ^{13}C NMR (100 MHz, $CDCl_3$) δ 140.43, 139.37, 130.38, 128.97, 124.84, 124.13, 123.30, 121.79, 112.78,

112.38, 110.33, 108.88, 84.62, 75.54, 54.56, 54.05, 42.82, 23.58. ESI-MS (m/z) 367.10 $[M+H]^+$.

To a solution of **11** (150 mg, 0.41 mmol) in a 3:1 mixture of THF/H_2O (8 mL), copper (II) sulphate pentahydrate (10 mg, 0.04 mmol) and sodium ascorbate (8 mg, 0.04 mmol) were added, and the solution was stirred for 10 minutes. The corresponding azide **S5** (116 mg, 0.82 mmol) was added, and the mixture was then heated for 4 h at 70 °C under nitrogen atmosphere. The reaction mixture was quenched with water, and dichloromethane (50 mL) was added. After stirring for 10 minutes, the solution was separated and extracted with dichloromethane (3×20 mL). The combined organic layer was washed with brine for three times, dried over anhydrous sodium sulfate, and the solvent was removed under reduced pressure. The residue was purified by using column chromatography on silica gel with MeOH/CH_2Cl_2/NH_4OH (1:50:0.01) to give compound **12** as a yellow solid (207 mg) with a yield of 83%. ^1H NMR (400 MHz, $CDCl_3$) δ 8.42 (d, J = 1.3 Hz, 1H), 8.15 (d, J = 1.9 Hz, 1H), 7.94 – 7.89 (m, 2H), 7.49 (dd, J = 8.6, 1.9 Hz, 1H), 7.40 (d, J = 8.5 Hz, 1H), 7.23 (d, J = 2.9 Hz, 1H), 4.51 (t, J = 6.6 Hz, 2H), 4.41 – 4.32 (m, 2H), 3.00 (t, J = 6.6 Hz, 2H), 2.86 – 2.78 (m, 2H), 2.56 (dd, J = 7.7, 2.3 Hz, 8H), 1.78 (dt, J = 6.2, 3.3 Hz, 8H). ^{13}C NMR (100 MHz, $CDCl_3$) δ 148.34, 140.41, 139.34, 128.61, 124.66, 124.60, 123.31, 122.48, 122.21, 119.53, 117.86, 111.99, 110.23, 109.14, 55.68, 54.55, 54.19, 54.06, 49.53, 42.76, 23.65, 23.25. ESI-MS (m/z) 507.20 $[M+H]^+$.

A mixture of **5** (300 mg, 0.81 mmol), bis(pinacolato)diboron (614 mg, 2.42 mmol), $Pd(dppf)_2Cl_2$ (59 mg, 0.08 mmol) and AcOK (237 mg, 2.42 mmol) in 30 mL absolute 1,4-dioxane was heated at 85 °C for 12 h under nitrogen atmosphere, and monitored by using TLC. After cooling down, the reaction was quenched with water. The solvent was removed under reduced pressure, and the solution was extracted with dichloromethane (3×20 mL). The combined organic layer was washed with brine for three times, dried over anhydrous sodium sulfate, filtered and concentrated under reduced pressure to give

crude compound **13**, which was used for the next step without further purification.

A mixture of **12** (160 mg, 0.32 mmol), 2M K_2CO_3 (3 mL), $Pd(PPh_3)_4$ (26 mg 0.02 mmol) and **13** (199 mg, 0.47 mmol) in 20 mL absolute tetrahydrofuran was heated at 85 °C for 12 h under nitrogen atmosphere, and monitored by using TLC. The reaction mixture was quenched with water, and the solvent was removed under reduced pressure. The solution was extracted with dichloromethane (3×20 mL), and the combined organic layer was washed with brine for three times, and dried over anhydrous sodium sulfate. The solvent was removed under reduced pressure, and the residue was purified by using column chromatography on silica gel with $MeOH/CH_2Cl_2/NH_4OH$ (1:50:0.01) to give compound **G51** as a yellow solid (184 mg) with a yield of 81%. ^1H NMR (500 MHz, $CDCl_3$) δ 8.63 (dd, J = 12.0, 7.4 Hz, 2H), 8.59 (s, 1H), 8.40 (d, J = 8.5 Hz, 1H), 8.25 (s, 1H), 7.97 (d, J = 8.5 Hz, 1H), 7.94 (s, 1H), 7.78 (d, J = 7.5 Hz, 1H), 7.68 (t, J = 7.8 Hz, 1H), 7.63 – 7.57 (m, 2H), 7.53 (d, J = 8.5 Hz, 1H), 4.54 (dd, J = 13.4, 6.8 Hz, 4H), 4.43 – 4.38 (m, 2H), 3.04 – 2.95 (m, 4H), 2.88 – 2.82 (m, 2H), 2.69 (d, J = 6.4 Hz, 8H), 2.58 (s, 4H), 1.85 (s, 4H), 1.80 (dd, J = 5.9, 3.2 Hz, 8H). ^{13}C NMR (125 MHz, $CDCl_3$) δ 164.44, 164.24, 148.39, 147.85, 140.70, 140.66, 133.18, 131.14, 130.90, 130.42, 129.82, 128.92, 128.22, 128.02, 124.51, 123.28, 123.08, 122.85, 122.60, 122.26, 121.15, 119.51, 117.92, 109.22, 108.98, 55.68, 54.63, 54.40, 54.26, 54.19, 53.70, 49.56, 42.91, 39.17, 23.63. HRMS (ESI) calculated for $C_{44}H_{48}N_8O_2$, $[M+H]^{2+}$, 361.2023; found, 361.2039.

1.4. Carbazole derivative **G59-G67** was prepared by following Scheme S1D

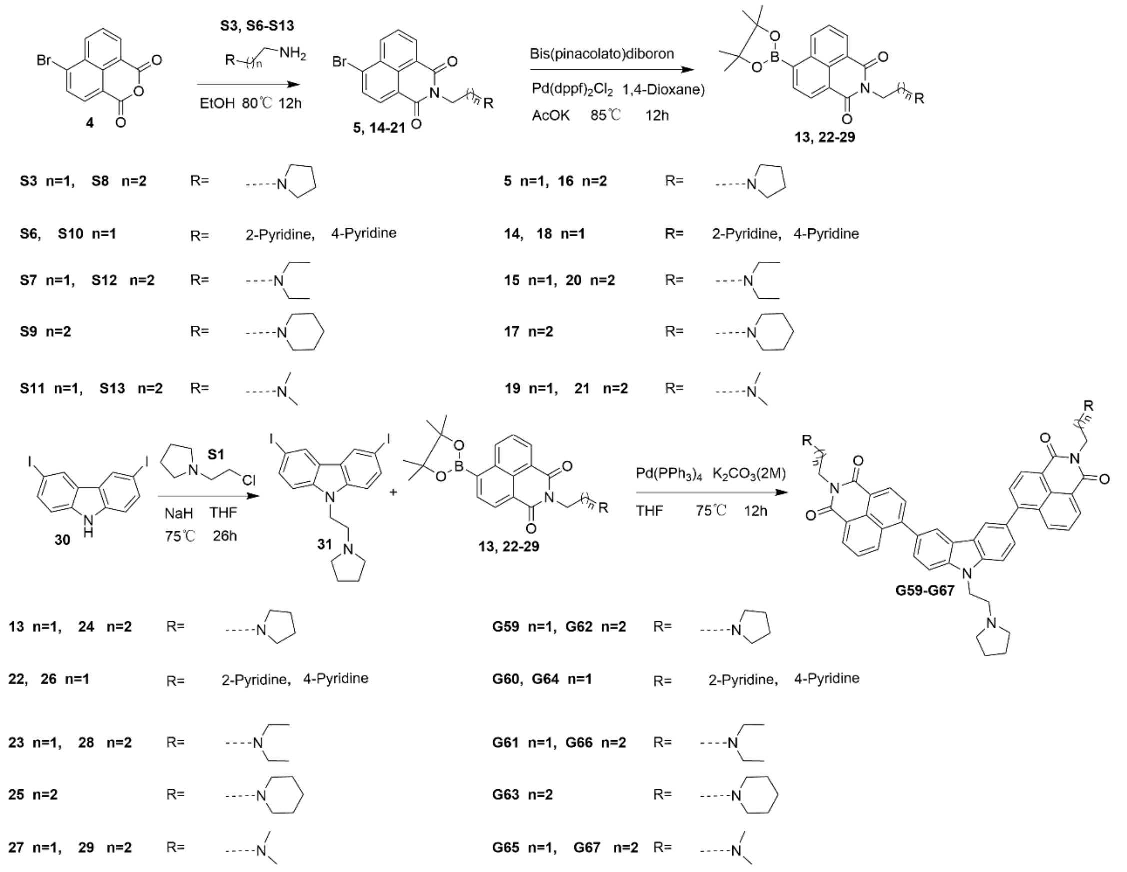

1.4.1. General procedures for syntheses of **14-21** were similar to that for compound **5**

1.4.2. General procedures for syntheses of **22-29** were similar to that for compound **13**

A mixture of **30** (500 mg, 1.19 mmol) and NaH (115 mg, 4.78 mmol) in 20 mL THF was stirred at room temperature for 2 h under nitrogen atmosphere. In another flask, a mixture of 1-(2-chloroethyl) pyrrolidine hydrochloride **S1** (609 mg, 3.58 mmol) and NaOH (1.5 g) in 5 mL water was prepared at room temperature, and the upper layer of this solution was added dropwise to the previous mixture. The reaction mixture was heated at 75 °C for additional 24 h, and monitored by using TLC. The reaction was quenched with water, and the solvent was removed under reduced pressure. The solution was extracted with dichloromethane (3×20 mL), and the combined organic layer was washed with brine for three times, and dried over anhydrous sodium sulfate. After removal of the solvent under reduced pressure, the residue was purified by using column chromatography on silica gel with MeOH/CH_2Cl_2/NH_4OH (1:60:0.01) to give compound **31** as a white liquid (591 mg) with a yield of 96%. ^{1}H NMR (400 MHz, $CDCl_3$) δ 8.23 (d, J = 1.6 Hz, 2H), 7.67 (dd, J = 8.6, 1.7 Hz, 2H), 7.14 (d, J = 8.6 Hz,

2H), 4.34 – 4.23 (m, 2H), 2.87 – 2.74 (m, 2H), 2.57 (dd, J = 8.5, 3.1 Hz, 4H), 1.83 – 1.73 (m, 4H). ^{13}C NMR (100 MHz, $CDCl_3$) δ 139.33, 134.58, 129.33, 123.98, 110.79, 81.93, 54.56, 53.96, 42.65, 23.58. ESI-MS (m/z) 517.05 $[M+H]^+$.

1.4.3. General procedure for syntheses of carbazole derivatives **G59-G67**

A mixture of **31** (150 mg, 0.29 mmol), 2M K_2CO_3 (3 mL), $Pd(PPh_3)_4$ (24 mg 0.02 mmol) and **13** (**22-29**) (366 mg, 0.87 mmol) in 20 mL absolute tetrahydrofuran was heated at 85 °C for 12 h under nitrogen atmosphere, and monitored by using TLC. The reaction was quenched with water, and the solvent was removed under reduced pressure. The solution was extracted with dichloromethane (3×20 mL), and the combined organic layer was washed with brine for three times, and dried over anhydrous sodium sulfate. After removal of the solvent under reduced pressure, the residue was purified by using column chromatography on silica gel with $MeOH/CH_2Cl_2/NH_4OH$ (1:50:0.01) to give compound **G59** as a yellow solid (198 mg) with a yield of 80%. ^1H NMR (400 MHz, $CDCl_3$) δ 8.66 – 8.57 (m, 4H), 8.39 – 8.33 (m, 2H), 8.25 (s, 2H), 7.79 (d, J = 7.6 Hz, 2H), 7.70 – 7.62 (m, 6H), 4.67 – 4.57 (m, 2H), 4.41 – 4.31 (m, 4H), 3.05 (s, 2H), 2.86 – 2.78 (m, 4H), 2.70 (d, J = 22.0 Hz, 12H), 1.87 (d, J = 3.3 Hz, 4H), 1.79 (d, J = 3.2 Hz, 8H). ^{13}C NMR (100 MHz, $CDCl_3$) δ 164.35, 164.16, 147.64, 140.77, 133.02, 131.14, 130.87, 130.43, 130.18, 128.85, 128.39, 128.27, 126.74, 123.04, 122.86, 122.12, 121.26, 109.12, 54.68, 54.38, 54.33, 53.66, 42.99, 39.21, 23.65, 23.62. HRMS (ESI) calculated for $C_{54}H_{52}N_6O_4$, $[M+H]^{2+}$, 425.2098; found, 425.2096.

Compound **G60** was similarly obtained as a yellow solid (156 mg) with a yield of 86%. ^1H NMR (400 MHz, $CDCl_3$) δ 8.66 (dd, J = 13.3, 7.4 Hz, 4H), 8.57 (d, J = 4.6 Hz, 2H), 8.41 (d, J = 8.4 Hz, 2H), 8.29 (s, 2H), 7.83 (d, J = 7.5 Hz, 2H), 7.70 (d, J = 8.3 Hz, 6H), 7.63 (t, J = 7.6 Hz, 2H), 7.32 (s, 2H), 7.20 – 7.11 (m, 2H), 4.66 (dd, J = 20.5, 13.0 Hz, 6H), 3.38 – 3.24 (m, 4H), 3.13 (s, 2H), 2.80 (s, 4H), 1.93 (s, 4H). ^{13}C NMR (100 MHz, $CDCl_3$) δ 164.22, 164.04, 158.99, 149.38, 147.63, 140.79, 136.38, 132.98, 131.16, 130.88, 131.02, 130.48, 130.27, 128.86, 128.39, 128.26, 126.73, 123.32, 123.08,

122.88, 122.11, 121.47, 121.30, 109.15, 54.65, 54.29, 40.21, 36.43, 23.65. HRMS (ESI) calculated for $C_{56}H_{44}N_6O_4$, $[M+H]^{2+}$, 433.1785; found, 433.1785.

Compound **G61** was similarly obtained as a yellow solid (157 mg) with a yield of 88%. ^{1}H NMR (500 MHz, $CDCl_3$) δ 8.64 (dd, J = 16.5, 7.4 Hz, 4H), 8.38 (d, J = 8.5 Hz, 2H), 8.26 (s, 2H), 7.81 (d, J = 7.5 Hz, 2H), 7.68 (dd, J = 9.3, 6.3 Hz, 6H), 4.68 – 4.61 (m, 2H), 4.37 – 4.30 (m, 4H), 3.09 – 3.04 (m, 2H), 2.85 – 2.80 (m, 4H), 2.74 (s, 4H), 2.70 (q, J = 7.1 Hz, 8H), 1.89 (s, 4H), 1.12 (t, J = 7.1 Hz, 12H). ^{13}C NMR (125 MHz, $CDCl_3$) δ 164.40, 164.21, 147.66, 140.82, 133.02, 131.12, 130.84, 130.50, 130.23, 128.87, 128.38, 128.30, 126.76, 123.07, 122.91, 122.12, 121.33, 109.15, 54.69, 54.35, 49.90, 47.68, 38.03, 23.65, 12.24. HRMS (ESI) calculated for $C_{54}H_{56}N_6O_4$, $[M+H]^{2+}$, 427.2254; found, 427.2256.

Compound **G62** was similarly obtained as a yellow solid (156 mg) with a yield of 85%. ^{1}H NMR (400 MHz, $CDCl_3$) δ 8.64 (dd, J = 12.9, 7.4 Hz, 4H), 8.38 (d, J = 8.4 Hz, 2H), 8.26 (s, 2H), 7.81 (d, J = 7.6 Hz, 2H), 7.68 (t, J = 7.9 Hz, 6H), 4.72 – 4.57 (m, 2H), 4.38 – 4.23 (m, 4H), 3.11 – 3.01 (m, 2H), 2.74 (s, 4H), 2.68 – 2.62 (m, 4H), 2.56 (s, 8H), 2.03 (dd, J = 14.5, 7.3 Hz, 4H), 1.89 (s, 4H), 1.75 (s, 8H). ^{13}C NMR (100 MHz, $CDCl_3$) δ 164.42, 164.24, 147.63, 140.80, 132.98, 131.13, 130.84, 130.47, 130.22, 128.84, 128.39, 128.28, 126.75, 123.06, 122.93, 122.11, 121.34, 109.13, 54.68, 54.34, 54.10, 54.01, 43.00, 38.98, 29.71, 27.37, 23.65, 23.43. HRMS (ESI) calculated for $C_{56}H_{56}N_6O_4$, $[M+H]^{2+}$, 439.2254; found, 439.2253.

Compound **G63** was similarly obtained as a yellow solid (152 mg) with a yield of 80%. ^{1}H NMR (400 MHz, $CDCl_3$) δ 8.64 (dd, J = 12.7, 7.4 Hz, 4H), 8.38 (d, J = 8.5 Hz, 2H), 8.26 (s, 2H), 7.80 (d, J = 7.6 Hz, 2H), 7.74 – 7.64 (m, 6H), 4.74 – 4.58 (m, 2H), 4.30 – 4.19 (m, 4H), 3.11 – 3.01 (m, 2H), 2.74 (s, 4H), 2.53 – 2.47 (m, 4H), 2.41 (s, 8H), 2.02 – 1.96 (m, 4H), 1.89 (d, J = 3.2 Hz, 4H), 1.54 (dt, J = 10.8, 5.5 Hz, 8H), 1.41 (dd, J = 9.5, 3.1 Hz, 4H). ^{13}C NMR (100 MHz, $CDCl_3$) δ 164.42, 164.24, 147.59, 140.81,

132.95, 131.11, 130.82, 130.47, 130.24, 128.85, 128.38, 128.27, 126.74, 123.06, 122.97, 122.11, 121.39, 109.13, 56.83, 54.68, 54.49, 54.36, 43.02, 39.07, 25.89, 25.27, 24.42, 23.66. HRMS (ESI) calculated for $C_{58}H_{60}N_6O_4$, $[M+H]^{2+}$, 453.2411; found, 453.2412.

Compound **G64** was similarly obtained as a yellow solid (138 mg) with a yield of 76%. ^{1}H NMR (400 MHz, $CDCl_3$) δ 8.70 – 8.59 (m, 4H), 8.52 (d, J = 4.7 Hz, 4H), 8.40 (d, J = 8.4 Hz, 2H), 8.27 (s, 2H), 7.82 (d, J = 7.6 Hz, 2H), 7.75 – 7.59 (m, 6H), 7.29 (d, J = 5.7 Hz, 4H), 4.73 – 4.59 (m, 2H), 4.45 (dd, J = 8.9, 6.8 Hz, 4H), 3.14 – 2.96 (m, 6H), 2.75 (s, 4H), 1.89 (s, 4H). ^{13}C NMR (100 MHz, $CDCl_3$) δ 164.23, 164.04, 149.86, 147.92, 147.79, 140.82, 133.28, 131.31, 131.01, 130.51, 130.19, 128.84, 128.44, 128.36, 126.84, 124.41, 123.09, 122.63, 122.14, 121.02, 109.23, 54.65, 54.24, 42.82, 40.57, 33.57, 23.65. HRMS (ESI) calculated for $C_{56}H_{44}N_6O_4$, $[M+H]^{2+}$, 433.1785; found, 433.1785.

Compound **G65** was similarly obtained as a yellow solid (135 mg) with a yield of 81%. ^{1}H NMR (400 MHz, $CDCl_3$) δ 8.64 (dd, J = 12.6, 7.5 Hz, 4H), 8.37 (d, J = 8.5 Hz, 2H), 8.25 (s, 2H), 7.80 (d, J = 7.5 Hz, 2H), 7.68 (d, J = 9.4 Hz, 6H), 4.74 – 4.58 (m, 2H), 4.36 (t, J = 6.9 Hz, 4H), 3.11 – 2.99 (m, 2H), 2.70 (dd, J = 17.5, 10.6 Hz, 8H), 2.37 (s, 12H), 1.89 (s, 4H). ^{13}C NMR (100 MHz, $CDCl_3$) δ 164.45, 164.27, 147.68, 140.79, 133.05, 131.20, 130.92, 130.47, 130.22, 128.90, 128.40, 128.28, 126.75, 123.06, 122.87, 122.12, 121.28, 109.14, 56.98, 54.68, 54.31, 45.75, 42.95, 38.18, 23.65. HRMS (ESI) calculated for $C_{50}H_{48}N_6O_4$, $[M+H]^{2+}$, 399.1941; found, 399.1941.

Compound **G66** was similarly obtained as a yellow solid (133 mg) with a yield of 72%. ^{1}H NMR (400 MHz, $CDCl_3$) δ 8.65 (dd, J = 12.9, 7.4 Hz, 4H), 8.38 (d, J = 8.5 Hz, 2H), 8.26 (s, 2H), 7.81 (d, J = 7.6 Hz, 2H), 7.73 – 7.64 (m, 6H), 4.72 – 4.57 (m, 2H), 4.31 – 4.18 (m, 4H), 3.19 – 2.95 (m, 2H), 2.74 (s, 4H), 2.67 – 2.54 (m, 12H), 1.95 (dd, J = 15.0, 7.7 Hz, 4H), 1.89 (s, 4H), 1.04 (t, J = 7.1 Hz, 12H). ^{13}C NMR (100 MHz, $CDCl_3$) δ 164.41, 164.23, 147.65, 140.82, 133.01, 131.16, 130.87, 130.49, 130.23,

128.85, 128.39, 128.29, 126.76, 123.09, 122.93, 122.12, 121.34, 109.15, 54.69, 50.38, 46.76, 25.22, 23.65, 11.57. HRMS (ESI) calculated for $C_{56}H_{60}N_6O_4$, $[M+H]^{2+}$, 441.2411; found, 441.2410.

Compound **G67** was similarly obtained as a yellow solid (136 mg) with a yield of 79%. ^1H NMR (400 MHz, $CDCl_3$) δ 8.66 (dd, J = 12.8, 7.4 Hz, 4H), 8.40 (d, J = 8.5 Hz, 2H), 8.28 (s, 2H), 7.82 (d, J = 7.6 Hz, 2H), 7.75 – 7.65 (m, 6H), 4.77 – 4.61 (m, 2H), 4.33 – 4.23 (m, 4H), 3.17 – 3.02 (m, 2H), 2.76 (s, 4H), 2.48 (t, J = 7.2 Hz, 4H), 2.29 (s, 12H), 2.01 – 1.89 (m, 8H). ^{13}C NMR (100 MHz, $CDCl_3$) δ 164.38, 164.20, 147.63, 140.82, 132.98, 131.12, 130.84, 130.48, 130.22, 128.85, 128.38, 128.27, 126.74, 123.06, 122.93, 122.10, 121.34, 109.14, 57.30, 54.67, 54.35, 45.36, 43.02, 38.84, 26.11, 23.66. HRMS (ESI) calculated for $C_{52}H_{52}N_6O_4$, $[M+H]^{2+}$, 413.2098; found, 413.2098.

2. Table S1. Oligomers or primers used in this study

**Oligomer**	**Sequence**
c-myc	5′-CCCCACCTTCCCCACCCTCCCCACCCTCCCC-3′
5′-biotin c-myc	5′-biotin-CCCCACCTTCCCCACCCTCCCCACCCTCCCC-3′
PDGF-A	5′-CCGCGCCCCTCCCCCGCCCCCGCCCCCGCCCCCCCCCCCCC-3′
C-kit	5′-CCCTCCTCCCAGCGCCCACCCT-3′
bcl-2	5′-CAGCCCCGCTCCCGCCCCCTTCCTCCCGCGCCCGCCCCT-3′
ILPR	5′-TGTCCCCACACCCCTGTCCCCACACCCCTGT-3′
VEGF-A	5′-GACCCCGCCCCCGGCCCGCCCCGG-3′
c-myb	5′-TCCTCCTCCTCCTTCTCCTCCTCCTCCGTGTCCTCCTCCTCC-3′
RET	5′-CCGCCCCCGCCCCGCCCCGCCCCTA-3′
n-myc	5′-ACCCCCTGCATCTGCATGCCCCCTCCCACCCCCT-3′
Hras	5′-CGCCCGTGCCCTGCGCCCGCAACCCGA-3′
c-myc-C7A	5′-CCCCACATTCCCCACCCTCCCCACCCTCCCC-3′
c-myc-C7T	5′-CCCCACTTTCCCCACCCTCCCCACCCTCCCC-3′
c-myc-C7G	5′-CCCCACGTTCCCCACCCTCCCCACCCTCCCC-3′
c-myc-C16A	5′-CCCCACCTTCCCCACACTCCCCACCCTCCCC-3′

c-myc-C17T	5′-CCCCACCTTCCCCACTCTCCCCACCCTCCCC-3′
c-myc-C16G	5′-CCCCACCTTCCCCACGCTCCCCACCCTCCCC-3′
c-myc-C25A	5′-CCCCACCTTCCCCACCCTCCCCACACTCCCC-3′
c-myc-C25T	5′-CCCCACCTTCCCCACCCTCCCCACTCTCCCC-3′
c-myc-C25G	5′-CCCCACCTTCCCCACCCTCCCCACGCTCCCC-3′
c-myc	5′-TTGAGGGTGGGTAGGGTGGGTAAA-3′
5′-biotin	5′-biotin-TTGAGGGTGGGTAGGGTGGGTAAA-3′
Pu27	5′-TGGGGAGGGTGGGGAGGGTGGGGAAGG-3′
pu22	5′-TGAGGGTGGGGAGGGTGGGGAA-3′
bcl2	5′-GGGCGGGCGCGGGAGGAAGGGGGCGGG-3′
C-kit	5′-GGCGAGGAGGGGCGTGGCCGGC-3′
VEGF	5′-GGGGCGGGCCGGGGGCGGGG-3′
Pu18	5′-AGGGTGGGGAGGGTGGGG-3′
RET	5′-GGGGCGGGGCGGGGCGGGGG-3′
ILPR	5′-ACAGGGGTGTGGGGACAGGGGTGTGGGG-3′
PDGF-A	5′GGAGGCGGGGGGGGGGGGCGGGGGCGGGGGCGGGGGAGGGGCGCGGC-3′
Hras	5′-TCGGGTTGCGGGCGCAGGGCACGGGCG-3′
c-myb	5′-AGGGAGTCGGGCAGGGGTGCTGGGA-3′
DS26	5′-CAATCGGATCGAATTCGATCCGATTG-3′
SS26	5′-ATACGATGCTTCACGGTGCTATCTG-3′

3. Table S2. Fluorescence intensity of **G59** in the presence of loop-mutated c-myc i-motif

name	sequence	FI/F_0
c-myc	5′-CCCCACCTTCCCCACCCTCCCCACCCTCCCC-3′	17.98
c-myc-C7A	5′-CCCCACATTCCCCACCCTCCCCACCCTCCCC-3′	13.35
c-myc-C7T	5′-CCCCACTTTCCCCACCCTCCCCACCCTCCCC-3′	18.35
c-myc-C7G	5′-CCCCACGTTCCCCACCCTCCCCACCCTCCCC-3′	5.44
c-myc-C16A	5′-CCCCACCTTCCCCACACTCCCCACCCTCCCC-3′	15.2

c-myc-C16T	5′-CCCCACCTTCCCCACTCTCCCCACCCTCCCC-3′	18.23
c-myc-C16G	5′-CCCCACCTTCCCCACGCTCCCCACCCTCCCC-3′	6.03
c-myc-C25A	5′-CCCCACCTTCCCCACCCTCCCCACACTCCCC-3′	17.15
c-myc-C25T	5′-CCCCACCTTCCCCACCCTCCCCACTCTCCCC-3′	17.92
c-myc-C25G	5′-CCCCACCTTCCCCACCCTCCCCACGCTCCCC-3′	6.59

4. Table S3. Summary of thermodynamic parameters for the binding of **G59**[a]

substance	stoichiometry	K_a (M^{-1})	K_D (μM)	ΔH°(kcal/mol)	ΔS°(kcal/mol)	ΔG° (kcal/mol)
**G59**	2.22	5.94×10^5	1.68	-6196	5.98	-4384.06

[a]Binding affinity was obtained by following equation: $K_D = 1/K_a$. The binding free energy (ΔG°) was calculated by following equation: ΔG° = ΔH° – TΔS°

5. Fluorescence property of carbazole derivatives

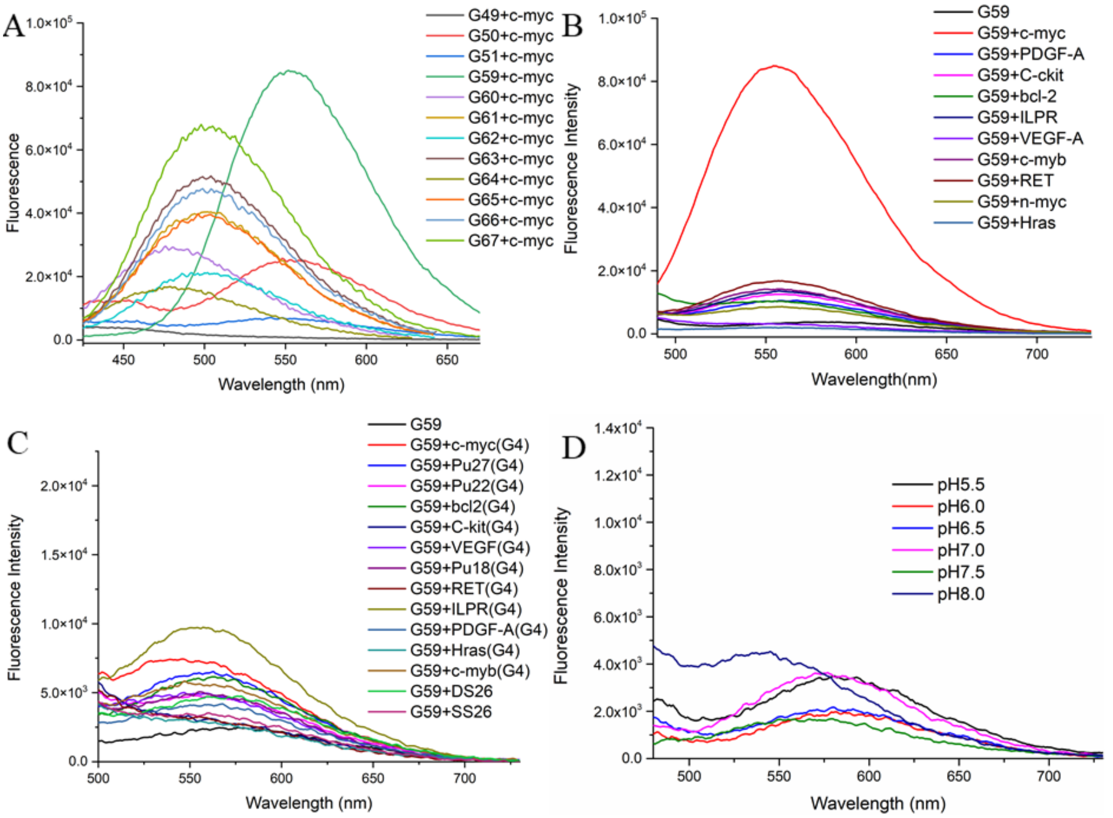

Fig. S1. A) Fluorescent spectra of c-myc promoter i-motif (2 μM) with or without our carbazole derivatives; B) Fluorescence intensity of 1 μM **G59** with 2 μM different i-motifs, in 1×BPES buffer at pH 5.5, with λex at 407 nm; C) Fluorescence intensity of 1 μM **G59** with 2 μM different G-

quadruplexes and other DNAs, in 20 mM Tris–HCl buffer at pH 7.4 containing 100 mM KCl, with λex at 407 nm; D) Fluorescent spectra of 1 μM **G59** under various pH conditions as a control experiment.

6. CD spectra of c-myc promoter i-motif

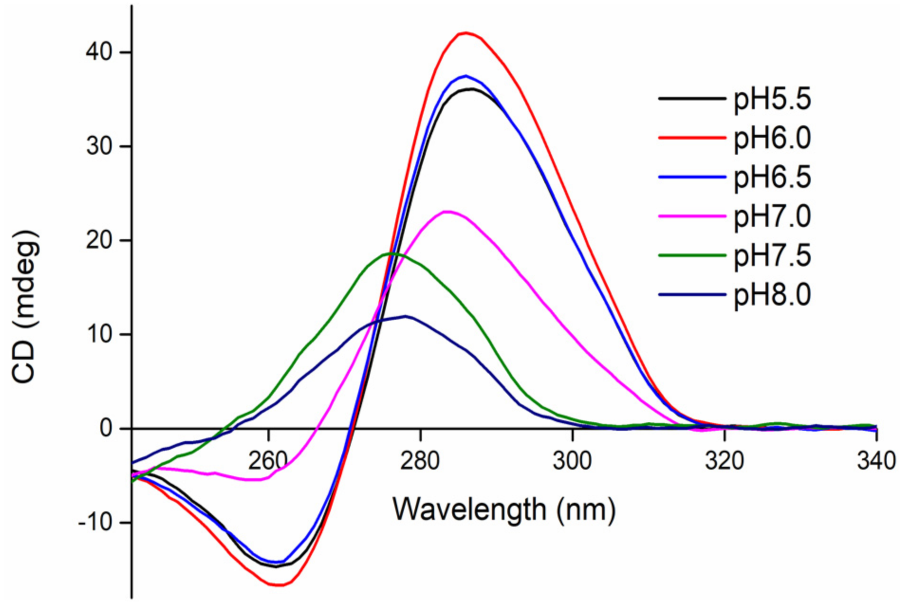

Fig. S2. CD spectra of c-myc promoter i-motif in 1×BPES buffer at different pH values.

7. CD spectra of wild type and mutant c-myc promoter i-motif

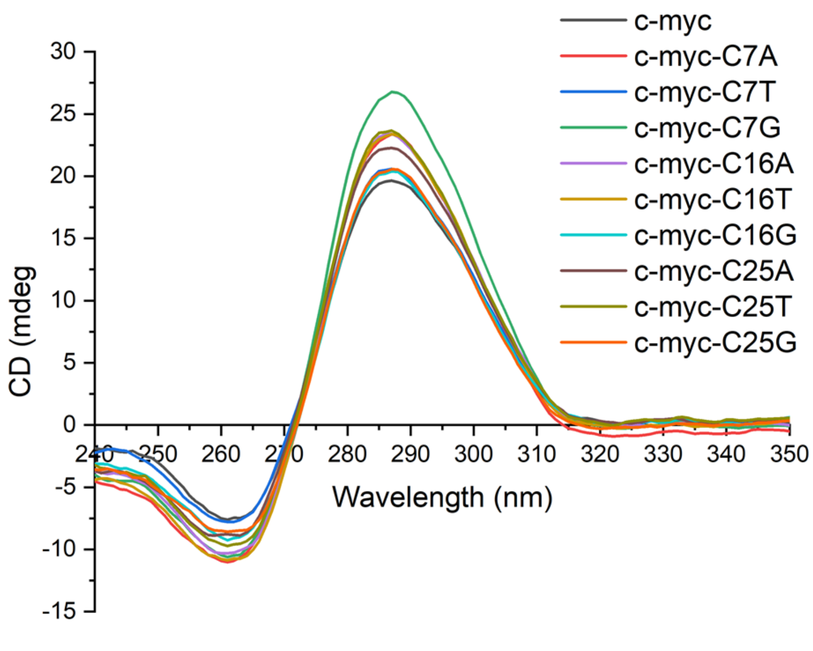

Fig. S3. CD spectra of c-myc promoter wild type and mutant i-motif in 1× BPES buffer at pH5.5.

8. ITC experiment for interaction between c-myc promoter i-motif and **G59**

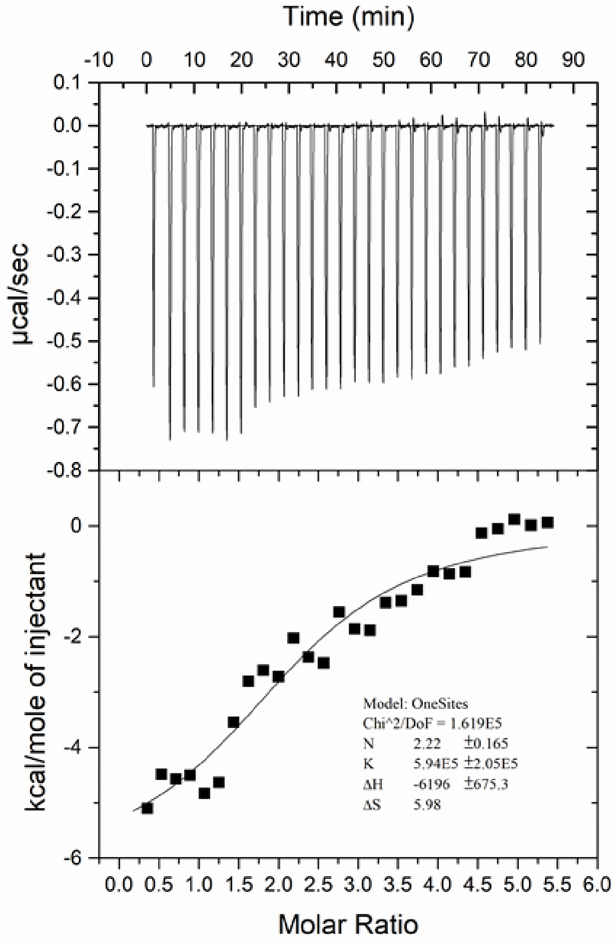

Fig. S4. ITC experiment was carried out for interaction between c-myc promoter i-motif and **G59**.

Top panel showed raw titration data, and bottom panel showed integrated binding isotherms.

9. Table S4. Relative replacement ratio of **G59** by using FID assay

Number	Chemical structure	Relative replacement ratio
**3be**	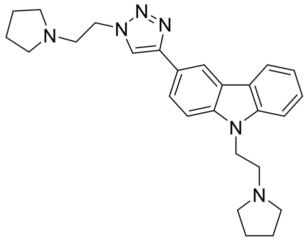	39.08±0.02%

**S876**	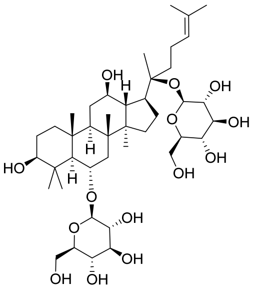<br>(ginsenoside Rg1)	3.34±0.03%
**S902**	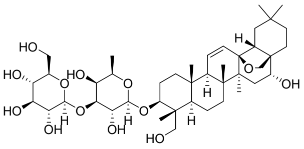<br>(Saikosaponin D)	5.75±0.06%
**S872**	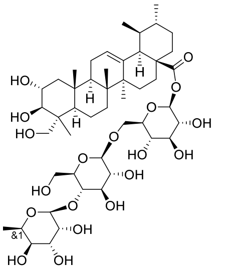<br>(Asiaticoside)	0.26±0.05%
**S884**	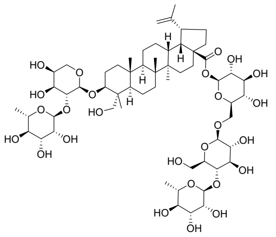<br>(Pulchinenoside B4)	0.85±0.03%
**S894**	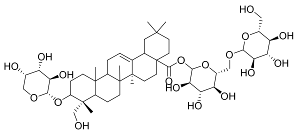<br>(Akebia saponin D)	0.79±0.01%
**S893**	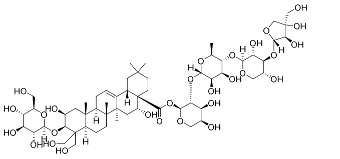<br>(Platycodin D)	0.20±0.02%

**S886**	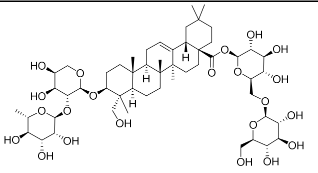 (Dipsacoside B)	2.44±0.01%
**S857**	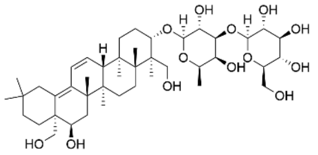 (Saikosaponin B2)	17.42±0.04%
**S188**	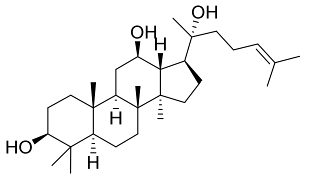 ((20S)-protopanaxadiol)	2.33±0.10%
**S895**	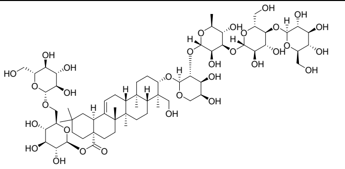 (macranthoidin B)	1.59±0.01%
**S879**	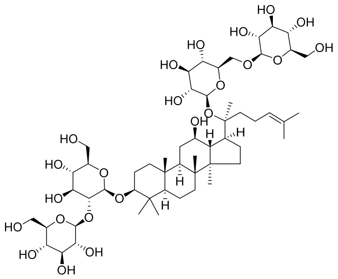 (Ginsenoside Rb1)	6.20±0.08%
**S885**	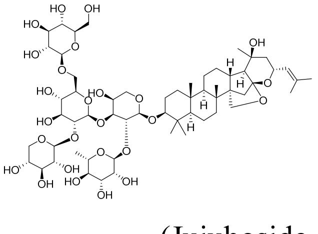 (Jujuboside A)	4.97±0.01%
**S877**	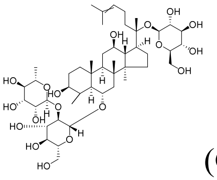 (Ginsenoside Re)	7.63±0.03%

**S168**	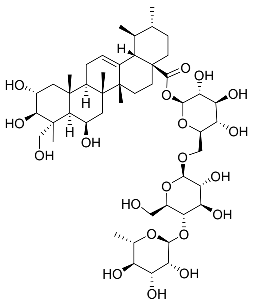<br>(Madecassoside)	4.29±0.02%

10. SPR experiment for interaction between c-myc promoter G-quadruplex and **S857**

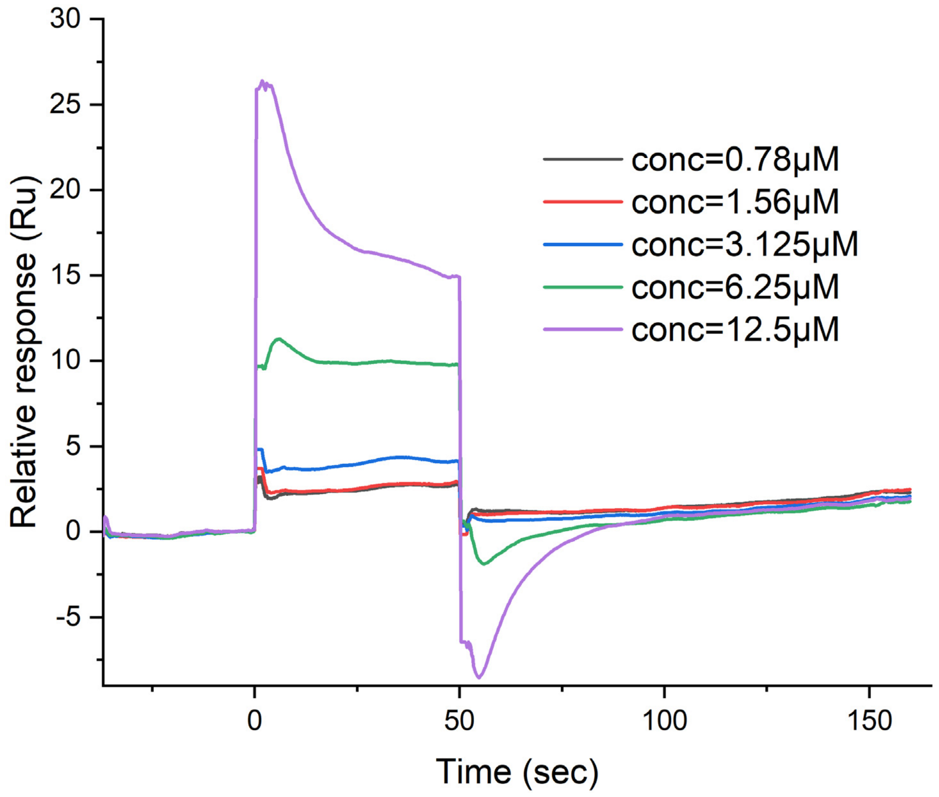

Fig. S5. Binding affinity of **S857** to c-myc gene promoter G-quadruplex was studied by using SPR in Tris-HCl buffer at pH 7.4.

11. NMR spectra of compounds

^{1}H and ^{13}C NMR of **2**:

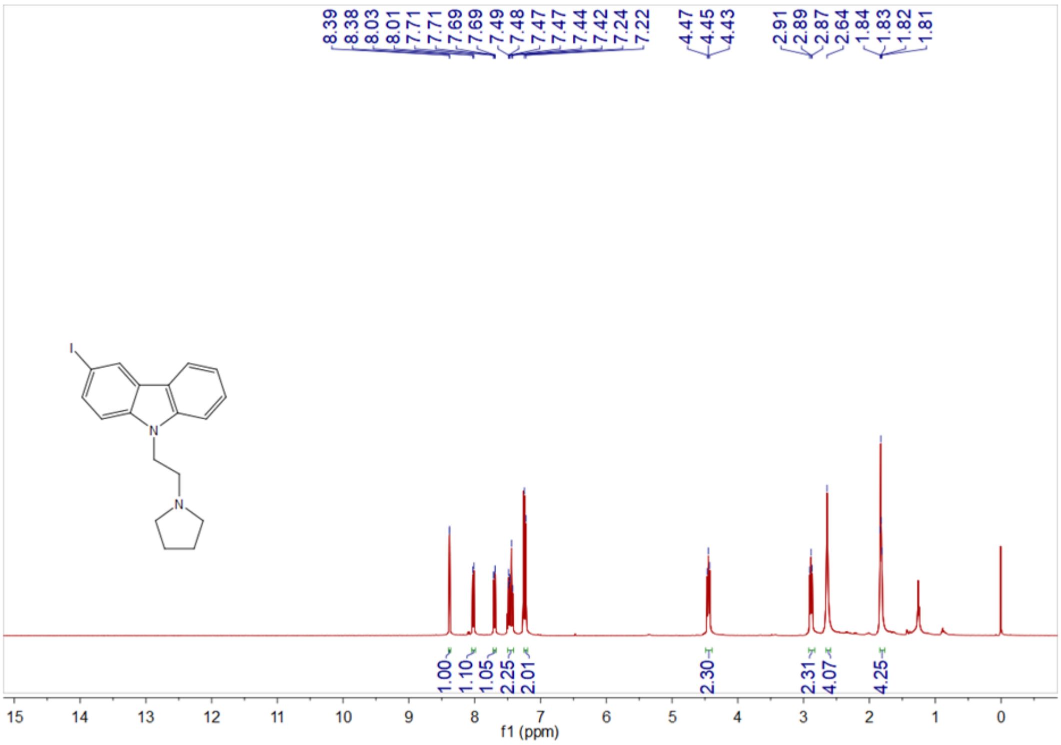

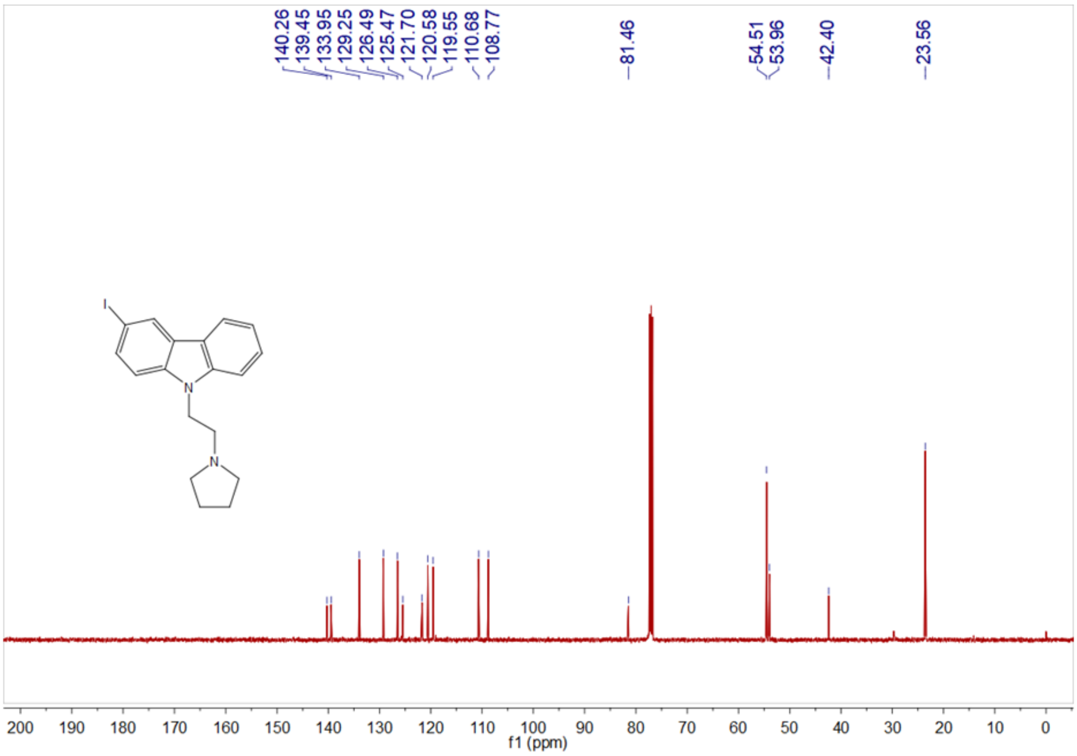

^{1}H and ^{13}C NMR of **3**:

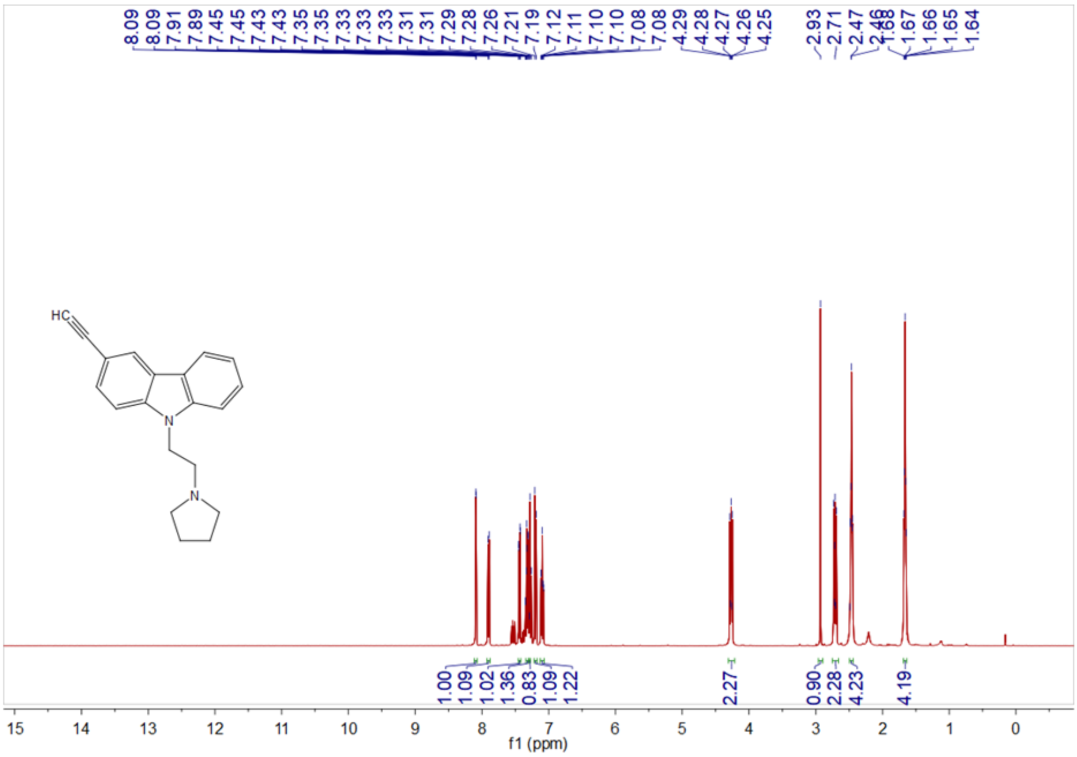

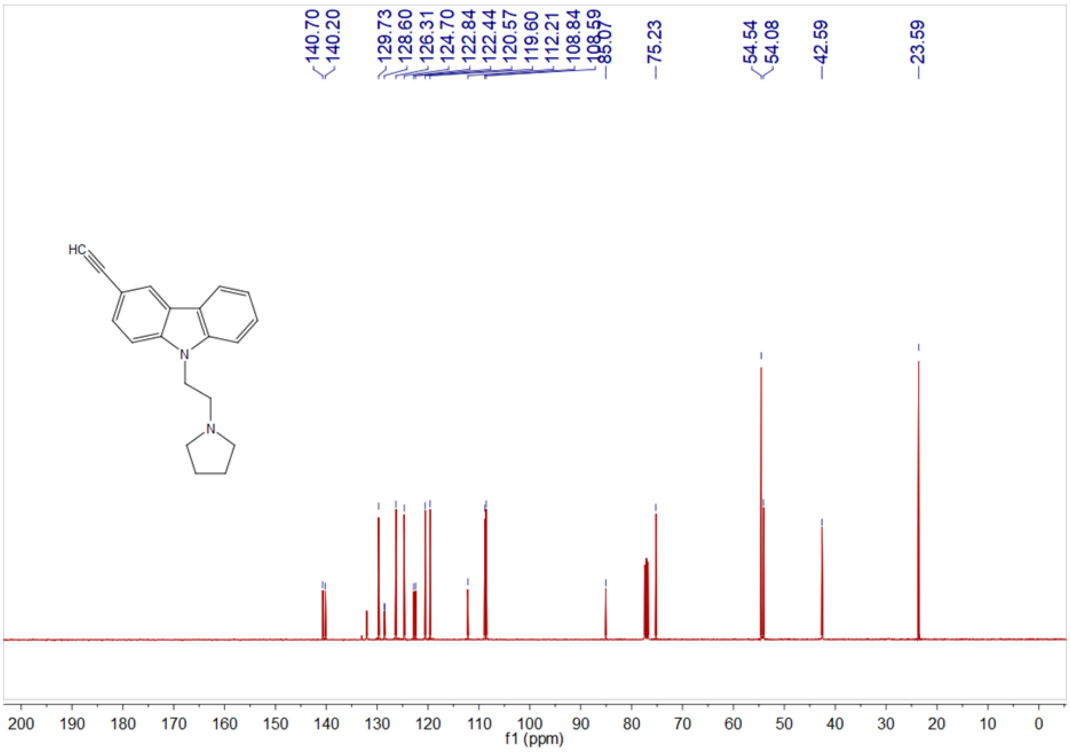

^{1}H and ^{13}C NMR of **5**:

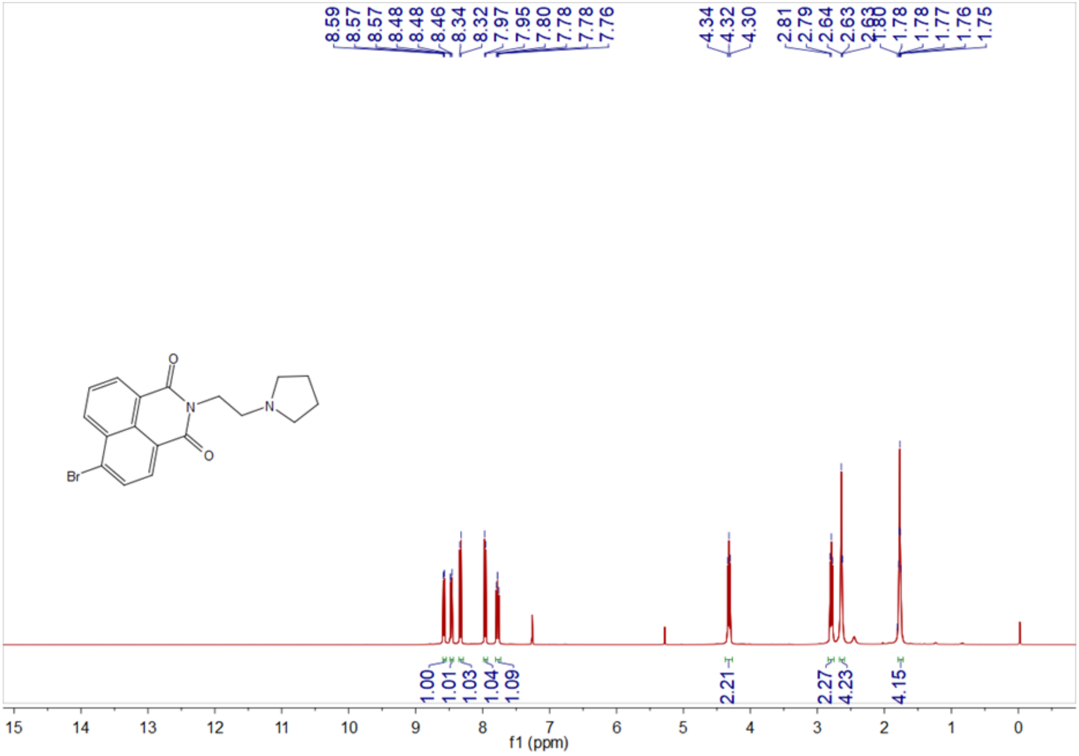

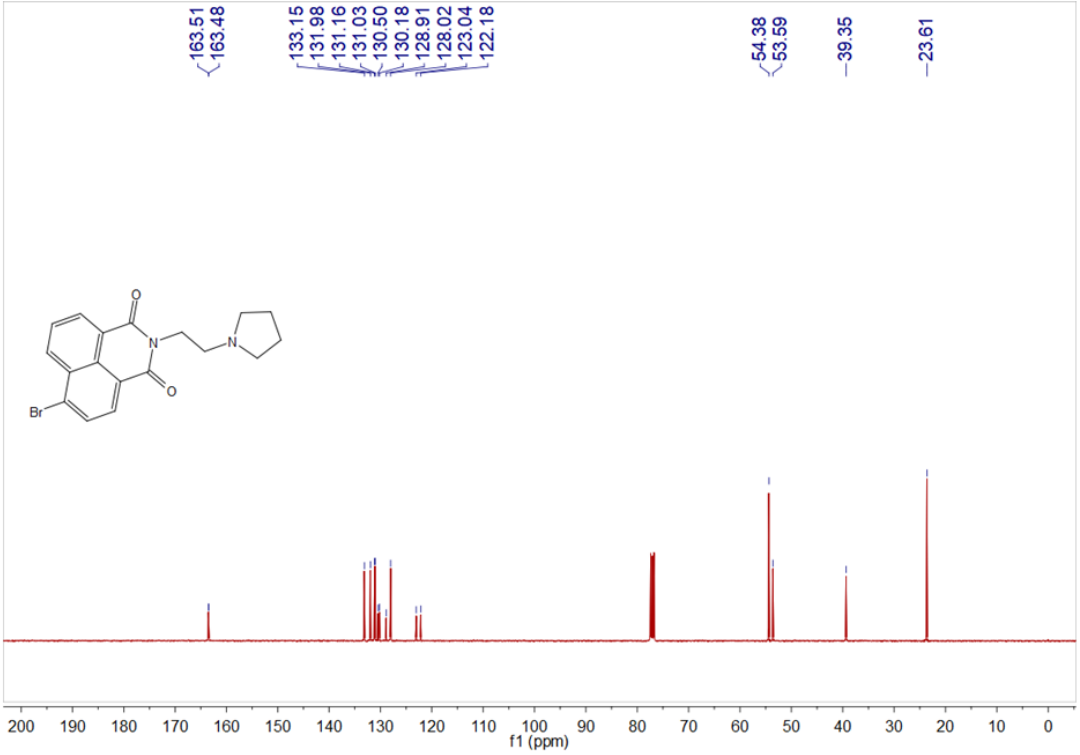

^{1}H and ^{13}C NMR of **7**:

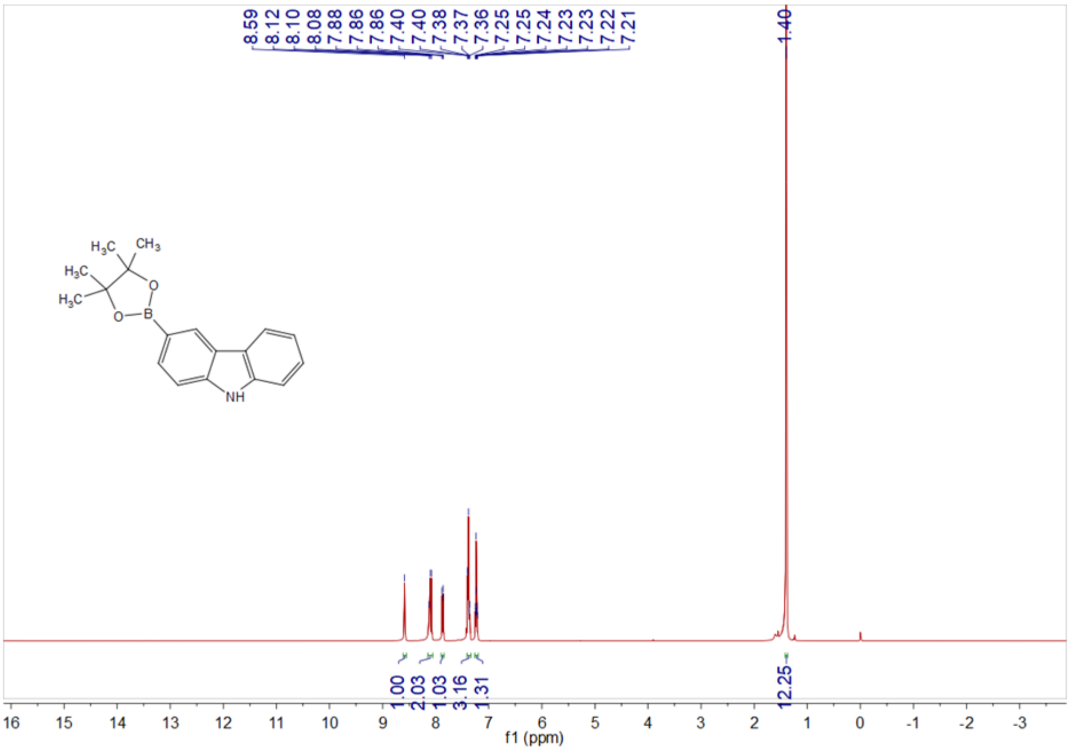

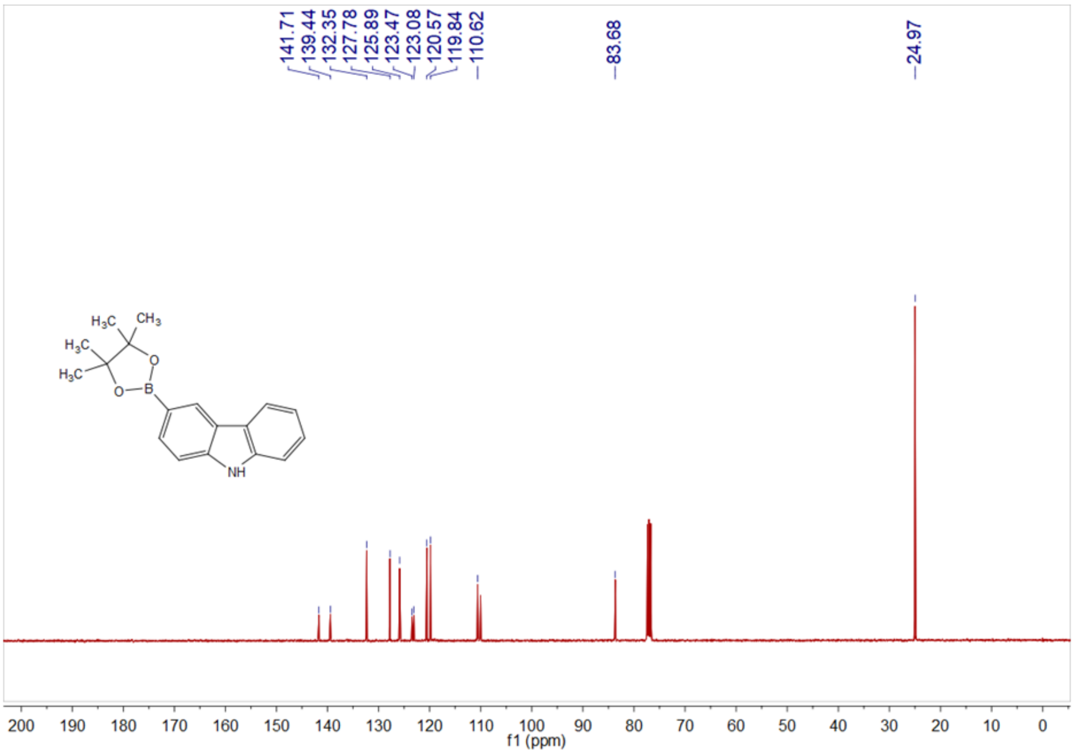

^{1}H and ^{13}C NMR of **8**:

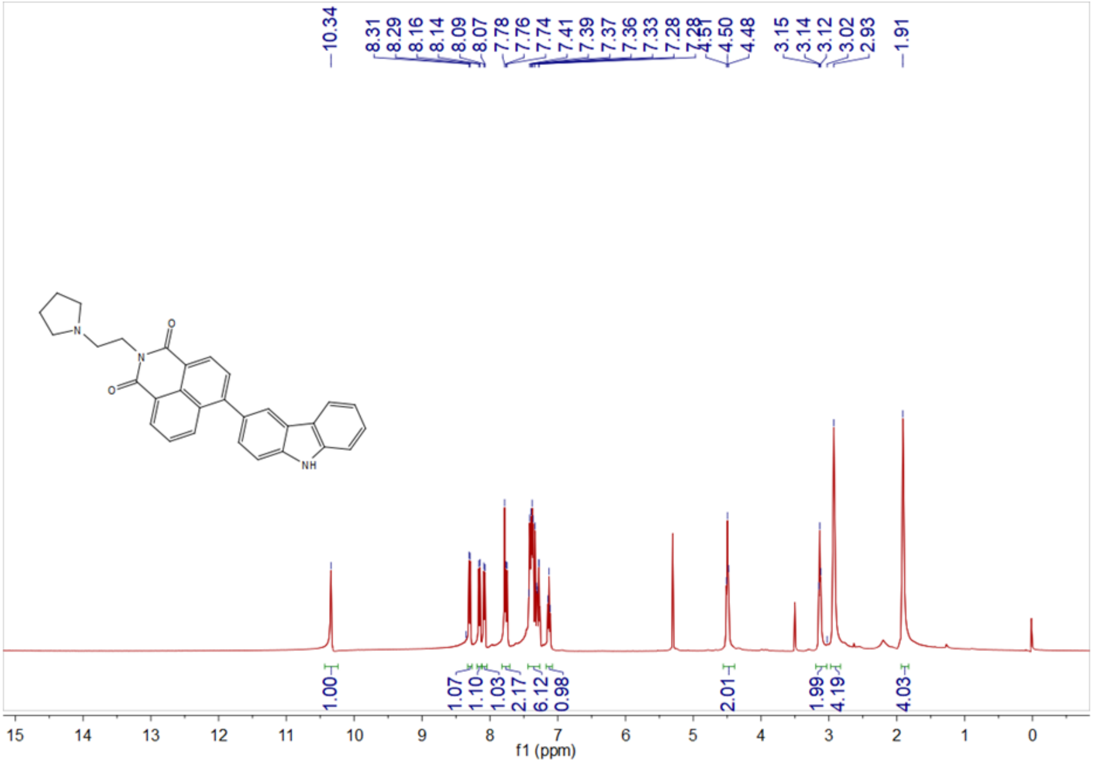

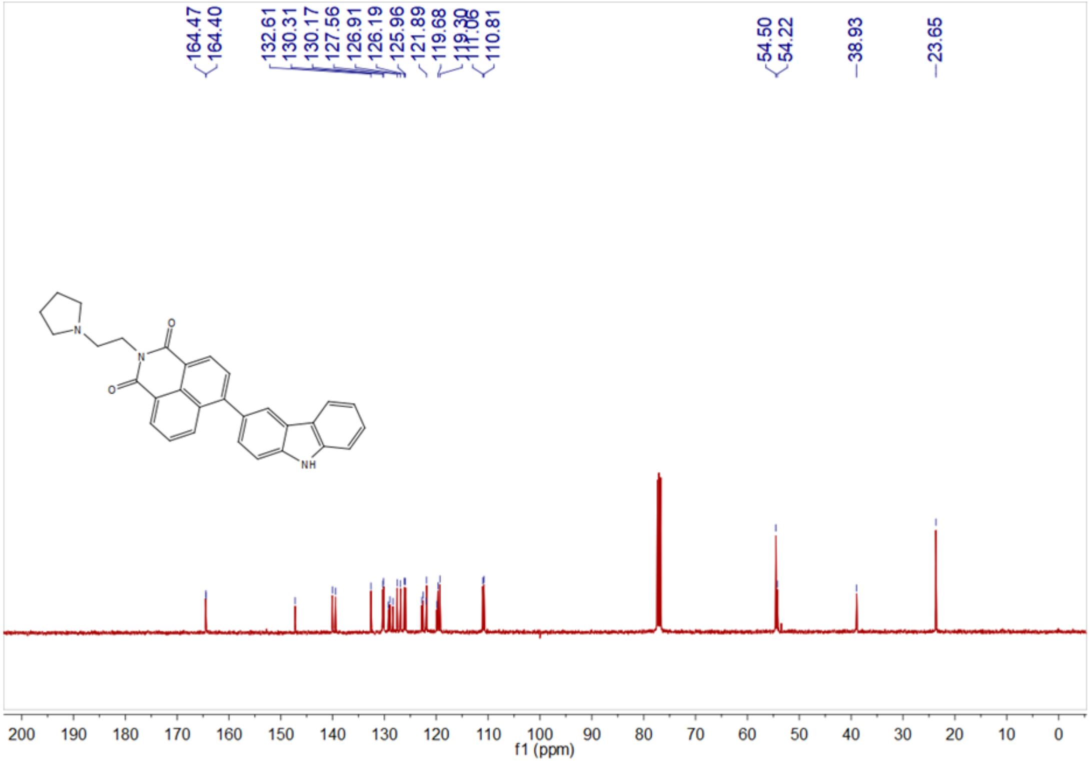

^{1}H and ^{13}C NMR of **9**:

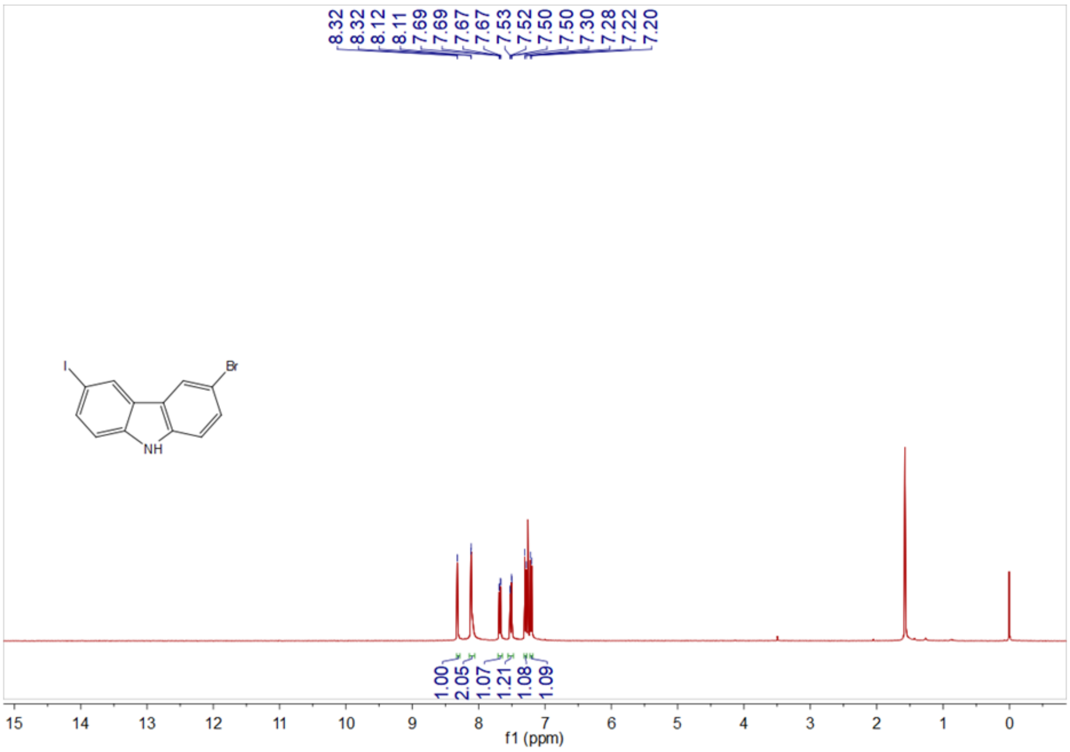

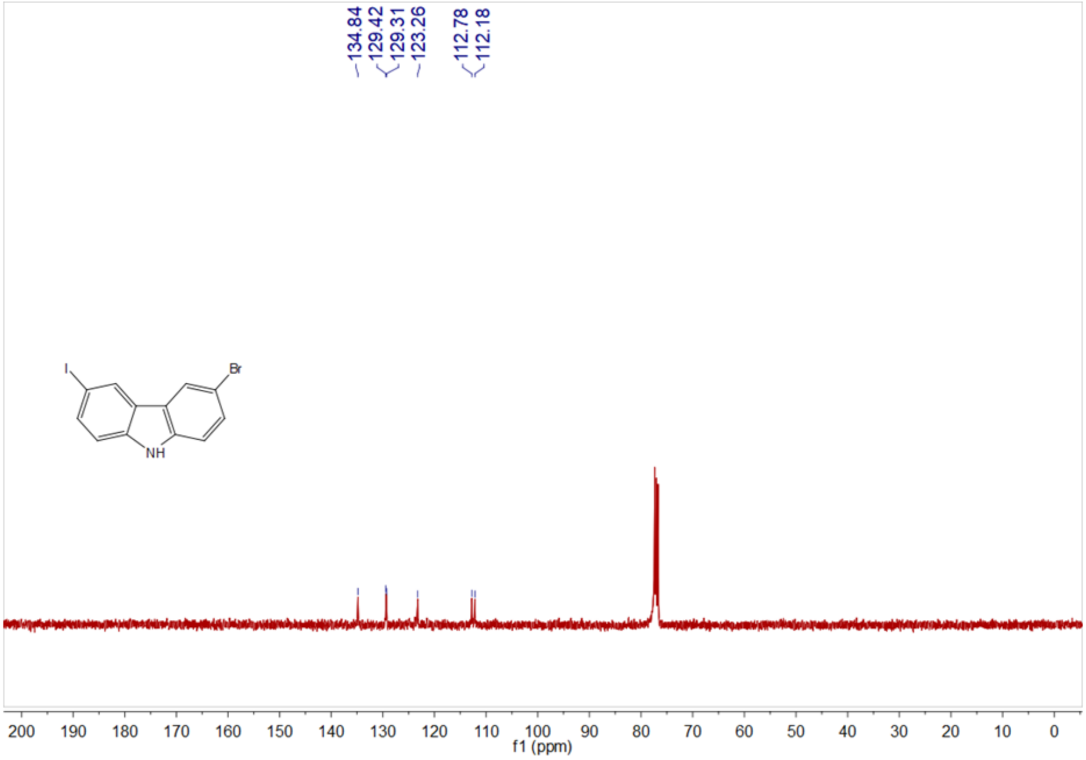

^{1}H and ^{13}C NMR of **10**:

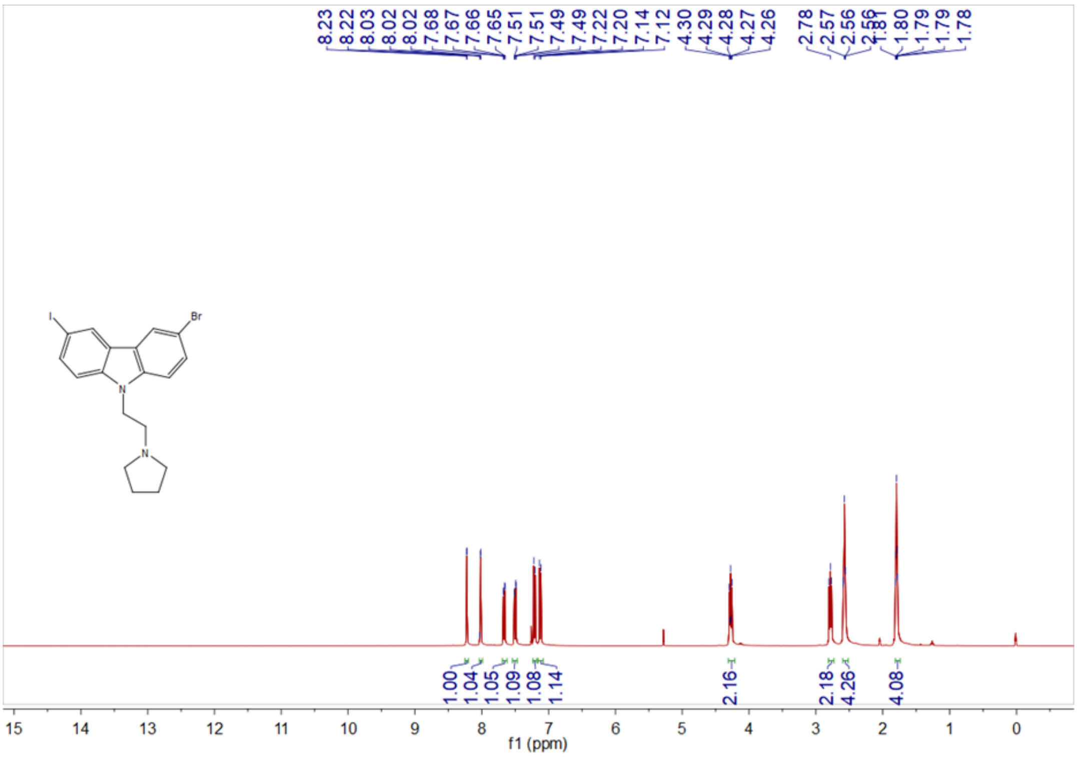

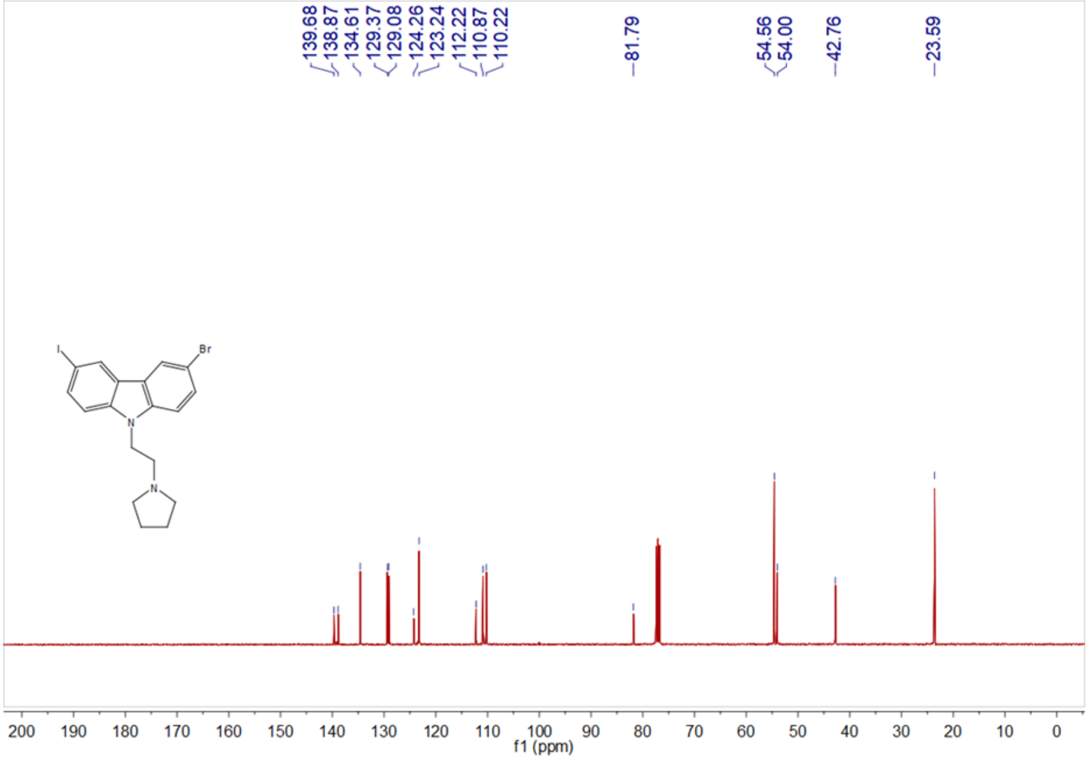

^{1}H and ^{13}C NMR of **11**:

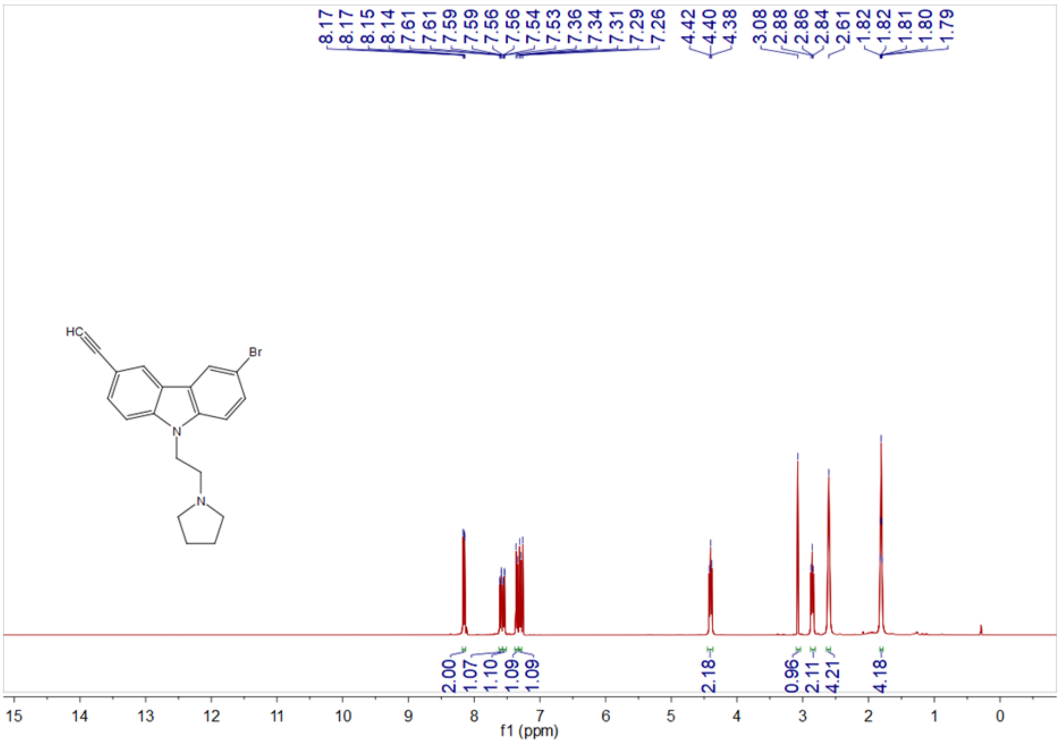

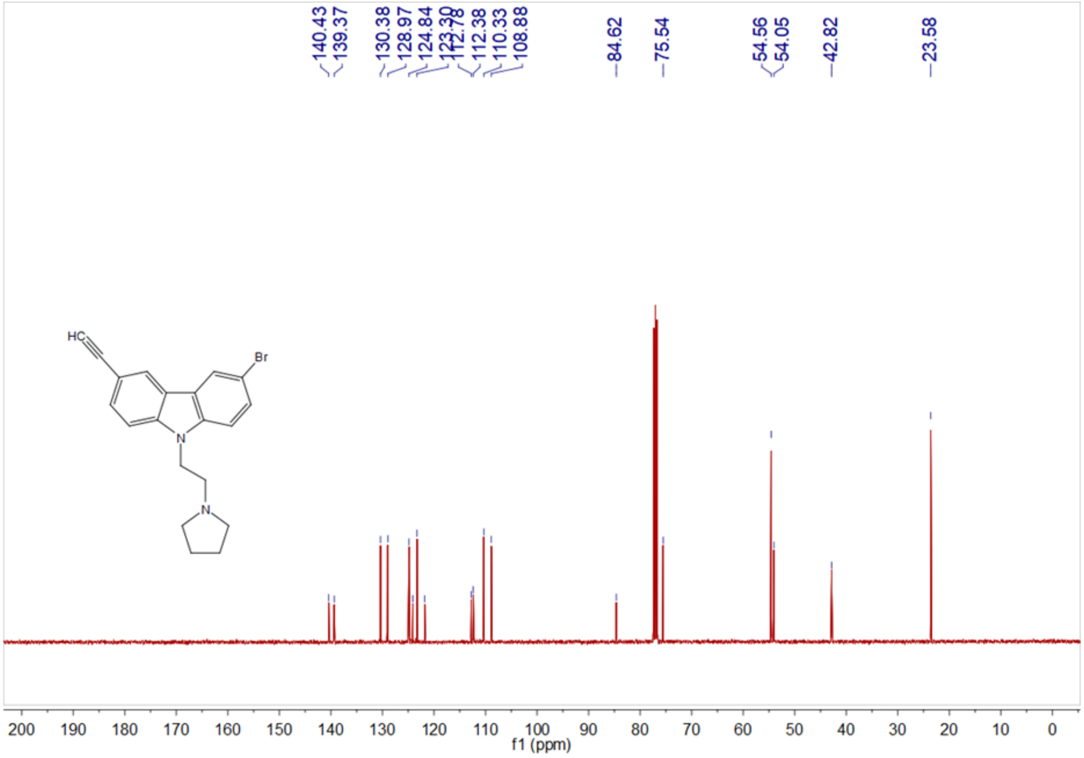

^{1}H and ^{13}C NMR of **12**:

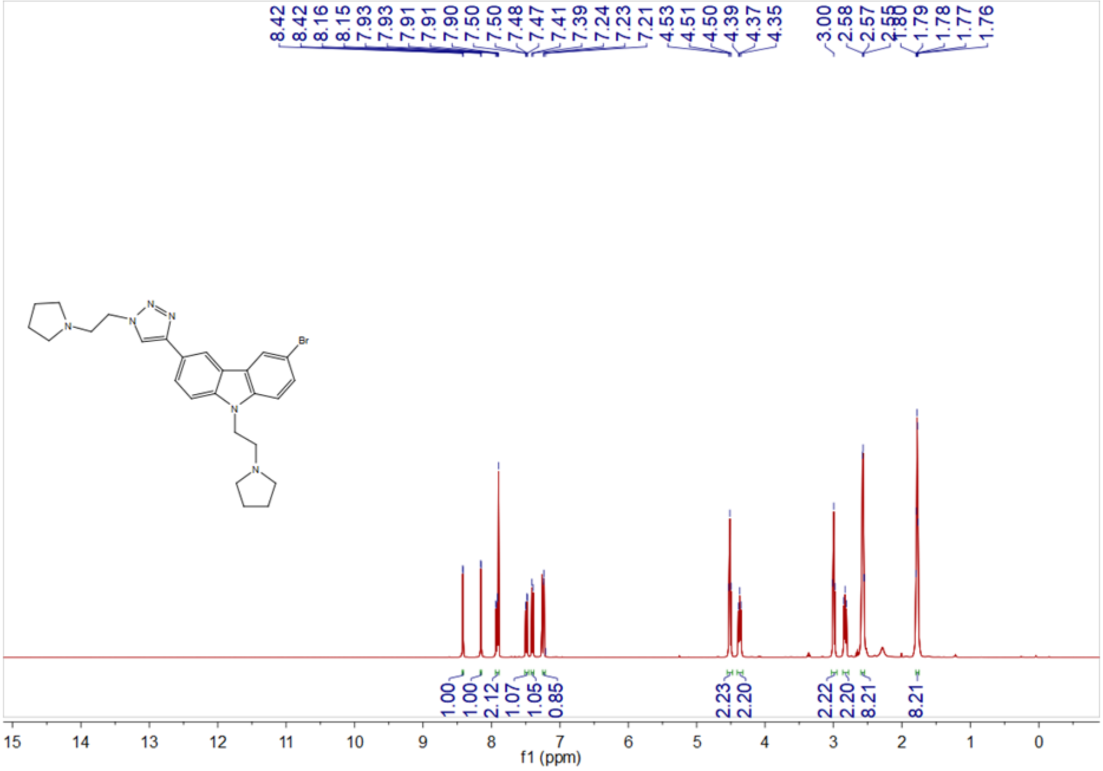

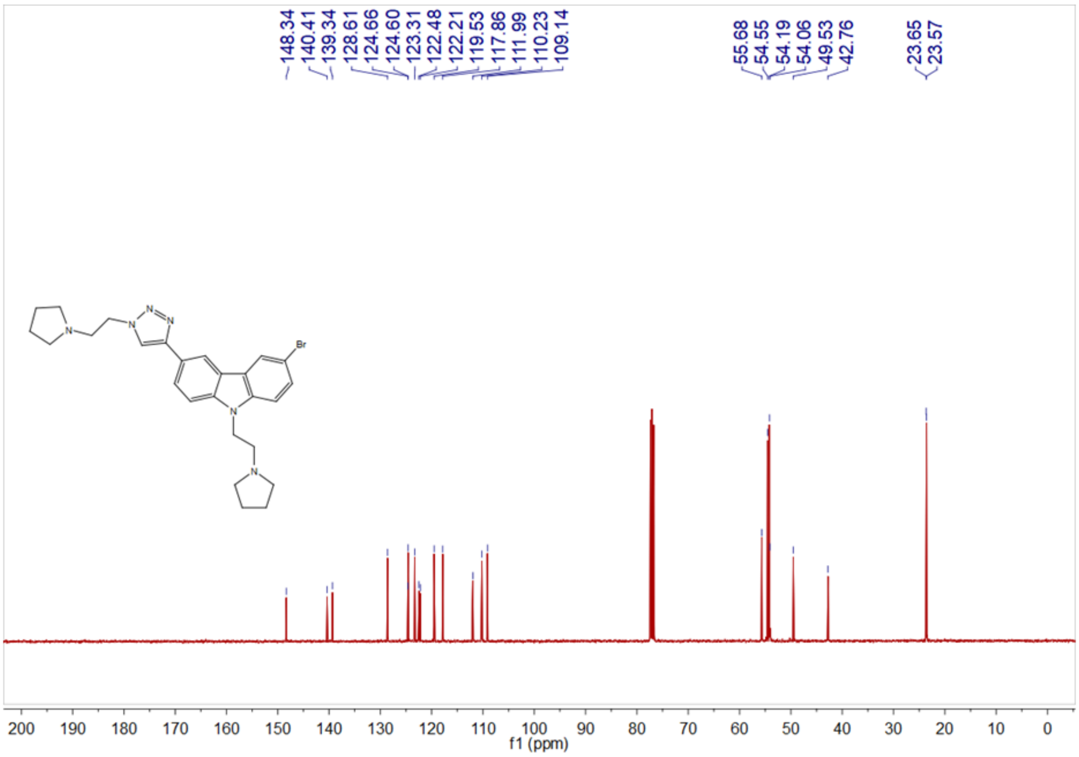

^{1}H and ^{13}C NMR of **15**:

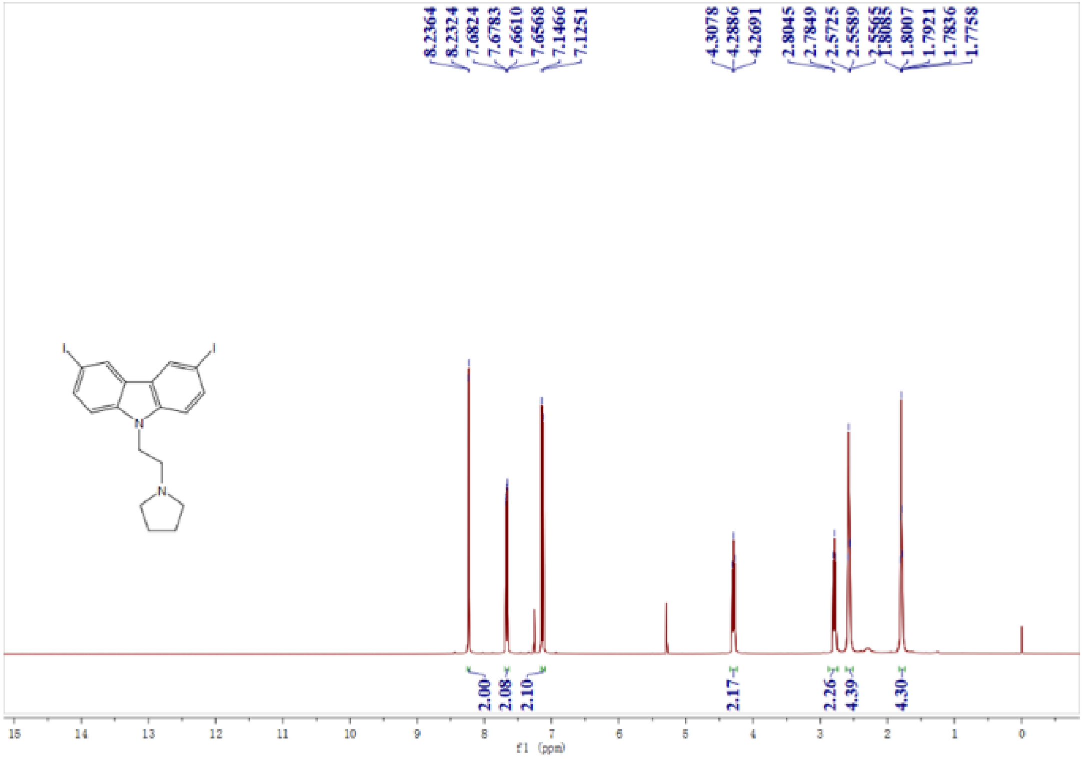

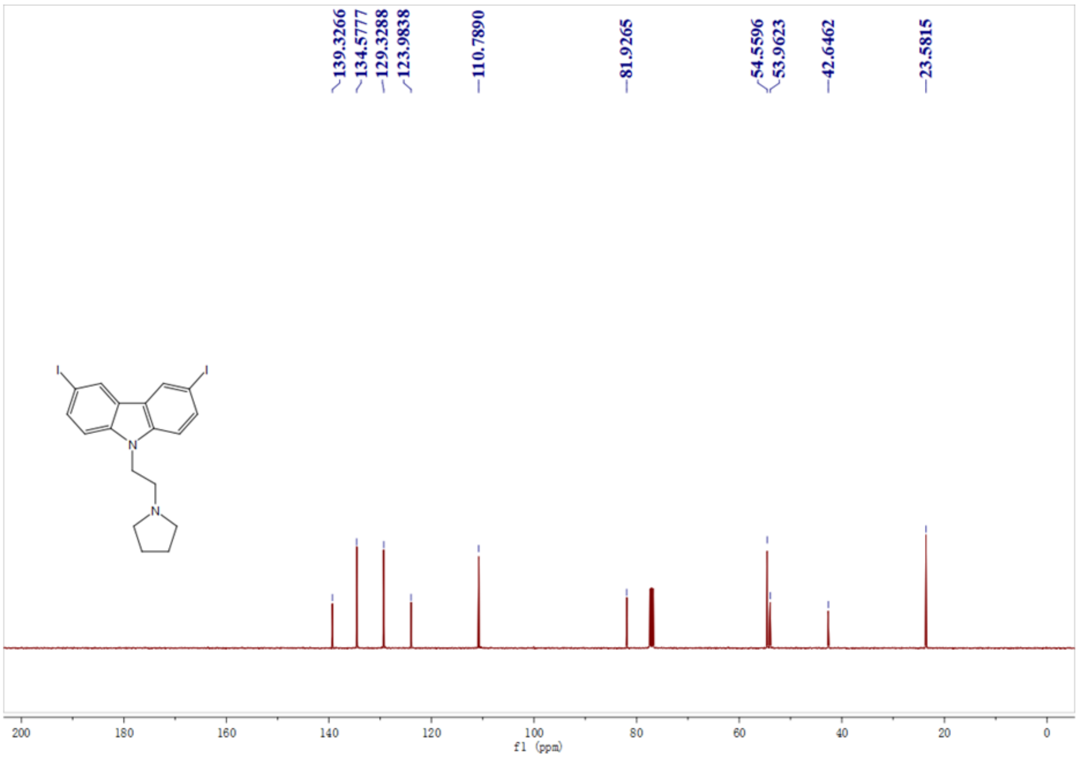

^{1}H and ^{13}C NMR of **G49**:

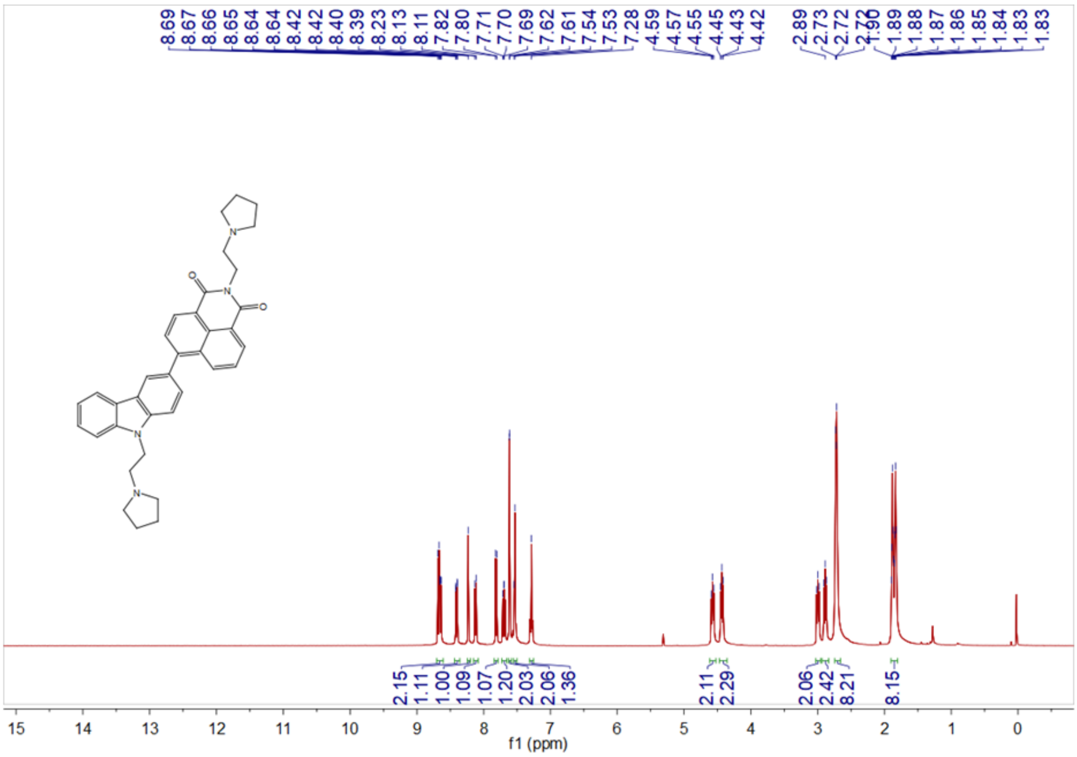

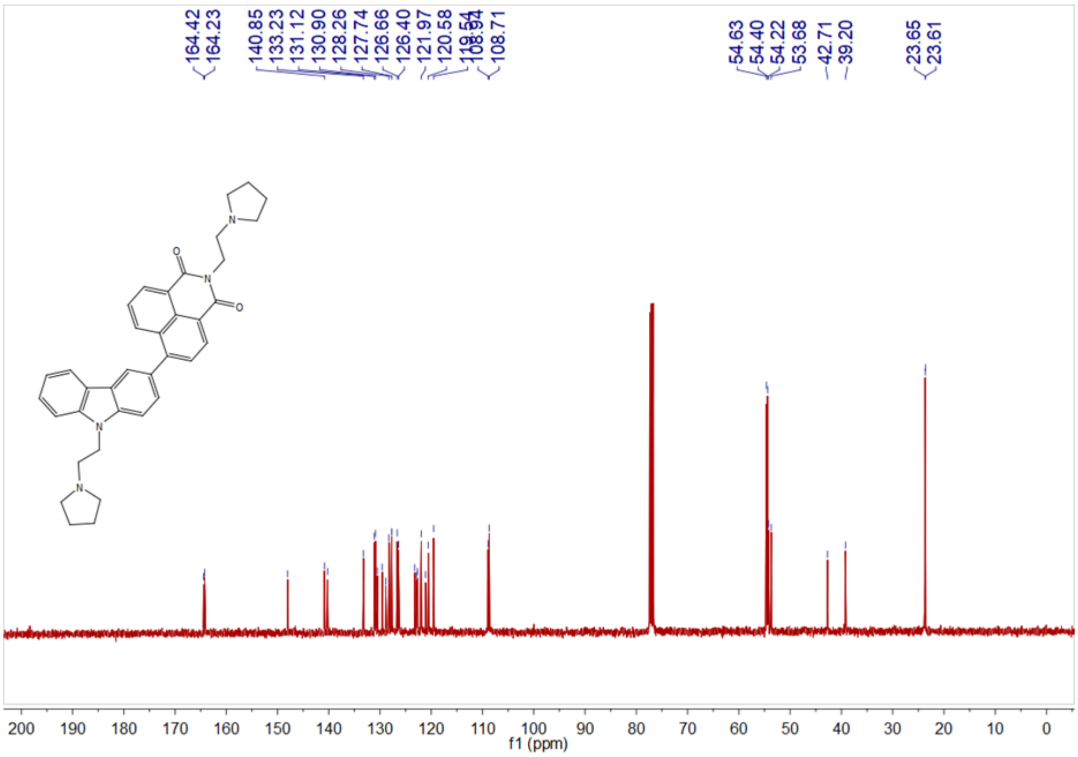

^1H and ^{13}C NMR of **G50**:

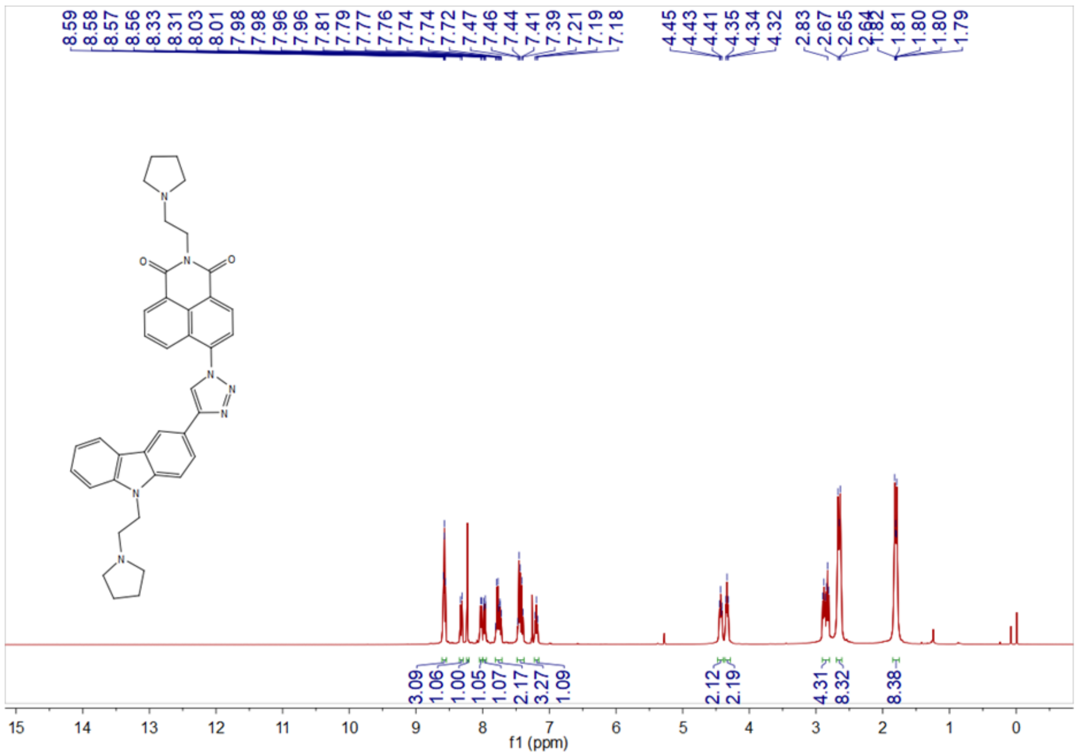

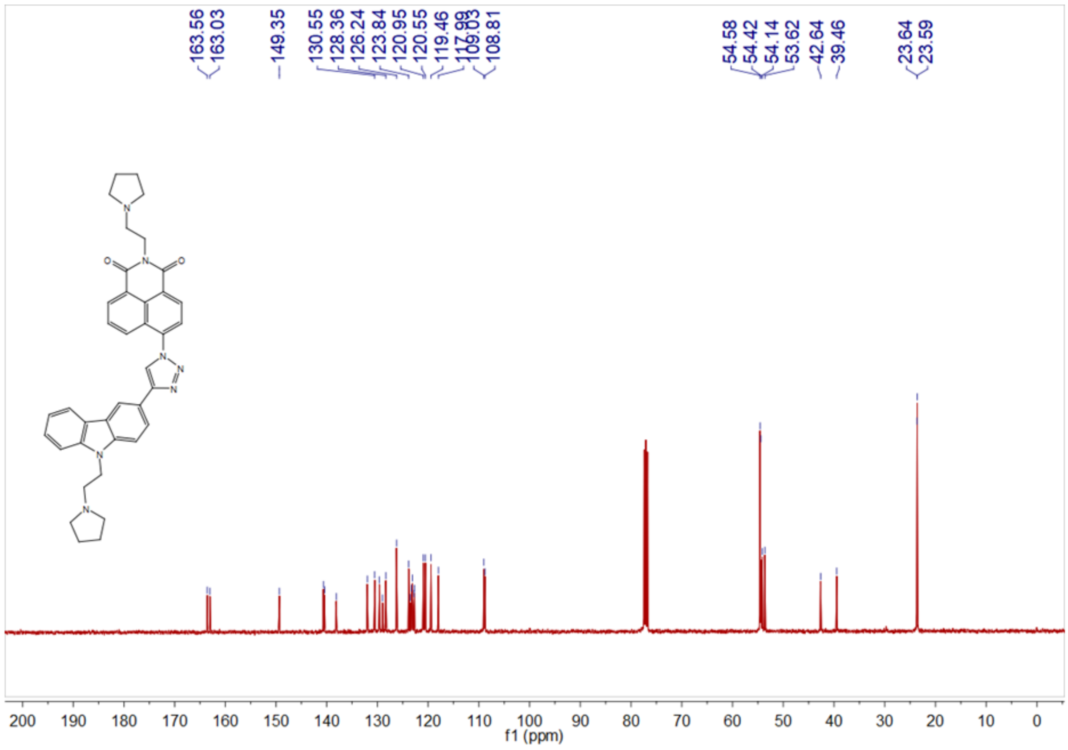

^1H and ^{13}C NMR of **G51**:

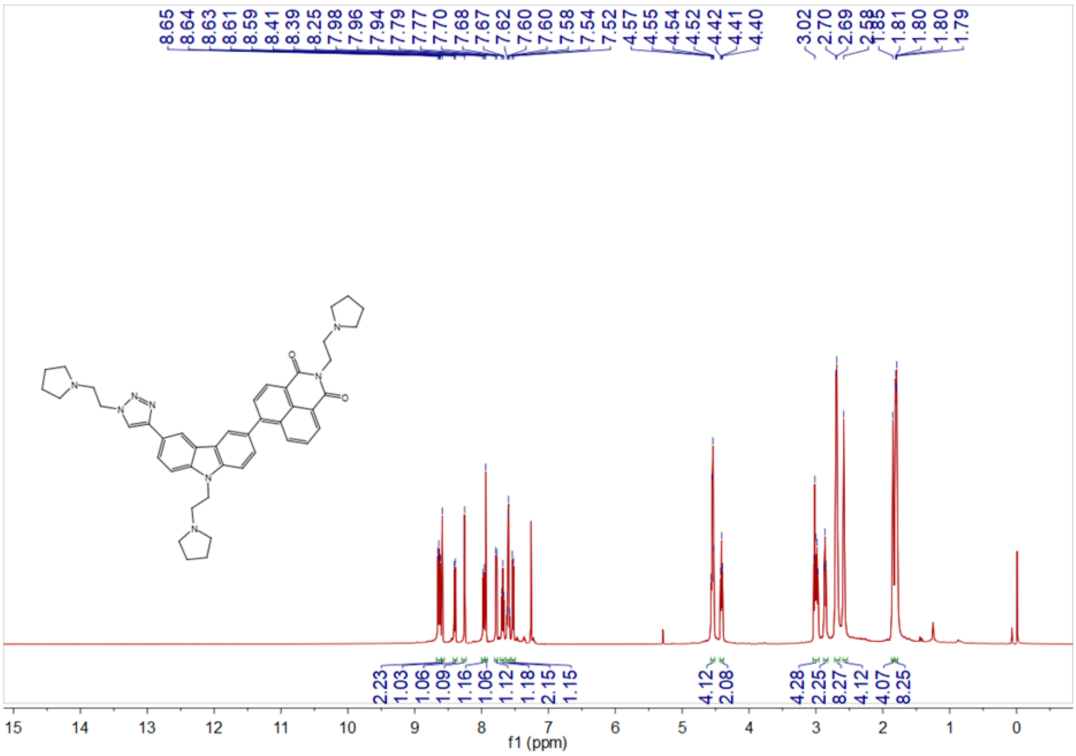

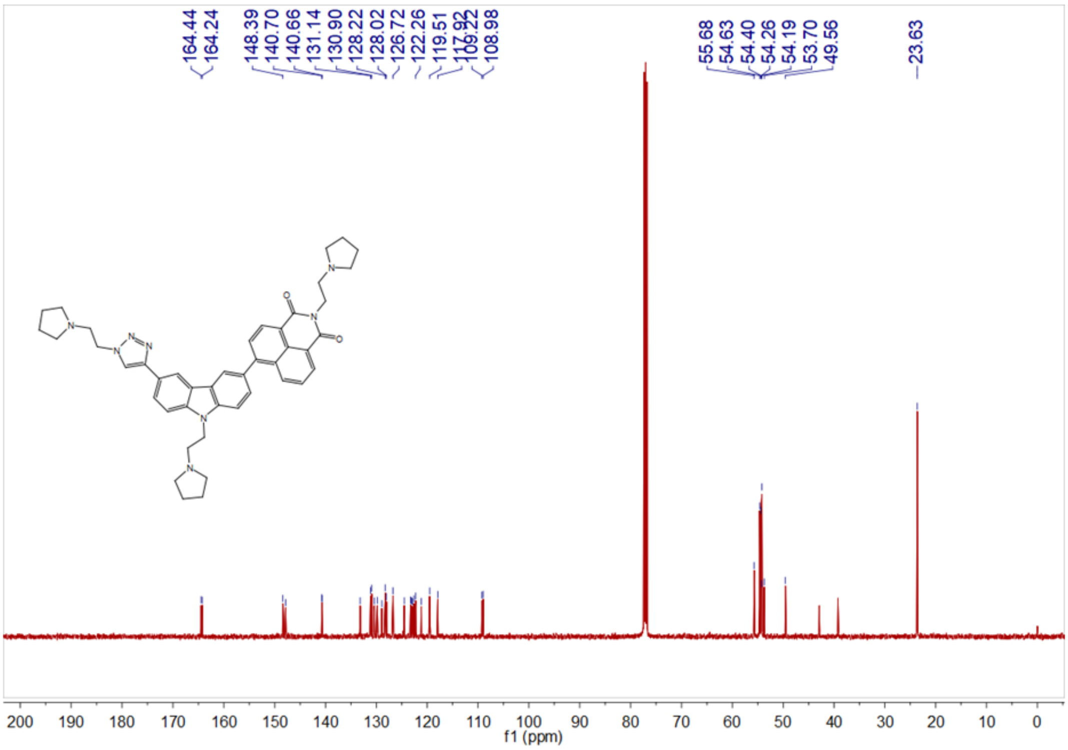

^{1}H and ^{13}C NMR of **G59**:

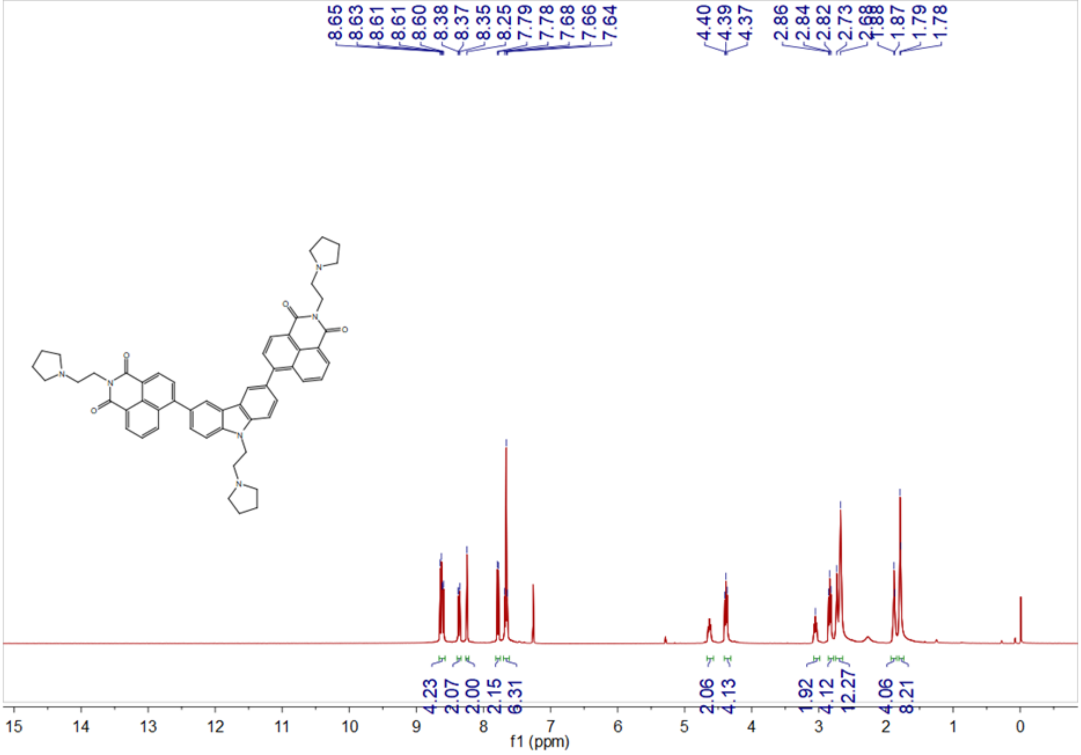

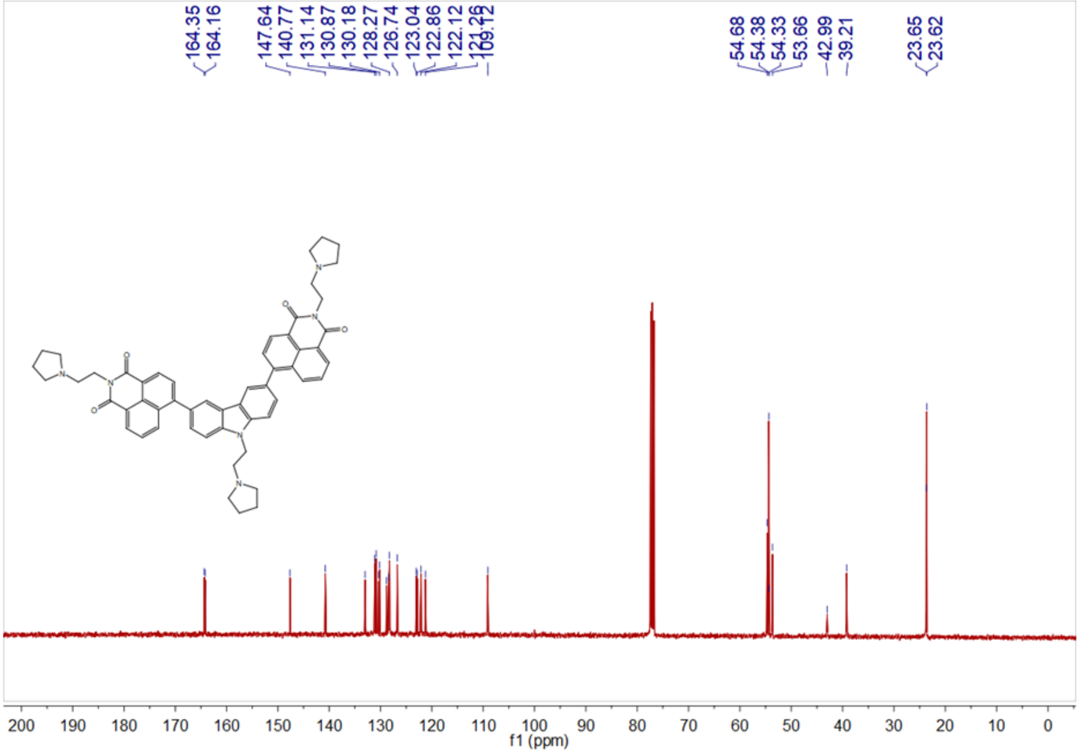

^{1}H and ^{13}C NMR of **G60**:

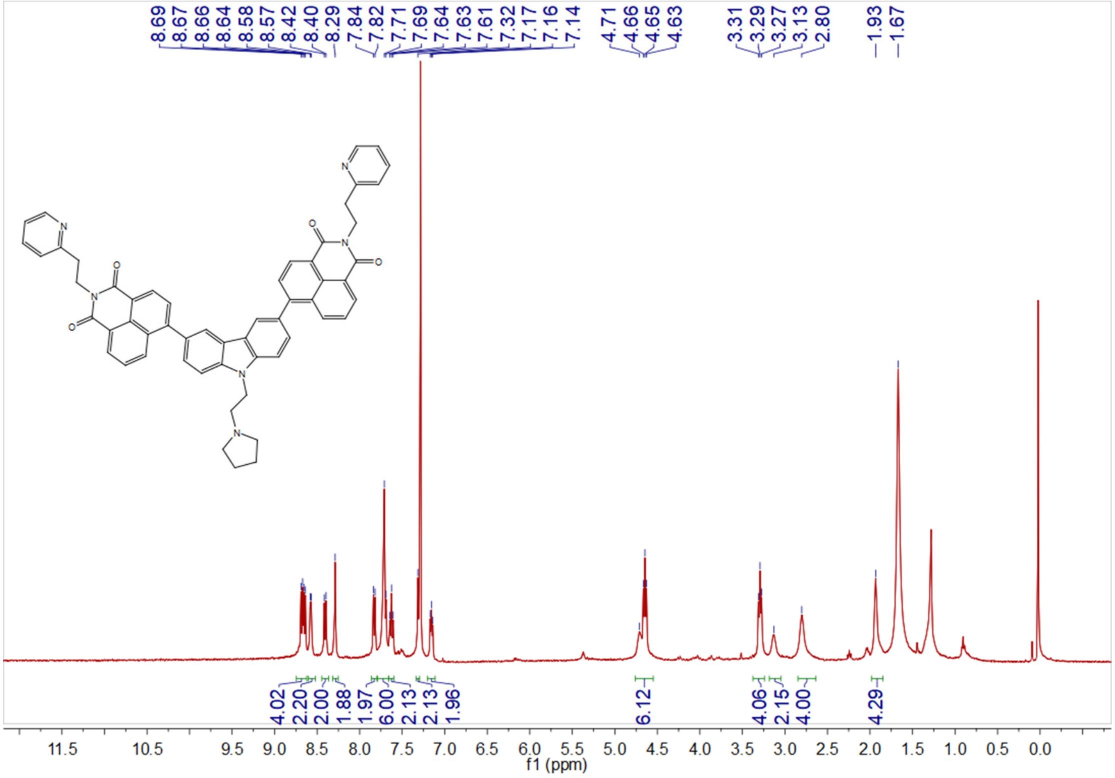

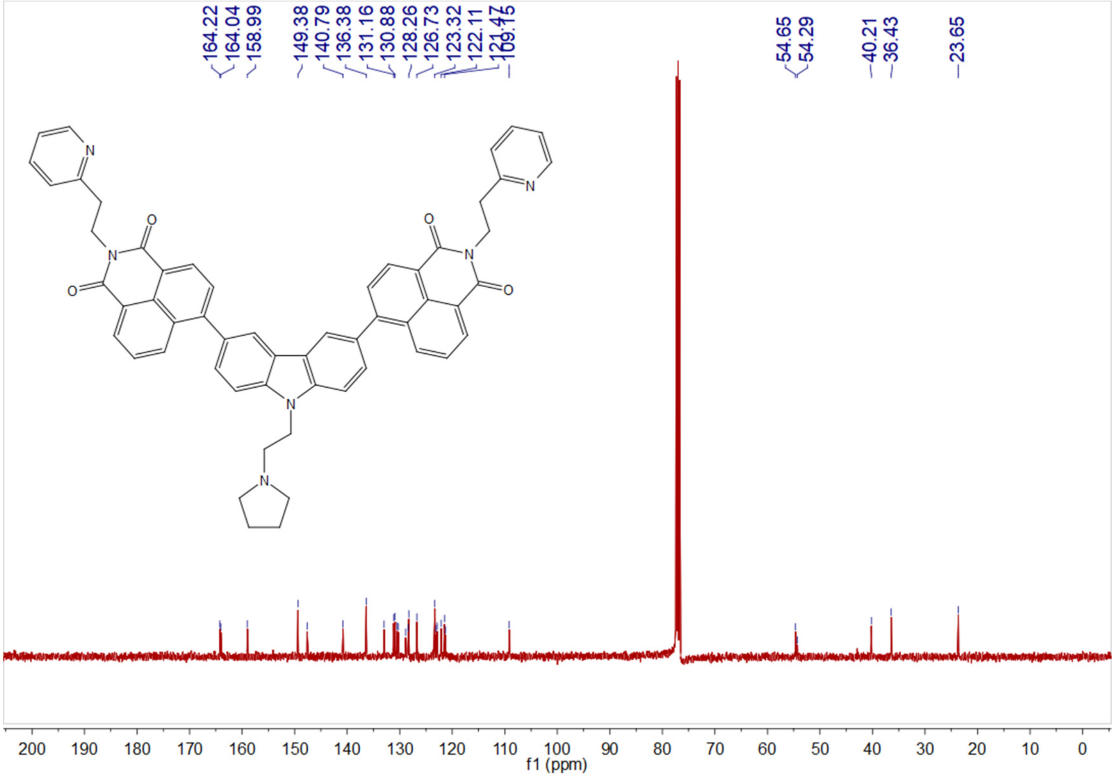

^{1}H and ^{13}C NMR of **G61**:

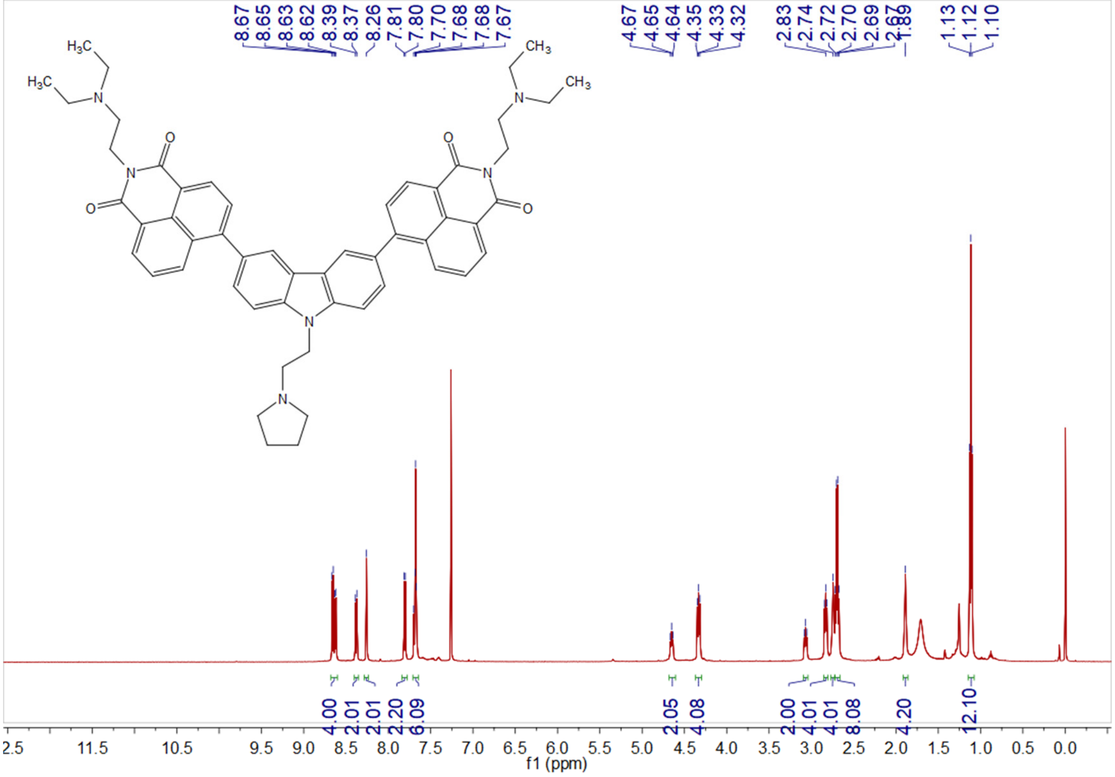

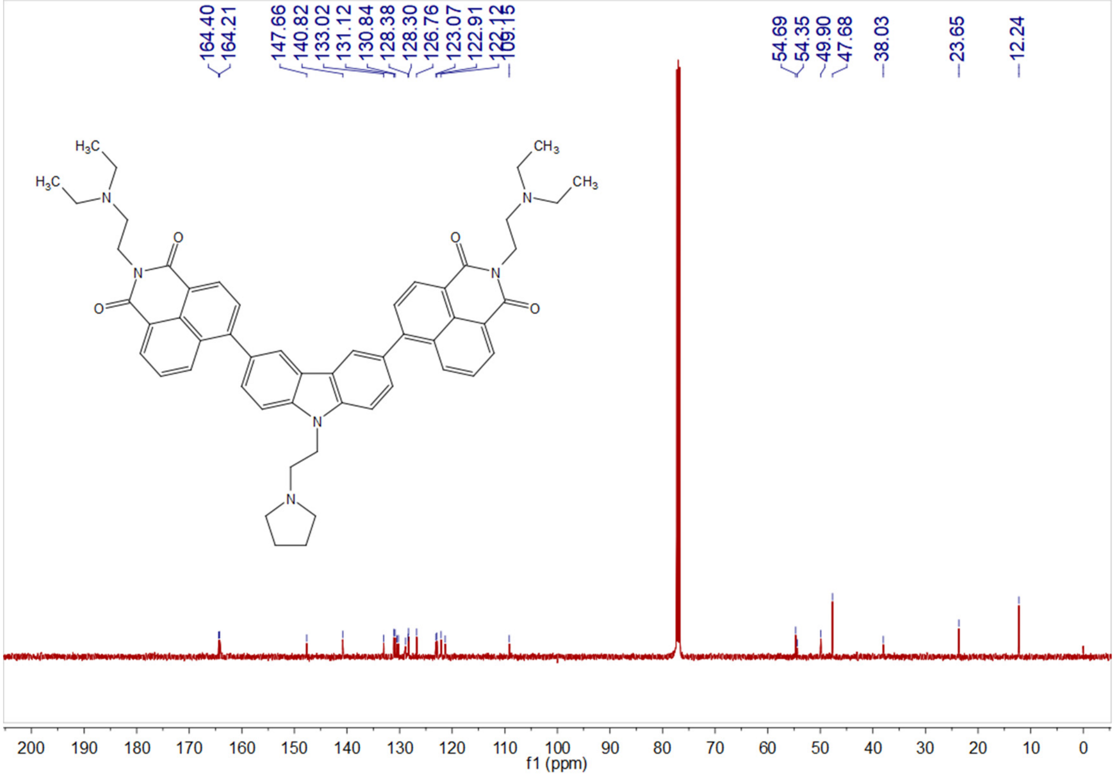

^{1}H and ^{13}C NMR of **G62**:

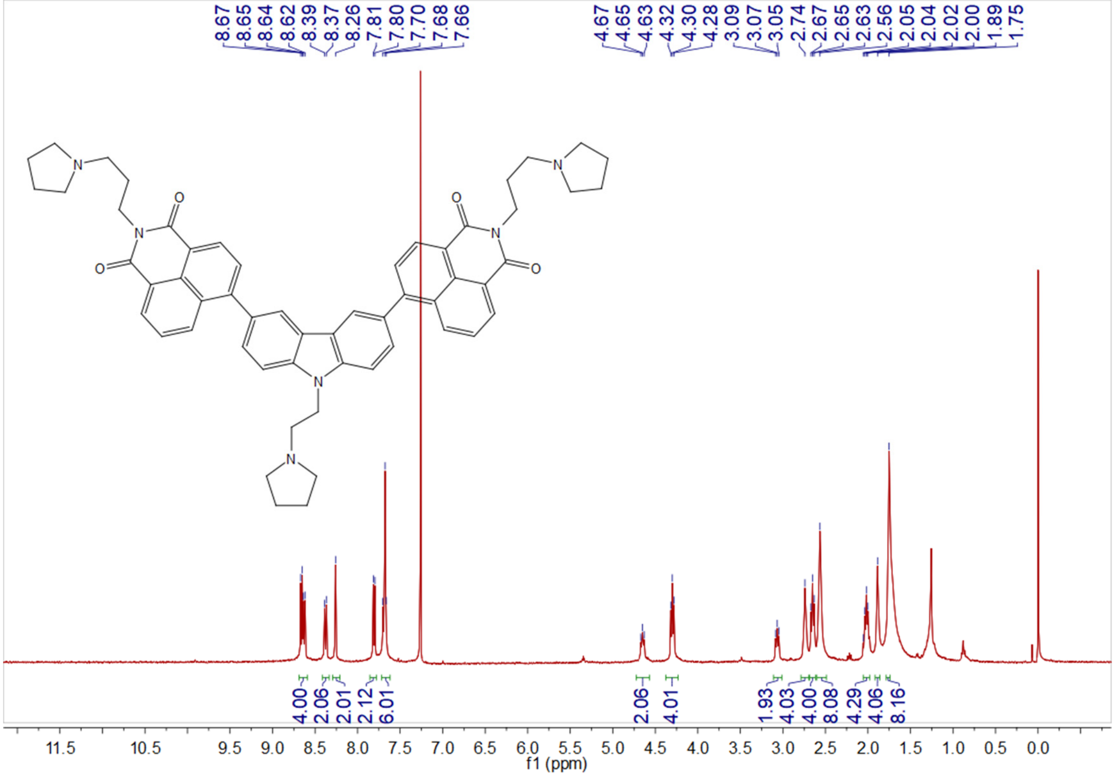

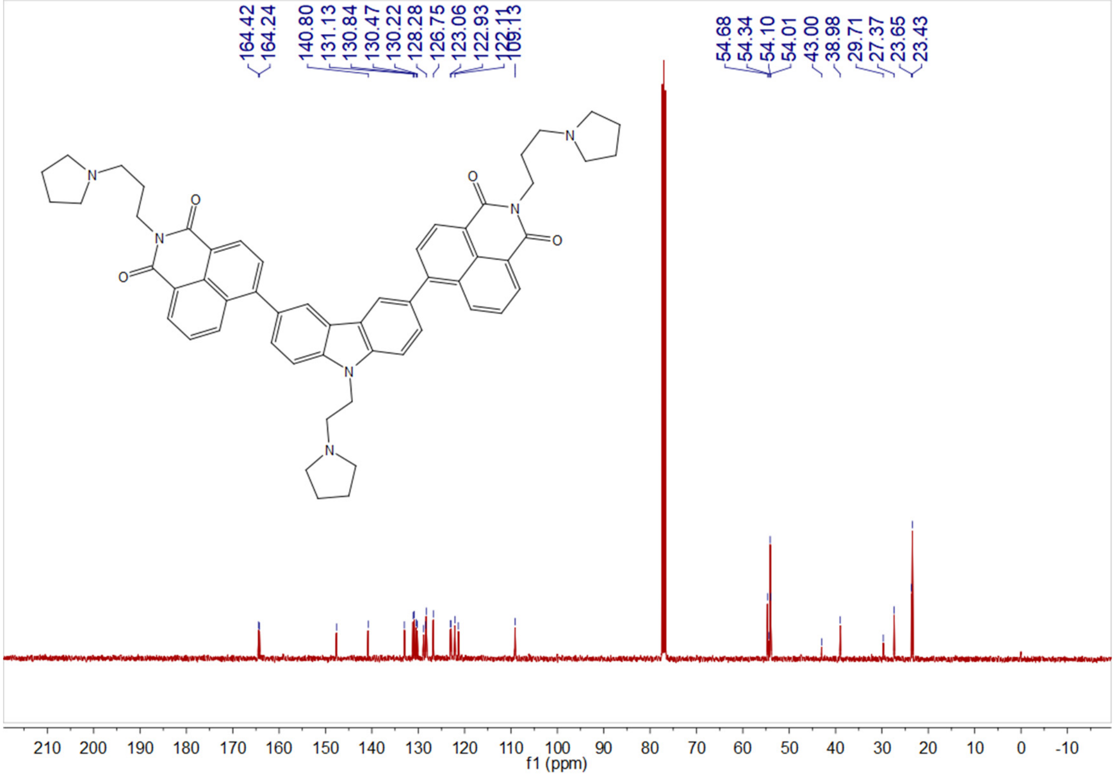

^{1}H and ^{13}C NMR of **G63**:

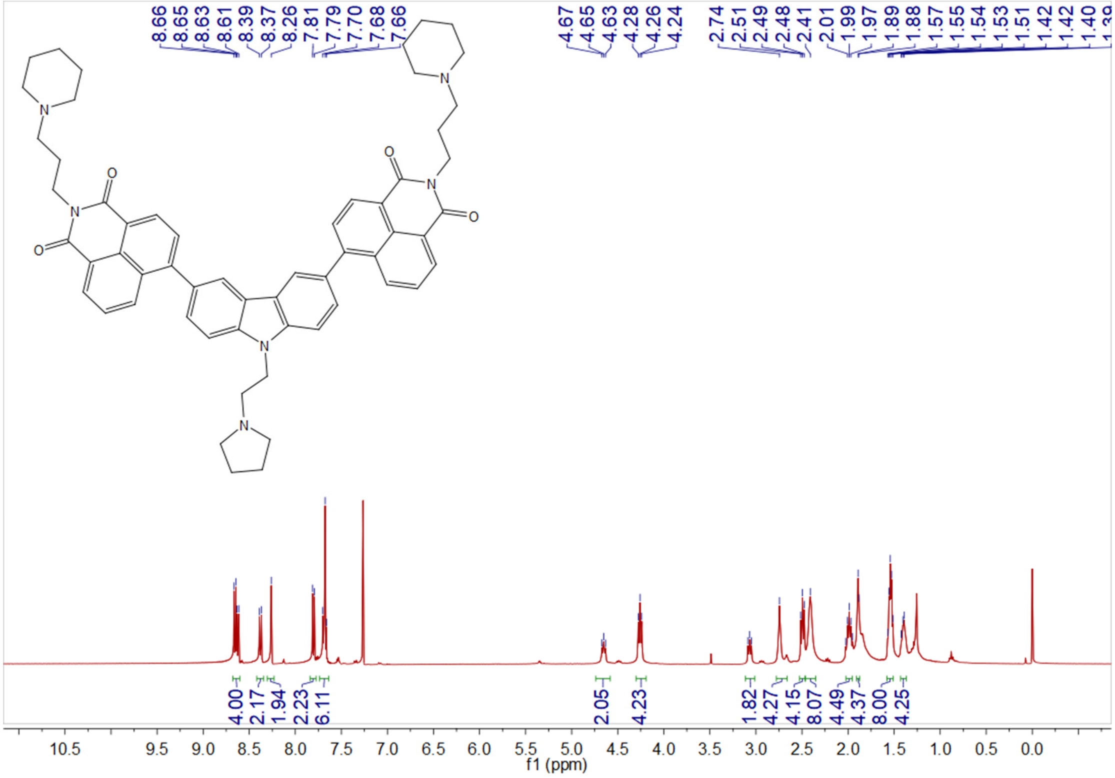

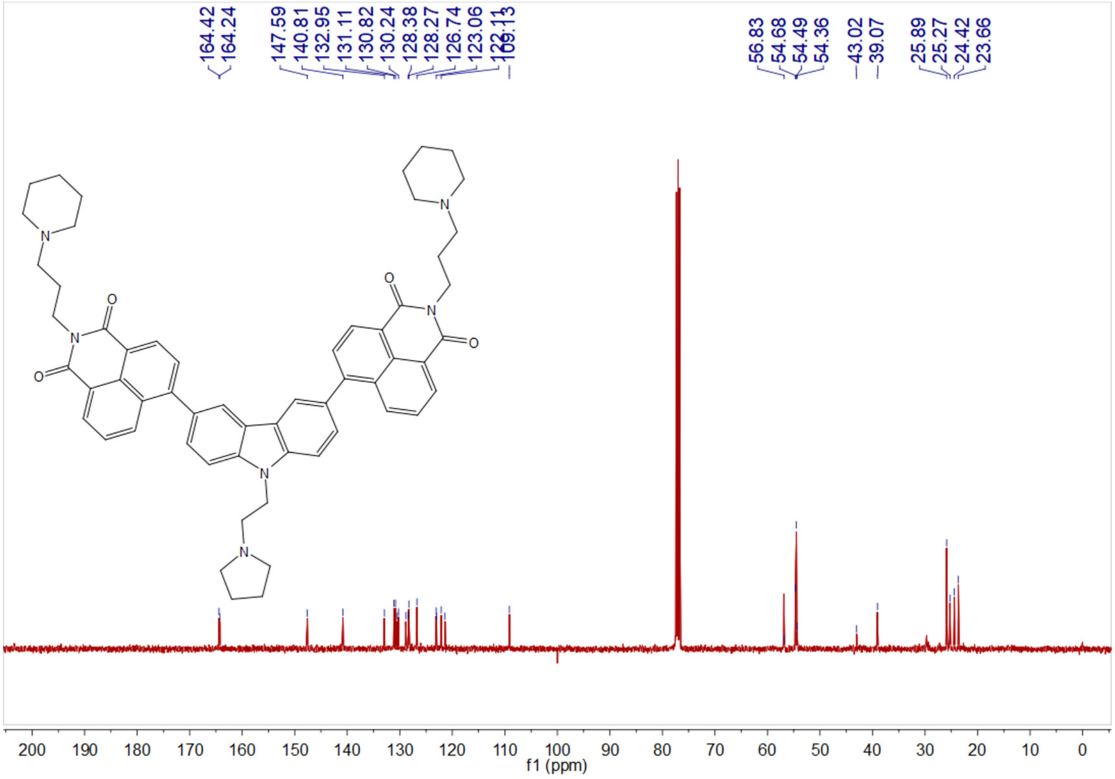

^{1}H and ^{13}C NMR of **G64**:

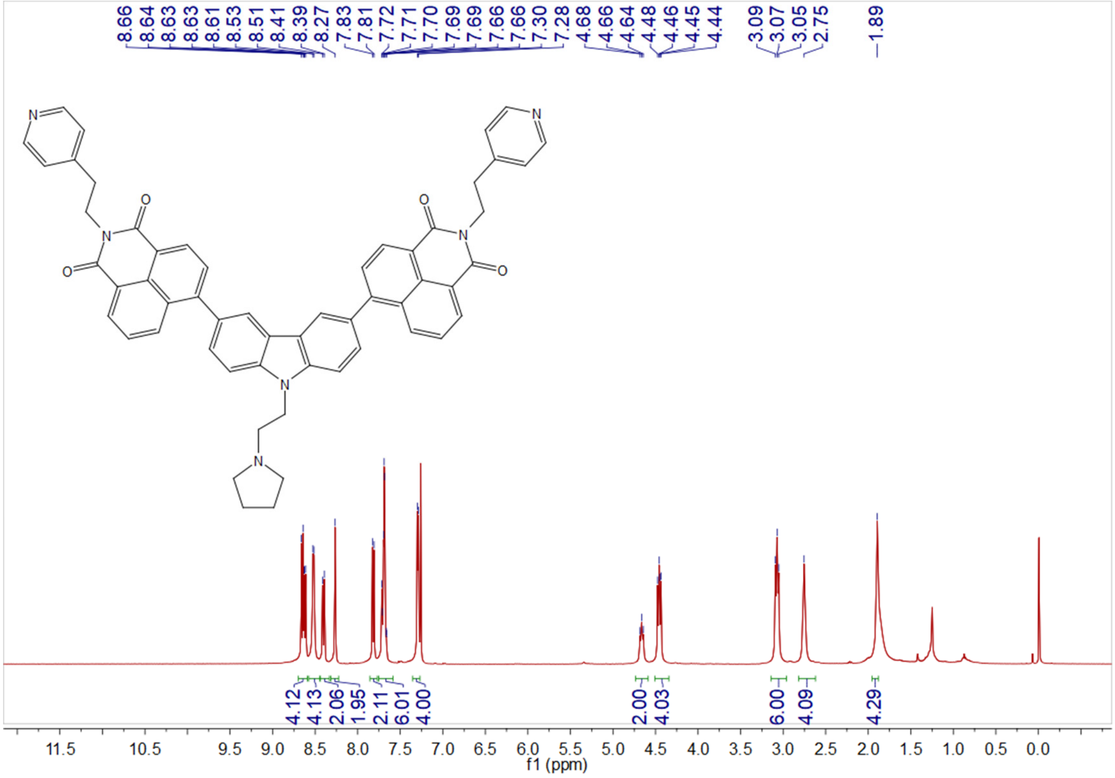

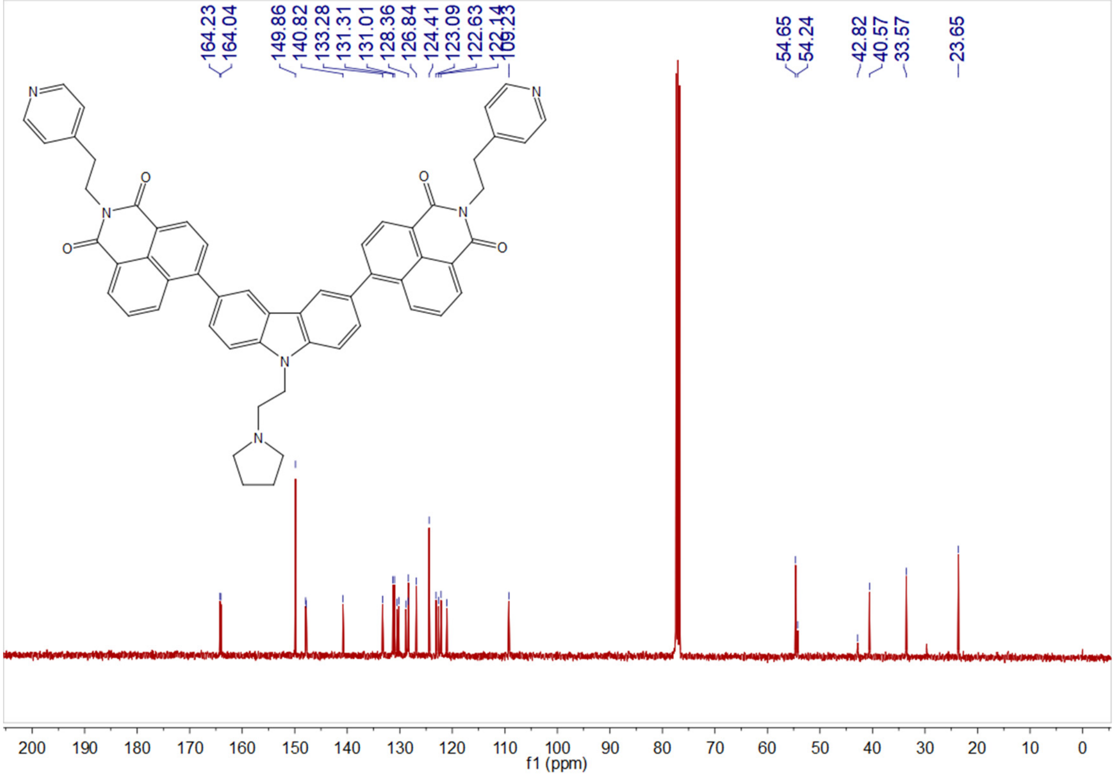

^{1}H and ^{13}C NMR of **G65**:

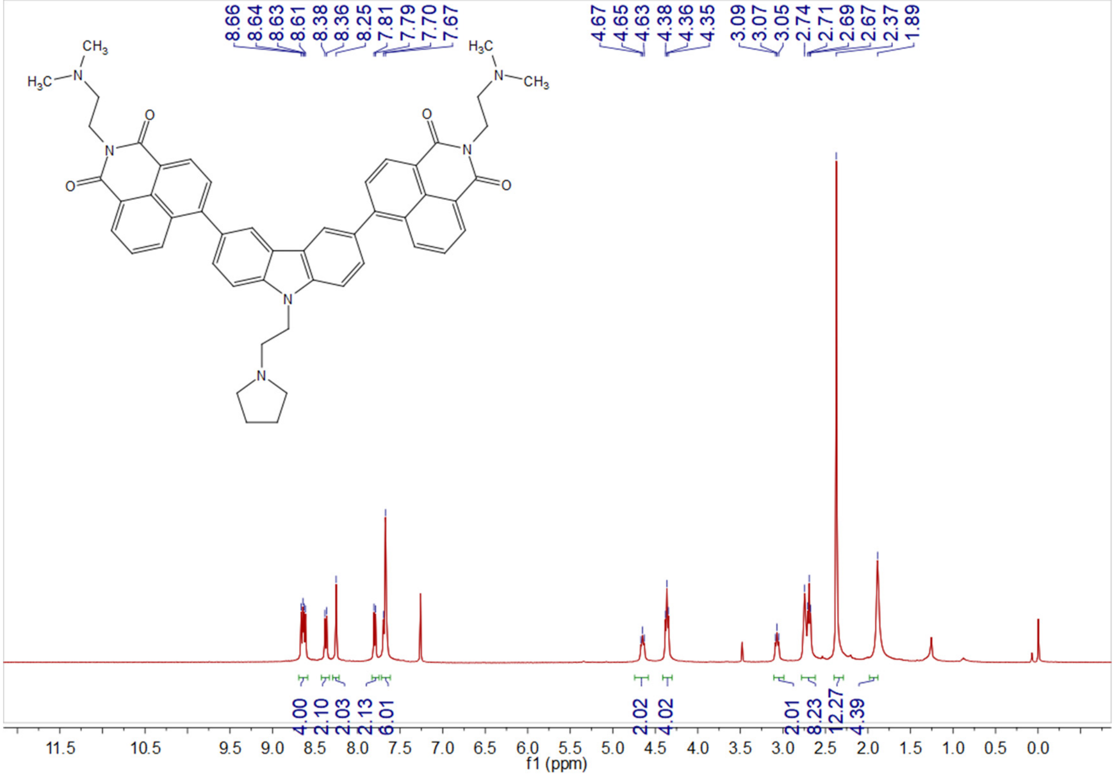

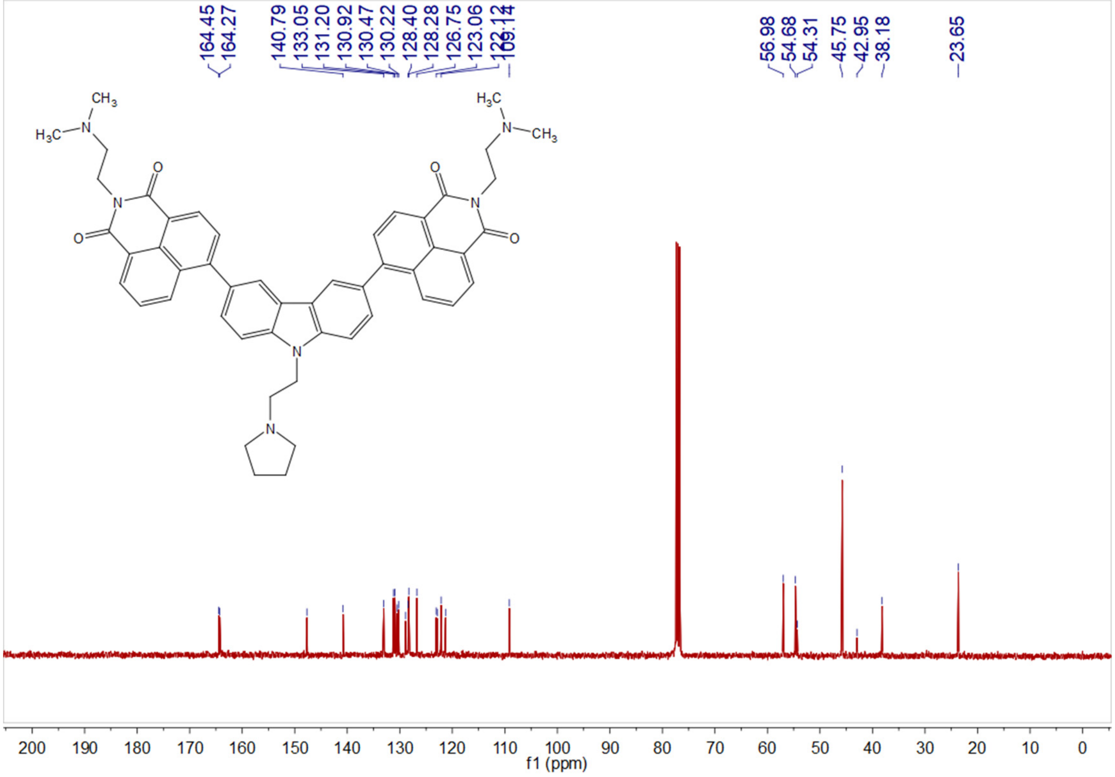

^{1}H and ^{13}C NMR of **G66**:

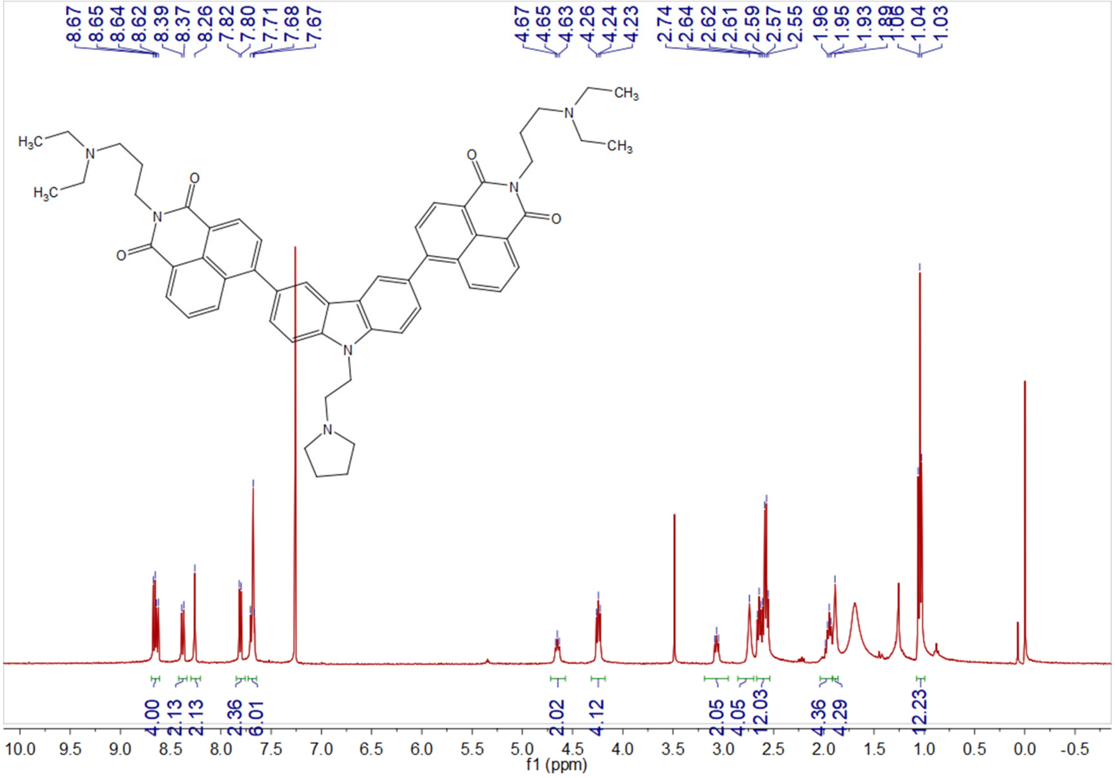

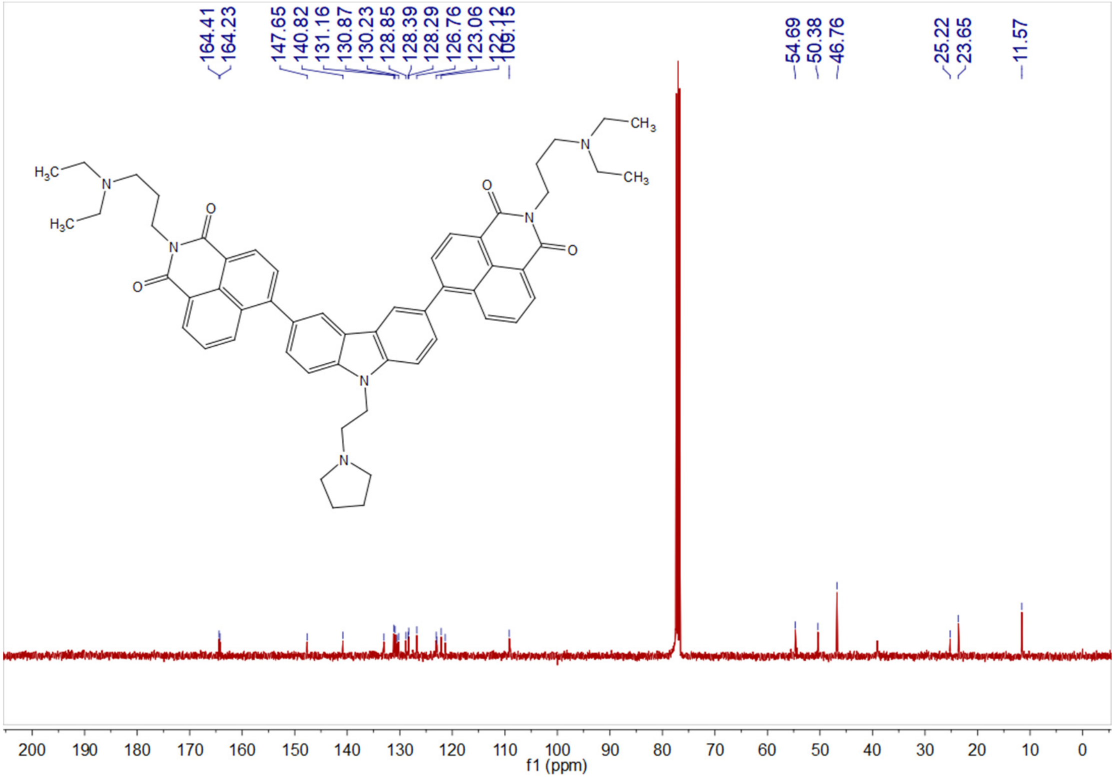

^{1}H and ^{13}C NMR of **G67**:

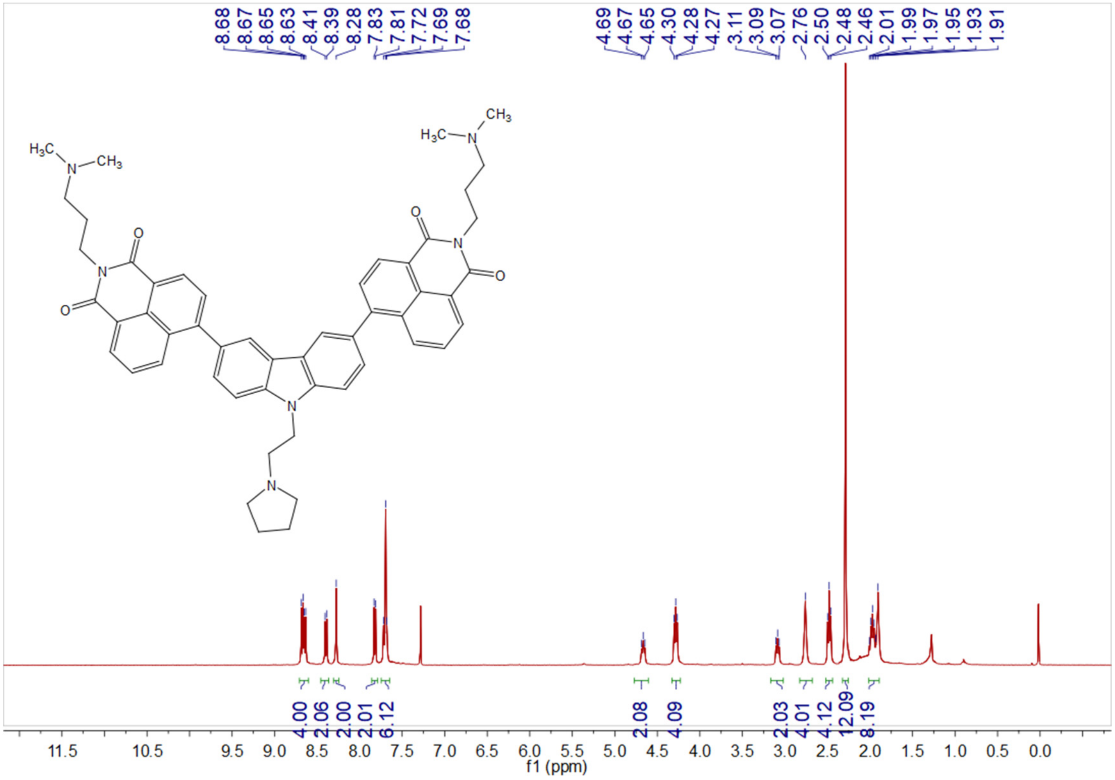

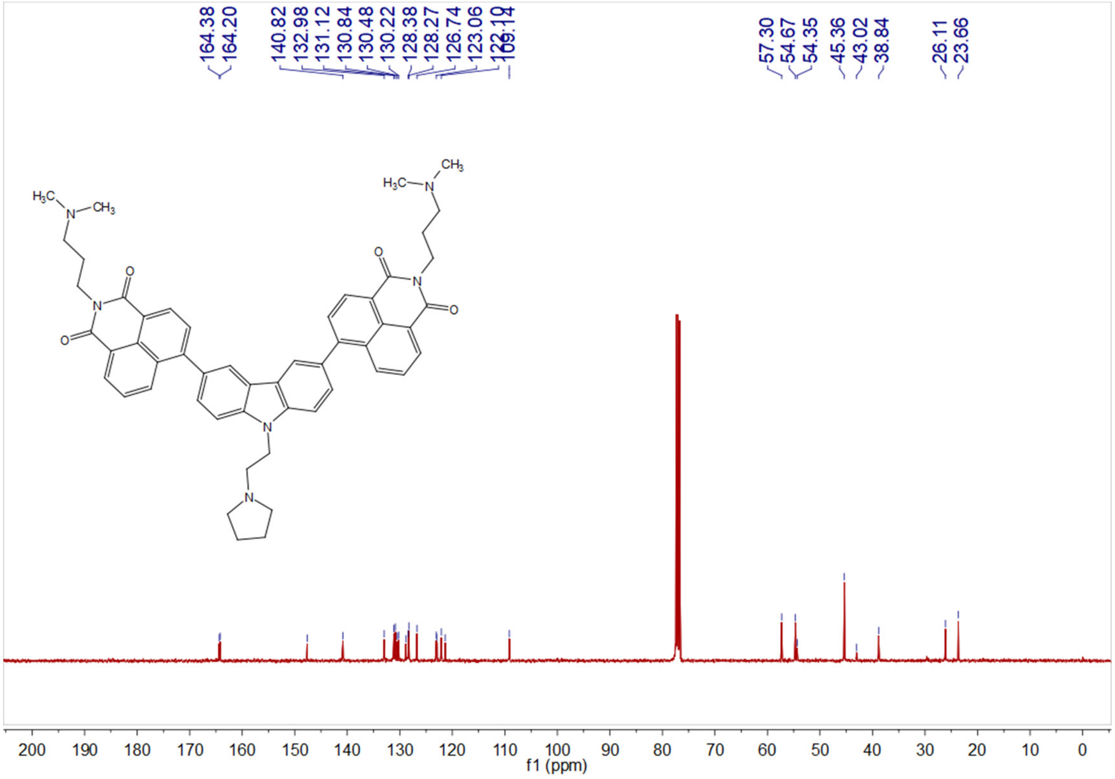

Supplement: Supplementary file 1 [file ijms-23-03872-s001.zip › ijms-1645306-supplementary.pdf]
